# Global groundwater contamination by geogenic fluoride

**DOI:** 10.1007/s10653-026-03187-8

**Published:** 2026-06-17

**Authors:** Shakir Ali, Pratibha Mishra, K. Brindha, Mélida Gutiérrez, Rakesh Kumar, Emmanuel Daanoba Sunkari, Patrick Kirita Gevera, Enn Karro, Johnbosco C. Egbueri, Reza Dehbandi, Rohana Chandrajith, Peiyue Li, Abu Reza Md. Towfiqul Islam, Seong-Taek Yun, Hullysses Sabino, Martínez Daniel Emilio, Alper Baba, Taimoor Shah Durrani, Vahab Amiri, Adnan Aqeel, Julian Ijumulana, Joshua Nosa Edokpayi, David Schafer, Lidia Razowska-Jaworek, Maria Teresa Alarcón-Herrera, Odsuren Batdelger, Ritusmita Goswami, Abida Farooqi, Alcaraz Emiliano Fabio, Yaşar Kemal Recepoğlu, Soraya Paz-Montelongo, Prosun Bhattacharya

**Affiliations:** 1 Department of Civil Engineering Science, Faculty of Engineering & the Built Environment, University of Johannesburg, Auckland Park, Kingsway Campus, Johannesburg, South Africa; 2 School of Geosciences, The University of Edinburgh, Edinburgh, EH8 9XP Scotland; 3 Department of Water Resources and Ecosystems, IHE Delft Institute for Water Education, Westvest 7, 2611 AX Delft, The Netherlands; 4 School of Earth, Environment, and Sustainability, Missouri State University, Springfield, USA; 5 Department of Biosystems Engineering, Auburn University, Auburn, AL 36849 USA; 6 Mining Engineering, Faculty of Integrated and Advanced Technology, Sir Padampat Singhania University, Udaipur, 313601 Rajasthan India; 7 Department of Chemical Sciences, Faculty of Science, University of Johannesburg, Auckland Park, P.O. Box 524, Johannesburg, 2006 South Africa; 8 Department of Civil Engineering, University of South Africa, [Florida Science Campus], Cnr Christian de Wet Road and Pioneer Avenue, Johannesburg, South Africa; 9 Department of Geology, Institute of Ecology and Earth Sciences, University of Tartu, Ravila 14a, 50411 Tartu, Estonia; 10 Department of Geology, Chukwuemeka Odumegwu Ojukwu University, Uli, Nigeria; 11 Research Management Office (RMO), Chukwuemeka Odumegwu Ojukwu University, Anambra, Nigeria; 12 Department of Chemical Engineering, University of Science and Technology of Mazandaran, Behshahr, Iran; 13 School of Geography, Earth and Environmental Sciences, University of Birmingham, Edgbaston, Birmingham, B15 2TT UK; 14 Department of Geology, Faculty of Science, University of Peradeniya, Peradeniya, Sri Lanka; 15 School of Water and Environment, Chang’an University, No. 126 Yanta Road, Xi’an, 710054 Shaanxi China; 16 Disaster Science and management, Begum Rokeya University, Rangpur, 5400 Bangladesh; 17 Department of Earth and Environmental Sciences, Korea University, Seoul, 02841 South Korea; 18 Geography Department, Geosciences Institute, Universidade Federal Fluminense, Rio de Janeiro, Brazil; 19 Instituto de Geología de Costas y del Cuaternario (U.N. Mar del Plata–CIC BA), Instituto de Investigaciones Marinas y Costeras (CONICET-U.N. Mar del Plata), Mar del Plata, Argentina; 20 Department of the International Water Resources, Izmir Institute of Technology, Izmir, Türkiye; 21 Department of Environmental Sciences, Balochistan University of Information Technology Engineering and Management Sciences (BUITEMS), Quetta, Pakistan; 22 Department of Geology, Yazd University, Yazd, Iran; 23 Department of Earth & Environmental Sciences, Sana’a University, Sana’a, Yemen; 24 DAFWAT Research Group, Department of Water Resources Engineering, College of Engineering and Technology, University of Dar Es Salaam, Dar Es Salaam, Tanzania; 25 Geospatial Data Sciences and Technology (Geomatics) Section, Department of Transportation and Geotechnical Engineering, College of Engineering and Technology, University of Dar Es Salaam, Dar Es Salaam, Tanzania; 26 Water and Environmental Management Research Group, Faculty of Science, Engineering and Agriculture, University of Venda, Limpopo Province, Thohoyandou, 0950 South Africa; 27 Department of Water and Environmental Regulation, Perth, WA Australia; 28 Polish Geological Institute-National Research Institute, Warsaw, Poland; 29 Centro de Investigaciones de Materiales Avanzados, Dgo.34147, Durango, México; 30 Institute of Geography and Geoecology, Mongolian Academy of Sciences, Ulaanbaatar, Mongolia; 31 Centre for Ecology Environment and Sustainable Development, Tata Institute of Social Sciences, Guwahati, Assam India; 32 Environmental Hydro Geochemistry Lab, Department of Environmental Sciences, Quaid-I-Azam University, Islamabad, Pakistan; 33 Department of Chemical Engineering, Izmir Institute of Technology, Izmir, Türkiye; 34 Department of Chemical Engineering, Ege University, Izmir, Türkiye; 35 Área de Toxicología, Universidad de La Laguna, 38071 La Laguna, Tenerife, Islas Canarias Spain; 36 KTH-International Groundwater Arsenic Research Group, Department of Sustainable Development, Environmental Science and Engineering, KTH Royal Institute of Technology, Teknikringen 10B, 114 28 Stockholm, Sweden; 37 Centre of Excellence in Environmental Science and Sustainability, Sir Padampat Singhania University, Udaipur, 313601 Rajasthan India

**Keywords:** Fluoride, Chronic fluorosis, Geogenic contamination, Defluoridation, Water–rock interactions, Public health, Global risk

## Abstract

Fluoride (F¯) is a global geogenic contaminant in groundwater, occurring at elevated concentrations across almost every continent. While optimal fluoride intake benefits dental health, levels exceeding the WHO safe limit (1.5 mg L^−1^) can cause dental fluorosis and even severe skeletal fluorosis, with drinking water being the primary exposure pathway. Understanding the global occurrence and geochemistry of fluoride in groundwater is essential for minimizing associated health risks. Therefore, this study reviews the incidence, distribution, mobilization mechanisms of fluoride in groundwater, and identifies the key knowledge gaps related to fluoride contamination worldwide. The study presents a large-scale synthesis of contaminated aquifers, cost-effective defluoridation methods, socio-economic challenges, and strategies for safe drinking water supply. This was done by adopting the PRISMA 2020 systematic review framework with review period between 1953 and 2025. Findings confirm that fluoride contamination is prevalent in Asia, Africa, the Middle East, Europe, and the Americas, driven mainly by fluorine-bearing minerals in aquifers. Key factors intensifying the contamination includes hydrogeochemical conditions such as high pH, Na–HCO_3_ water types, and low calcium (Ca^2+^) concentrations. Identified hotspot are the East African Rift Valley, Indo-Gangetic plains, northern China, and volcanic or geothermal regions in Mexico and Türkiye, with localized anomalies observed in Estonia, Poland, and Australia. Anthropogenic inputs contribute in some localized areas, although their impact is minimal. The fluoride levels are particularly high in arid and semi-arid regions, where evaporation and prolonged water–rock interaction accelerate fluoride mobilization. Despite decades of research, monitoring gaps persist, and mitigation remains challenging, especially for rural communities who are entirely dependent on untreated groundwater. This study recommends prioritizing efforts on scalable, cost-effective solutions to provide fluoride-safe water, particularly in areas where populations are largely vulnerable.

**Supplementary Information:**

The online version contains supplementary material available at 10.1007/s10653-026-03187-8.

## Introduction

Fluoride (F¯) is a naturally occurring constituent commonly found in groundwater and is considered as a contaminant when its concentration exceeds the World Health Organization (WHO) guideline of 1.5 mg L^−1^ (WHO, 2011). Although not usually classified as an essential nutrient, fluoride has dental benefits and may contribute to bone development (WHO, 2019). Drinking water is the primary source of fluoride exposure, followed by food and dental products. Recent global hazard maps estimate that roughly 180 million people are at risk of fluoride-related health issues, with the most affected regions in developing countries where impacts extend beyond dental caries due to lack of access to safe drinking water (Podgorski & Berg, 2022). Elevated fluoride concentrations occur across diverse climatic and geological conditions, particularly in Asia and Africa, where hydrogeologic and climatic conditions favour enrichment (Podgorski & Berg, 2022; Podgorski et al., 2018; Habiyakare et al., 2025). A review indicated that more than 100 countries are affected and suggests that exposures may exceed 200 million individuals, with hotspots in arid and semi-arid regions (Shaji et al., 2024). Terrains containing granitoids, alkaline intrusions, geothermal systems, and volcanic rocks host fluorine-bearing minerals and promote prolonged water–rock interaction (Shaji et al., 2024). Geochemically, Na–HCO_3_ type waters with low calcium (Ca^2+^) stabilize dissolved fluoride and inhibit fluorite precipitation, a pattern observed in crystalline and volcanic aquifers from East Africa to parts of West Africa and western North America (Podgorski & Berg, 2022). Although minimal intake of fluoride has dental benefits, concentrations above 1.5 mg L^−1^ are linked to dental fluorosis, and chronic intake of ≥ 6 mg/day increases the risk of skeletal fluorosis, underscoring the need for monitoring and targeted mitigation in vulnerable regions (Podgorski & Berg, 2022; Shaji et al., 2024).

Globally, fluoride occurs in various rocks and sediments in variable proportions. Numerous studies have documented the possible mobilization of fluoride from rocks, soils, and sediments into water (Ali et al., 2016; Brindha & Elango, 2013). Regions naturally enriched in fluoride, often referred to as “fluoride belts,” have been identified by researchers (e.g., Chowdhury et al., 2019; Table 1). However, elevated fluoride levels in groundwater have also been reported in countries outside these belts. This suggests that elevated fluoride is not solely restricted to these geogenic zones, anthropogenic sources may also play a minor role.

**Table 1:** Fluoride belts and associated tectonic zones (modified after Chowdhury et al., 2019)

Belt	Country	Tectonic zone
1	Eritrea, Djibouti, Ethiopia, Kenya, Tanzania, Uganda, Rwanda, Burundi, DRC, and Malawi	East African Rift Zone
2	Egypt, Libya, Algeria, Morocco, and parts of Western Sahara	North African Mobile Belt
3	Türkiye, Iran, Afghanistan, Pakistan, India, and parts of China	Tethyan Mobile Belt
4	Ghana, Nigeria, and Mauritania	West African Craton
5	Western USA (Cascade Range), Mexico, Andes Mountains (Colombia, Ecuador, Peru, Chile, Bolivia), Japan, Philippines, Indonesia	Volcanic Belt

Anthropogenic activities, such as the extensive use of fluoride-based fertilizers, industrial fluorine applications, and paints, can also contribute to elevated fluoride levels, though their impact is often insignificant (Ali et al., 2023; Brindha & Elango, 2011; Nordstrom & Smedley, 2022).

Fluoride exhibits ambivalent effects, with both lower and upper permissible limits (0.5–1.5 mg L^−1^). Fluoride is non-toxic at the lower limit and is essential in moderate concentrations for proper tooth enamel formation and bone mineralization (Ali et al., 2016). Exceeding the maximum permissible limit over prolonged periods can be detrimental to human health. Early effects often manifest as dental fluorosis, which may progress to irreversible skeletal fluorosis and other health issues. While the effects of fluoride on teeth and bones have been well-known and studied since the 1900s (Medjedovic et al., 2015; Rango et al., 2012), its neurological effects such as impacts on intelligence quotient (IQ), learning, memory, and attention span, particularly in children, have gained attention only in the last two decades (Godebo et al., 2023; Goodman et al., 2022). Based on health risks associated with fluoride concentrations, six broad categories or risk levels can be identified, as listed in Table 2.

**Table 2:** Classification of fluorosis risk levels in drinking water (Ali et al., 2016; Aqeel et al., 2017)

Fluoride range (mg/L)	Symptoms/ diagnosis and impacts	Risk category
< 0.5	Dental caries	Low
0.5 – 1.5	Required for human well-being	Optimum level
1.5 – 2.0	Dental fluorosis	Mild
2.0 – 4.0	Dental fluorosis, skeletal fluorosis, and chronic kidney diseases	Moderate
4.0 – 10.0	Dental fluorosis, skeletal fluorosis, and nephrotoxicity	Severe (high)
> 10.0	Crippling fluorosis and neurological disorders	Extremely severe (very high)

Daily intake of water is also an important key factor in fluoride ingestion, which may lead to adverse health effects (Fantong et al., 2010; WHO, 1994, 2017). Where daily water consumption rates are high, maximum fluoride levels for drinking water should be significantly lower than the maximum permissible limit (1.5 mg L^−1^) to avoid excessive fluoride ingestion (Fantong et al., 2010; WHO, 1994). This is especially true in hot dry climates. Consequently, lower maximum fluoride levels for hot dry climates need to be further modified, especially considering that fluoridated toothpaste is easily accessible and acts as a supply source for the prevention of dental caries (WHO, 1994, 2017).

Removal of fluoride from groundwater has been a significant concern for environmental and human health safety. Various conventional and advanced technologies are available for effective fluoride removal, including coagulation, adsorption, electrochemical methods, ion exchange, membrane distillation, nanofiltration, precipitation, and reverse osmosis (Kut et al., 2016; Mohapatra et al., 2009). Defluoridation techniques have demonstrated success rates of 70 to 99% (Dar & Kurella, 2023; Fadaei, 2021). However, the biggest challenges, especially in rural communities, include the high cost of materials, operation, and maintenance, generation of undesirable by-products, limited technical expertise, and low public awareness towards defluoridation techniques (Yami et al., 2018).

Many case studies on groundwater fluoride across the globe provide valuable insights but do not offer comprehensive national or regional assessments. Independent studies often report fluoride concentrations in localized areas, yet coordinated efforts to synthesize these findings are limited. This study addresses this gap by integrating existing data to provide a holistic understanding of global fluoride exposure and its management. The study presents fluoride contamination data from 24 countries: Argentina, Australia, Bangladesh, Brazil, China, Colombia, Estonia, Ethiopia, Ghana, India, Iran, Kenya, Malawi, Mongolia, Mexico, Nigeria, Pakistan, Poland, South Korea, Sri Lanka, Tanzania, Türkiye, the USA, and Yemen. In many of these countries, a significant portion of the population is unintentionally consuming fluoride-contaminated groundwater and largely exposed to risk of fluorosis**.** Therefore, this study aims to document global fluoride occurrences, investigate mobilization mechanisms, identify knowledge gaps, and discuss cost-effective defluoridation solutions in the targeted countries.

## Methodology adopted for literature collection

The literature collection began with clearly defined objectives focused on fluoride contamination in groundwater. Relevant articles were gathered from various academic databases using appropriate keywords related to fluoride contamination in groundwater. Priority was given to articles published in peer-reviewed international journals; however, high-quality peer-reviewed articles from national journals were also considered. Studies were selected based on their scientific merit and relevance to fluoride contamination. Articles and reports published in languages other than English were also included, as local sources often provide important region-specific fluoride data. In such cases, authors proficient in the respective languages applied their expertise to accurately collect and incorporate the information. Data for map preparation were obtained from multiple sources and cleaned to generate maps for visualization and analysis of the spatial distribution of fluoride contamination in the selected countries using GIS software.

**Fig. 1:**
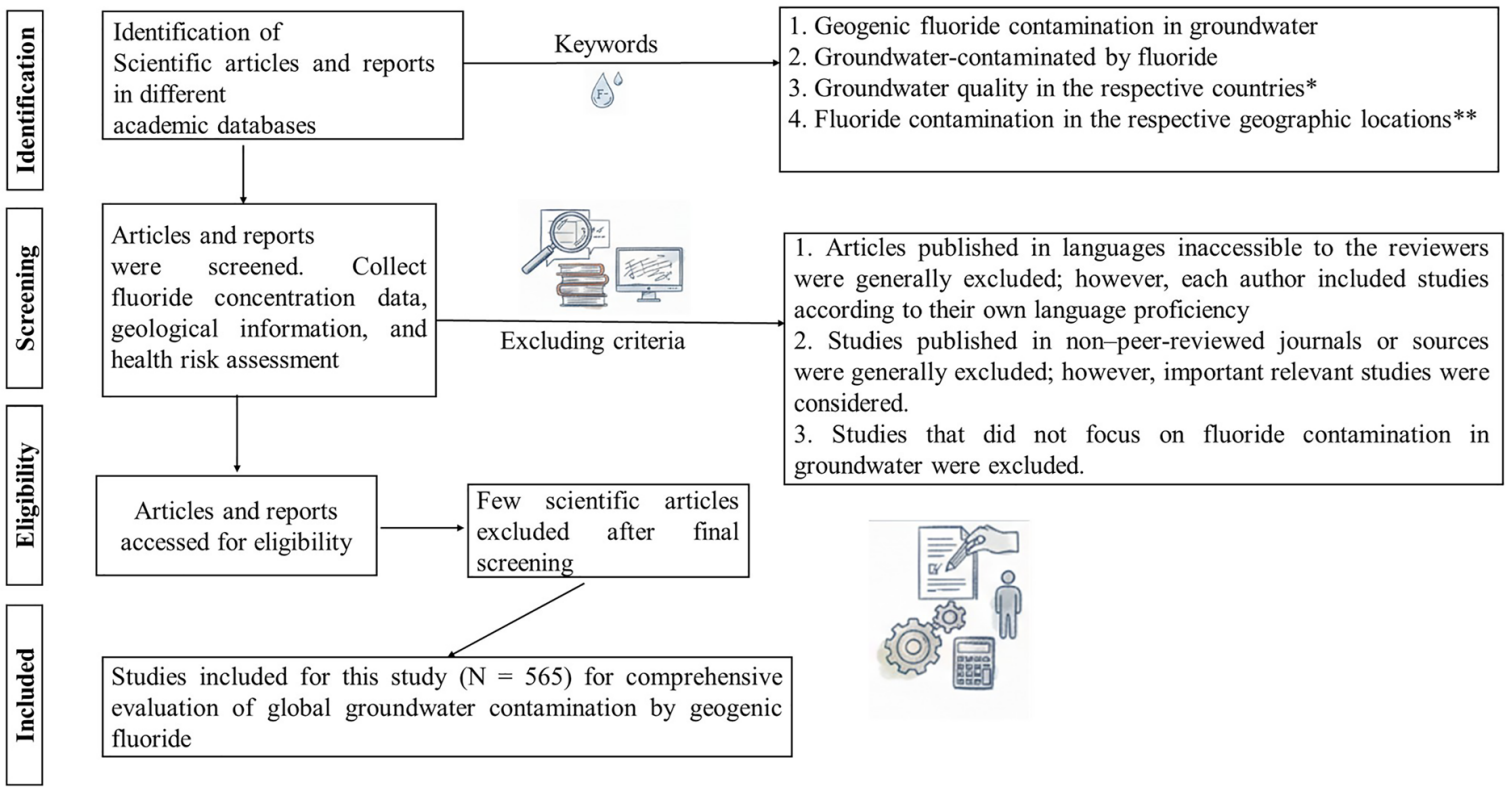
Flowchart illustrating the PRISMA methodology adopted in this study. (^*^The countries are Argentina, Australia, Bangladesh, Brazil, China, Colombia, Estonia, Ethiopia, Ghana, India, Iran, Kenya, Malawi, Mongolia, Mexico, Nigeria, Pakistan, Poland, South Korea, Sri Lanka, Tanzania, Türkiye, the USA, and Yemen. ^**^The geographical localities are South Asia, East Asia, the Middle East, Africa, North America, South America, Europe, and Oceania)

Defining a strict time frame for literature collection was not feasible since each author used different techniques to collect the relevant articles. However, the articles used by all authors from the different countries indicate review period from 1953 to 2025. A total of 650 articles were initially included in the review but after screening using the eligibility criteria in Fig. 1, they reduced to 565. Authors used their scientific judgment to incorporate the most relevant studies. As methodologies varied considerably across individual studies, developing a fully standardized schematic framework was challenging. However, the general methodology adopted in this study is presented in Fig. 1.

## Sources and mobilization of fluoride

All types of rocks, such as sedimentary, metamorphic, and igneous, as well as non-crystalline materials (e.g., volcanic glass in ignimbrites and tuffs) naturally contain fluorine (De Melo et al., 2020). The maximum fluorine content in these rocks varies widely: igneous and volcanic rocks range from 100 mg/kg (ultramafic) to > 1000 mg/kg (alkaline); sedimentary rocks range from 200 mg/kg in limestones to up to 1000 mg/kg in shales; while metamorphic rocks contain 100 mg/kg to < 5000 mg/kg (Frencken, 1992). Crystalline rocks are generally reported to have higher fluorine concentrations (Nordstrom & Smedley, 2022).

Geogenic fluoride contamination is mostly associated with two major fluorine-bearing minerals: fluorite (CaF_2_) (~ 48 wt% F¯) and fluorapatite (~ 3.8 wt% F¯) (Banerjee, 2015, Garcia and Borgnino, 2015; Schafer, 2020). Fluorapatite (FAP: Ca_10_(PO_4_)_6_F_2_) and the associated variety, carbonate-rich fluorapatite (CFA: Ca_10_(PO_4_)_5_(CO_3_,F)F_2_), are the most widespread fluorine-bearing minerals occurring ubiquitously as a trace component in all rock types; sedimentary, metamorphic, and igneous (basic and acidic) (Filippelli, 2002; Hughes, 2015; Hughes & Rakovan, 2015; Ruttenberg, 2003). Fluorite is much less widespread than FAP and occurs mainly in hydrothermal vein deposits, some acid igneous rocks and rarely as a secondary cement in carbonate rocks (Edmunds & Smedley, 2013, Garcia and Borgnino, 2015, Mukherjee & Singh, 2018). Fluoride also substitutes in amphibole minerals (e.g., hornblende) and phyllosilicates (e.g., biotite). This is because it has a similar ionic radius to the hydroxyl group and can be released when these minerals weather (Edmunds & Smedley, 2013). Other fluoride-bearing minerals, e.g., topaz, Al_2_(SiO_4_)F_2_ (~ 11.5 wt% F¯) are either thermodynamically very stable or, like cryolite, Na_3_AlF_6_ (~ 54 wt% F¯), are very rare in nature due to their high solubility (Garcia and Borgnino, 2015).

The conditions that favour elevated fluoride in groundwater include:

(a) Geogenic sources: The majority of fluoride in groundwater originates from geological sources. Interaction between rocks and groundwater causes fluorite, fluorapatite, and other fluorine-bearing minerals, to weather and release fluoride.

(b) Chemical properties of groundwater: Typically, fluoride dissolution is favoured under specific conditions such as electrical conductivity (EC) between 1000 and 2000 µS/cm, pH between 7.5 and 8.5, and HCO₃/Ca ratios ranging from 0.8 to 2.3 (Saxena & Ahmed, 2003).

(c) Fluorite dissolution processes: The solubility of fluorite (CaF_2_) can be enhanced by processes such as calcite or gypsum precipitation or sodium/calcium exchange, which remove calcium from the solution. This results in elevated fluoride in equilibrium with fluorite according to the law of mass action (Eq. 1) when both calcite and fluorite equilibria control the water chemistry, the coupled reaction is described by Eq. 2 (Nordstrom & Smedley, 2022).

1 $$Ca^{2 + } \left( {aq} \right) + 2F^{ - } \left( {aq} \right) \rightleftharpoons CaF_{2} \left( s \right)$$

2 $$CaCO_{3} + 2F^{ - } + H^{ + } \rightleftharpoons CaF_{2} + HCO_{3}^{ - }$$

(d) Dissolution processes for fluorine-bearing calcium apatite minerals: Similar to fluorite dissolution, the solubility of fluorine-bearing calcium apatite minerals, such as fluorapatite [Ca_10_(PO_4_)_6_F_2_] and carbonate-rich fluorapatite [Ca_10_(PO_4_)_5_(CO_3_)F_2_], can also be enhanced by processes such as sodium and calcium exchange or precipitation of calcium-bearing minerals such as gypsum or calcite, which remove calcium from the solution. The dissolution of calcium apatite is complex, being initially incongruent and pH dependent. Fluorine-bearing apatite minerals (FAP and CFA) rapidly develop a fluoride and calcium depleted surface layer at the water–rock interface that controls the kinetics of further mineral dissolution (Chaïrat et al., 2007a, 2007b; Schafer et al., 2018). Equation 3 nominally represents the initial rapid surface hydrogen exchange reaction for FAP at circum-neutral pH, which generates a depleted surface layer of di-calcium phosphate composition which controls the rate of bulk FAP dissolution (Eq. 4).

3 $$\equiv Ca_{10} (PO_{4} )_{6} F_{2} + 6H^{ + } \left( {aq} \right) \rightleftharpoons \equiv {\mathrm{Ca}}_{6} {\mathrm{H}}_{6} ({\mathrm{PO}}_{4} )_{6} + 4Ca^{2 + } \left( {aq} \right) + 2F^{ - } \left( {aq} \right)$$

where ≡ represents the FAP surface layer

4 $$Ca_{10} (PO_{4} )_{6} F_{2} \left( s \right) \rightleftharpoons 10Ca^{2 + } \left( {aq} \right){ } + 6{\mathrm{PO}}_{4} ^{3 - } \left( {aq} \right){ } + { }2\left( {{\mathrm{F}}^{ - } } \right) \left( {aq} \right)$$

(e) Dissolution of silicate minerals**:** Silicate hydrolysis can also contribute to fluoride enrichment; Eq. 5 and 6 demonstrate the release of fluoride via hydroxyl-fluoride exchange in phlogopite and muscovite, respectively. Finally, Eq. 7 illustrates the oxidative weathering of fluorine-bearing biotite, which releases fluoride alongside potassium and magnesium ions (Ali, 2022; Chae et al., 2007).

5 $$\mathop {{\mathrm{KMg}}_{{3}} \left( {{\mathrm{AlSi}}_{{3}} {\mathrm{O}}_{{{1}0}} } \right){\mathrm{F}}_{{2}} + {\text{ 2OH}}^{ - } }\limits_{{{\mathrm{Phlogopite}}}} \to {\text{ KMg}}_{{3}} \left( {{\mathrm{AlSi}}_{{3}} {\mathrm{O}}_{{{1}0}} } \right) \, \left( {{\mathrm{OH}}} \right)_{{2}} + {\text{ 2F}}^{ - }$$

6 $$\mathop {{\mathrm{KAl}}_{{2}} \left( {{\mathrm{AlSi}}_{{3}} {\mathrm{O}}_{{{1}0}} } \right){\text{ F}}_{{2}} + {\mathrm{2OH}}^{ - } }\limits_{{{\mathrm{Muscovite}}}} \to {\mathrm{KAl}}_{{2}} \left( {{\mathrm{AlSi}}_{{3}} {\mathrm{O}}_{{{1}0}} } \right) \, \left( {{\mathrm{OH}}} \right)_{{2}} + {\mathrm{2F}}^{ - }$$

7 $$\begin{aligned}\mathop 4\mathrm{K}(\mathrm{Mg}_{2}\mathrm{Fe})(\mathrm{AlSi}_{3}\mathrm{O}_{10})(\mathrm{F}, \mathrm{OH})+3\mathrm{O}_{2}+ & 16\mathrm{CO}_{2}+44\mathrm{H}_{2}\mathrm{O} \to \mathrm{Al}_{2}\mathrm{Si}_{2}\mathrm{O}_{5}(\mathrm{OH})_{4}+4\mathrm{K}^{+}+8\mathrm{Mg}^{2+} \\& +4\mathrm{Fe}(\mathrm{OH})_{3}+12\mathrm{H}_{4}\mathrm{SiO}_{4}+16\mathrm{HCO}_{3}^{-}+4\mathrm{F}^{-}\end{aligned}$$

(f) Anthropogenic sources: Coal combustion and use of agricultural fertilizers are two notable anthropogenic contributors to fluoride in the environment. A summary of fluoride concentrations analysed in different fertilizers is provided in Supp. Table 1.

While the highest fluoride concentrations found in groundwater are normally associated with fluorite (CaF_2_) dissolution, globally, naturally elevated fluoride levels can occur even when fluorite is not present in the rock matrix, as long as calcium concentrations are low enough to prevent precipitation of fluoride. This is typically characterized by a strong negative correlation between fluoride and calcium ions, alongside a positive correlation of fluoride with sodium, pH, and bicarbonate ions (Nordstrom & Smedley, 2022; Podgorski & Berg, 2022). While hydroxyl exchange in mica minerals such as biotite can release fluoride, this mechanism alone does not fully explain high fluoride concentrations under these conditions, as it tends to lower pH. Instead, elevated fluoride in groundwater is best explained by the complex dissolution mechanism of ubiquitous trace fluoride-bearing calcium apatite minerals (fluorapatite and carbonate-rich fluorapatite), which requires further research. Key controlling factors for fluoride enrichment include pH, temperature, water residence time, concentrations of key species (calcium and phosphate), and aquifer mineralogy. Thus, geogenic sources are considered to be the primary origin of most instances of fluoride enrichment.

## Fluoride as a global groundwater contaminant

**Fig. 2:**
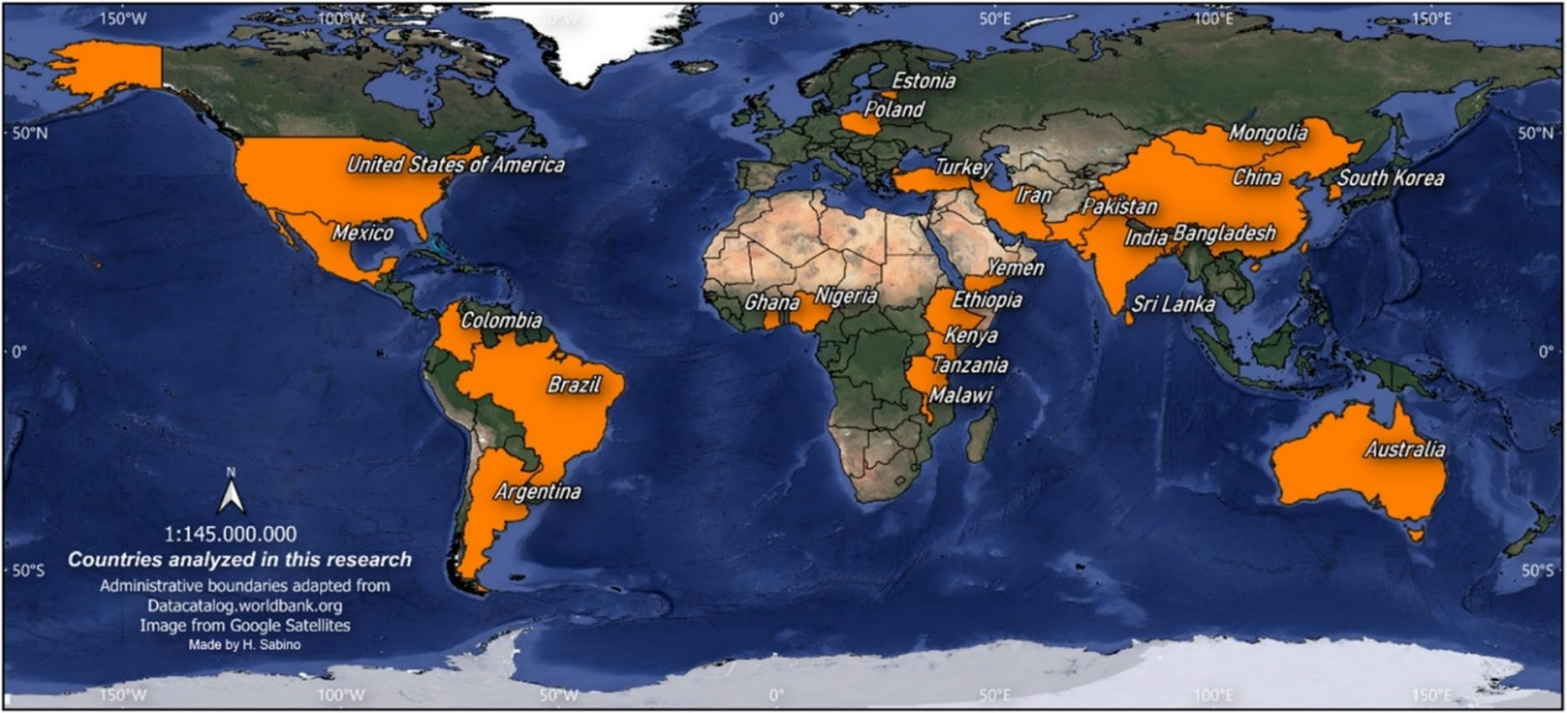
Geographic distribution of the countries evaluated for groundwater fluoride contamination

The study documents fluoride contamination in countries Asia, Africa, Europe, Middle East, America, and Australia with Asia, Africa, Middle East, Europe, the Americas, and Australia, and Australia (Fig. 2). The countries are Argentina, Australia, Bangladesh, Brazil, China, Colombia, Estonia, Ethiopia, Ghana, India, Iran, Kenya, Malawi, Mongolia, Mexico, Nigeria, Pakistan, Poland, South Korea, Sri Lanka, Tanzania, Türkiye, the USA, and Yemen. All countries are extensively investigated and documented in the following sections.

### Asia

In Asia, fluoride contamination is reported for Bangladesh, India, Pakistan, Sri Lanka, China, Mongolia, and South Korea. These countries are documented in the following sections.

#### Bangladesh

**Fig. 3:**
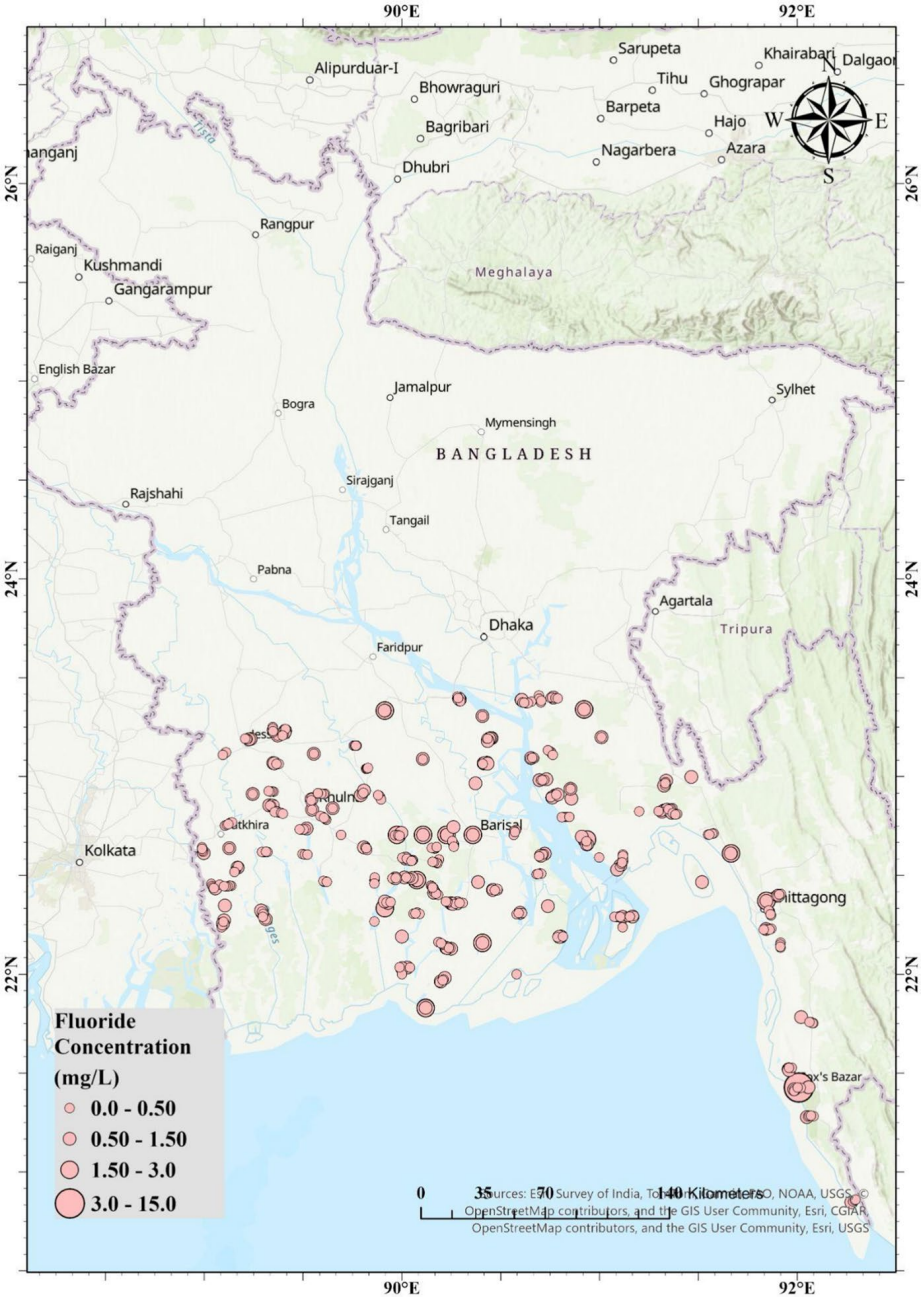
Spatial-distribution of fluoride in the coastal areas of Bangladesh in 2020 (after Rahman et al., 2020) (N = 546)

Bangladesh is mostly located in the Bengal Delta, distinguished by its complex hydrogeological environment and river system. Southeast Bangladesh's coastal areas may be home to an endemic fluorosis outbreak. In Southeast Bangladesh, fluorosis was initially discovered in the early 2000s following an upsurge in discoloured teeth, a common symptom of the disease (Hoque et al., 2003). Consumption of groundwater with a high fluoride content, associated with higher intake of drinking water, was identified as the major source of fluoride. The distribution of fluoride concentrations revealed a distinct pattern in coastal drinking water wells (Rahman et al., 2020; Fig. 3), with fluoride concentrations varying significantly across the country and particularly concentrated in coastal areas (Jannat et al., 2022). Wells drilled into sandstone aquifers, such as those in the southeast coastal region of Bangladesh, contain higher fluoride levels. Low-elevation coastal areas are typical of this region. Groundwater fluoride levels in the bordering Indian states of West Bengal and Assam were relatively higher than those observed in Bangladesh (Narsimha and Rajitha, 2018). Moreover, Hoque et al. (2003) noted the likelihood of high fluoride levels in groundwater of Bangladesh could be due to the border region's similar geological formation. Hoque et al. (2003) reported high fluoride in groundwater in many districts of Bangladesh, including Barisal, Faridpur, Jessore, Khulna, and Rajshahi.

Supp. Figure S1 shows that aquifer-aquitard sediments' depth and thickness patterns vary considerably across several sampling locations in the south-central and eastern coastal plains. This change in aquifer composition leads to spatial and temporal variation in the groundwater fluoride levels. Aquifer sediments are predominantly composed of fine sand and silt, water flows slowly through these porous, fine-grained sediments. The extended residence time of water in these aquifers is likely responsible for the dissolution of fluorine-bearing minerals. Saline water intrusion from the Bay of Bengal and salt-rich rains near the coast may also affect groundwater's chemistry. Shallow groundwater is often contaminated by chloride and fluoride from agro-fertilizer in agricultural areas and saltwater intrusion in coastal belts (Bohlke 2002). Salinity and fluoride in coastal shallow and major aquifers in the country varies widely. Aquifer lithology, hydraulics, and groundwater extraction stressors affect fluoride mobilization in groundwater.

Elevated levels of fluoride in groundwater have been reported in different localities of Bangladesh, with its spatial variability influenced by rainfall patterns, land use, aquifer type, and mineralogy. These parameters regulate water types that vary both vertically and horizontally. Aquifer sediments in the coastal delta of Bangladesh are mostly composed of fine sand and silt admixed with clay, which accelerates the mobilization of fluoride into groundwater through prolonged water–sediment interaction.

Rahman et al. (2020) investigated 19 coastal districts during both wet and dry seasons, highlighting variable fluoride distribution, with the middle and southern areas are reported to have elevated fluoride levels (see Fig. 3 and Table 3). The coastal areas in Barisal and Cox’s Bazar had highest level of fluoride. The high fluoride in the region might be caused by hydrogeological variables, such as the nature of aquifers and the types of groundwater. For example, Na-HCO_3_ water type is often found to be enriched in high fluoride levels (Edmunds and Smedley, 2005).

During the dry season, excessive fluoride levels were observed in the coastal regions of Chandpur, Shariatpur, Pirojpur, Bhola, Jhalokathi, Chittagong, and Cox's Bazar with values of 1.05, 1.30, 1.14, 1.14, 1.37, 1.42, and 1.41 mg L^−1^, which is greater than the recommended threshold value of 1.0 mg L^−1^ (BGD-DPHE, 2001). The low Ca^2+^ and high Na^+^ levels in the groundwater of research areas may also influence the high fluoride concentration. Furthermore, fluoride levels in the dry-season were greater than wet-season levels in terms of geographic extent. Fluoride levels were found to be particularly elevated in Cox’s Bazar Sadar Upazila, Chittagong Sadar, Sandwip, and Patuakhli Galachipa regions.

Both fluoride deficit and excess in drinking water have affected coastal residents. While consumption of groundwater with fluoride below the recommendation level might cause dental cavities (particularly in the demineralization and remineralization of enamel and dentine), prolonged consumption of high fluoride can cause skeletal fluorosis.

Rahman et al. (2020) revealed that coastal Bangladeshi infants are more sensitive to non-carcinogenic health risks due to consumption of fluoride. Hence, clinical, and epidemiological research on fluoride issues is necessary. Rahman et al. (2020) demonstrated the necessity for a major research program to evaluate clinical evidence and therapeutic alternatives. Such initiatives will raise awareness in population living along the coastal areas of Bangladesh, who are unintentionally drinking fluoride contaminated groundwater.

Bangladesh’s southeast coast is the fluorosis hotspot. Therefore, local government and non-governmental groups should focus on developing a low-fluoride water supply initiative to mitigate fluorosis. Inexpensive fluoride content tests and an increased understanding of water cleanliness and health will reduce health concerns in the country.

#### India

India is among the countries that frequently report high fluoride levels in groundwater. With increased institutional focus on providing safe drinking water and groundwater serving as a primary source, numerous studies have investigated the current fluoride situation in the country (Ali et al., 2019, 2023; Jacks et al., 2005; Mukherjee & Singh, 2018).

**Fig. 4:**
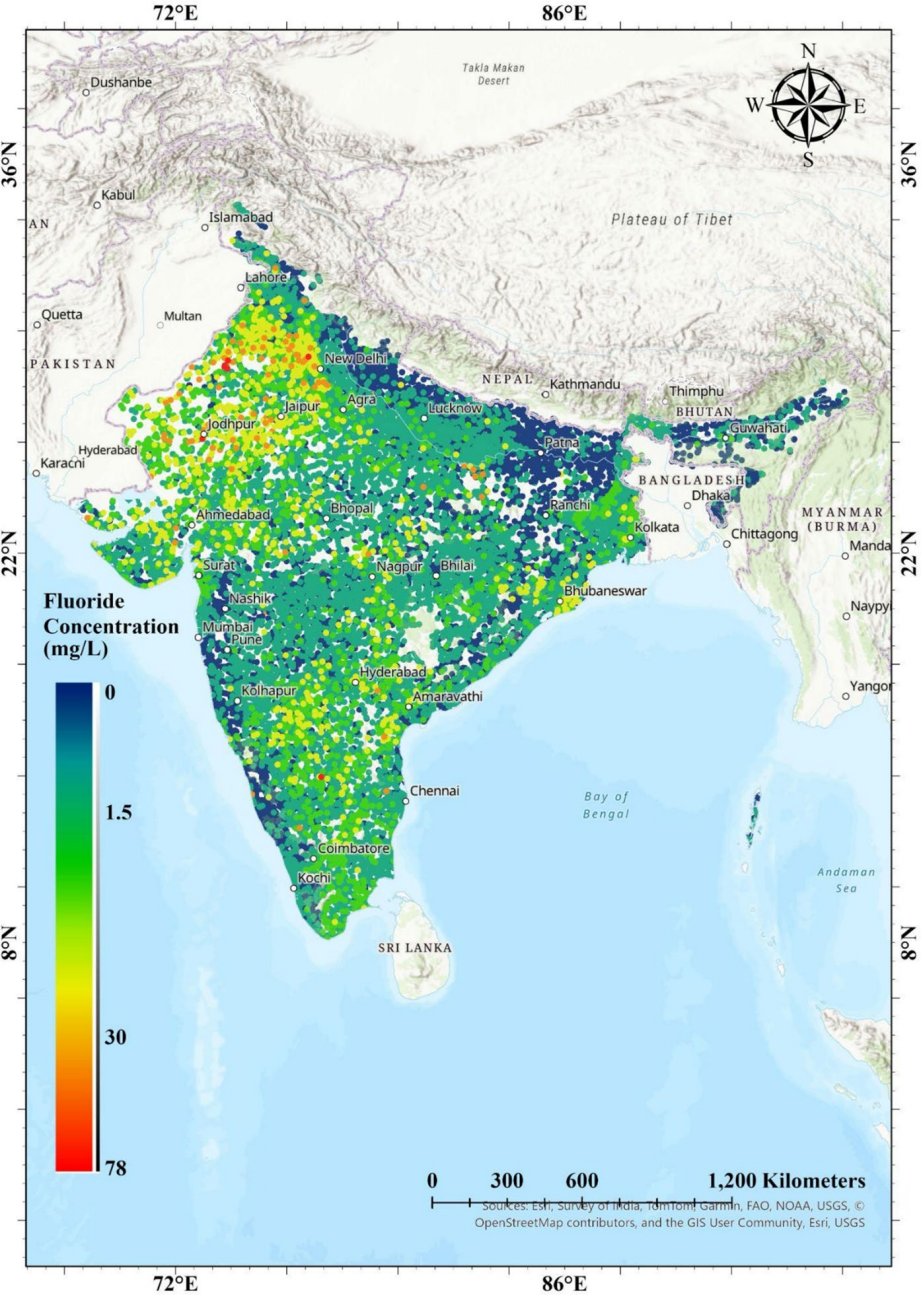
Fluoride concentration in groundwater of India (Central Ground Water Board (CGWB), 2000–2020; N = 127,020; https://cgwb.gov.in/). High concentrations occur mainly in arid and semi-arid regions. The boundaries, names, and designations shown on this map are for academic purposes only and may not reflect officially recognized borders. No endorsement is implied, and the author remains neutral regarding boundary disputes

Hydrogeologically, India comprises 14 principal aquifers. Alluvium is the major aquifer, followed by banded gneissic complex and gneiss aquifers. Other aquifers include sandstone, limestone, basalt, and shale, though these are less widespread than the alluvium or gneissic aquifers (CGWB: Central Ground Water Board; https://cgwb.gov.in/). Smaller areas are covered by schist, granite, quartzite, charnockite, khondalite, laterites, and intrusive aquifers. Fluoride has been reported in almost all aquifer types. However, alluvial aquifers of the Indo-Gangetic plains in the north and the hard rock aquifers of southern India reported to have the highest concentrations of fluoride (Fig. 4).

In India, high fluoride in groundwater is often associated with shallow aquifers, arid to semi-arid conditions with high evaporation rates, alkaline pH, and active ion-exchange processes, which promote leaching of fluoride from fluorine-bearing minerals. Several reviews have summarized fluoride occurrence in Indian groundwater (Ali et al., 2016, 2023; Brindha & Elango, 2011, and references therein). For instance, Mukherjee and Singh (2018) compiled fewer than 100 studies and reported maximum fluoride concentrations of 86.0 mg L^−1^ in northern India (Garg et al., 2009). The most affected states identified were Andhra Pradesh, Tamil Nadu, and Telangana in the south, Rajasthan and Gujarat in the west, and West Bengal in the east. A meta-analysis of about 57,000 samples from 63 studies over the last 35 years indicated an average fluoride concentration of 2.37 mg L^−1^ (range: 1.46–3.28 mg L^−1^, N = 57,381; Ali et al., 2019).

Crippling skeletal fluorosis occurs when individuals are exposed to high fluoride intake (10–20 mg/day) over prolonged periods (10–20 years; NRC 1993). However, cases have also been recorded in areas with average fluoride concentrations of 0.5, 0.7, and 2.8 mg L^−1^, leading to dental, skeletal, and crippling skeletal fluorosis, respectively (Ayoob & Gupta, 2006). Poor nutrition, especially low calcium and vitamin C, further exacerbates the risk of fluorosis.

While geogenic sources are the primary contributors, anthropogenic factors such as coal ash, brick industries, and agricultural fertilizers also noticeably elevate fluoride concentrations in the groundwater of India (Brindha & Elango, 2011). Geographically, higher fluoride concentrations are observed in alluvial plains compared to hard rock regions (Ali et al., 2023). Monitoring by CGWB with over 100,000 datasets (from 2000–2020) confirms that high fluoride is mainly reported from arid and semi-arid areas (see Table 3 and Fig. 4).

**Table 3:** Global overview of groundwater fluoride (F^−^) occurrence across selected countries and regions, showing climatic and hydrogeological settings, dominant aquifer lithologies, statistical distribution of fluoride concentrations (sample size *N*, range, median, and interquartile range), proportion of samples exceeding the WHO guideline value of 1.5 mg L^-1^, suspected geogenic sources, associated co-contaminants, and key literature references (BDL: Below detection limit; IQR: Interquartile range; TDS: Total dissolved solids; EC: Electrical conductivity; NA: Not available; ND: Not detected). Please note that the number of samples (N) presented in the tables may differ from those used for figure generation in the manuscript, due to differences in the underlying datasets (compiled with inputs from all authors)

S. No	Country / Region	Climate / Setting	Dominant aquifers / Geology	N; F^−^ range / median / IQR (mg/L)	% > 1.5 mg/L	Suspected Geogenic Sources	Co-contaminants	Key references
1	Bangladesh – Coastal belt (19 districts)	Tropical monsoon; tidal & estuarine influence	Holocene–Pleistocene deltaic alluvium; Na–HCO₃ water types	N = 840; 0.01–16.11; median ≈ 0.6; IQR ≈ 0.2–1.1	3.9–7.3% (seasonal)	Fluorite/fluorapatite dissolution; ion exchange (Ca ↔ Na); salinity intrusion	Salinity, Na⁺, Cl⁻, As	Rahman et al., 2020
	Bangladesh-Barind Tract / NW Bangladesh (Rajshahi–Naogaon)	Semi-arid, low recharge	Pleistocene terrace deposits; oxidized sands	N ≈ 50–150; 0.2–3.5; median ≈ 1.0; IQR ≈ 0.6–1.8	10–20%	Fluorite-bearing minerals; long residence time	As, Fe, Mn	Hoque et al., 2003
	Bangladesh-Sylhet Basin (NE Bangladesh)	High rainfall; hilly catchments	Alluvial–colluvial sediments; shallow aquifers	N ≈ 20–60; 0.05–1.2; median ≈ 0.4	Rare	Limited fluorite; strong dilution	Fe	Ahmed et al., 2019
	Bangladesh-South-central delta (Faridpur–Gopalganj)	Floodplain, monsoon	Deltaic alluvium	N ≈ 25–70; 0.1–2.0; median ≈ 0.7	~ 5–8%	Apatite/fluorite dissolution	As, Mn	Rahman et al., 2020
	Bangladesh-SE coastal fringe (Chattogram–Cox’s Bazar)	Coastal monsoon; saline intrusion	Deltaic & marine sediments	N ≈ 100 + ; up to 15–16; median ≈ 1.0	> 10% locally	Na-rich waters enhancing F⁻ mobility	Salinity, Na⁺	Jannat et al., 2022
**2**	India (national wide), South Asia	Arid–semi-arid to humid/ alluvial plains and hard rocks	Alluvium (Indo-Gangetic), hard rock (gneiss, granite, basalt), Older alluvium, sandy plains, charnockite	N = 127,020; BDL-78; median = 0.4; IQR = 0.17–0.8	7.9%	Fluorapatite, biotite, fluorite; ion exchange; alkaline pH; Micas in alluvium; long residence time; High-F granites (> 810 mg/kg F)	NO₃⁻, As (mainly in east and eastern India)	www.cgwb.gov.in; Ali et al., 2016, 2019, 2023; Mukherjee & Singh, 2018; Das et al., 2018; Brindha et al., 2023
**3**	Pakistan (South Asia)	Arid- Semi Arid Alluvial plains and hard rock	Alluvial (Unconsolidated and mix) Hard rock (igneous, sedimentary and sedimentary)	N = 21,427; BDL-44.4	11%	Fluorite, fluoroapatite, Biotite, muscovite, granite, silicate weathering	NO_3_ and As (In Punjab and Sindh Province)	Farooqi et al., 2007; Brahman et al., 2013; Rasool et al., 2015 Masood et al., 2022 Iqbal et al., 2023; Kumar et al., 2022b
**4**	Sri Lanka	Dry Zone	Hard rock/Saprolite	N = 6607 Range: 0.10–12.0 Average: 1.20 Median: 0.90	26.9%	High grade metamorphic rocks	Ca and Mg	Chandrajith et al., (2012, 2020)
		Intermediate Zone	Hard rock/Saprolite	N = 871 Range: 0.05–1.17 Average: 0.46 Median: 0.40	0%	High grade metamorphic rocks	Ca and Mg	Chandrajith et al., (2012, 2020)
		Wet Zone	Hard rock/Saprolite	N = 406 Range: 0.02–1.10 Average: 0.49 Median: 0.50	0%	High grade metamorphic rocks	-	Chandrajith et al., (2012, 2020)
**5**	China	Arid and semi-arid basins in northern, north-eastern, north-western China; Loess Plateau and Qinghai-Tibet Plateau; climate influences shallow, deep, and geothermal groundwater	Shallow, deep, and geothermal aquifers; coastal plains (Bohai, Yellow Sea), alluvial basins (Yuncheng, Tarim, Junggar), Guanzhong and Taiyuan basins; geology includes fluorine-rich minerals in sedimentary and volcanic rocks, carbonate formations, and faulted basins	NA	NA	Fluoride from mineral dissolution, water–rock interaction, cation exchange, weathering of fluorine-rich rocks; anthropogenic input from agriculture/industry	Na/Ca imbalance, Fe/Al oxides in some regions	He et al., 2020a, 2020b; Kumar et al., 2020; Fuge, 2019; Li et al., 2011, 2024; Wen et al., 2013; Wu et al., 2015; Deng et al., 2009; Wang et al., 2019, 2023a, 2023b; Hao et al., 2022; Han et al., 2021; Liu et al., 2023; Zhao et al., 2023; Sun et al., 2015; Huang et al., 2023; Xu et al., 2019
**6**	Mongolia	Semi-arid	Depending on the region, there are different types of aquifers with free surface in the river valleys, and with free and pressurized surface aquifers depending on the depth in the Gobi	N = 1030; Range- BDL-7.2; median0.94	27%	Fluorine-bearing minerals, Local mineralization and bedrock geology, hydrogeological conditions, hydrogeochemical conditions,	Mongolia has big country so it depends on the area or regions. Mainly Na, TDS, Cl, As, U, and so on	NIL
**7**	Republic of Korea / East Asia	Temperate / Monsoonal climate	Deep fractured rock aquifer / Precambrian metamorphic rocks and Mesozoic granitoids	N = 355; Range- BDL-40.8; median-5.7; IQR-6.9	75.8%	Major rock-forming minerals such as micas	–	Chae et al., 2007
**8**	Nigeria	Tropical (North central basement-volcanic)	Precambrian crystalline basement with Younger Granite intrusions; fractured granite, gneiss, volcanic rocks; thick weathered saprolite	N = 18; Range- 0.01–10.30	9 of 18 cases (50%)	Fluorite (CaF₂), fluorapatite (Ca₅(PO₄)₃F), biotite, topaz, volcanic glass; prolonged water–rock interaction	Various Major ions, TDS, pH, EC, metal(loid)s, radionuclides, etc	Dibal et al., 2012; Goyit et al., 2018; Lar & Gusikit, 2015; Hyeladi et al. 2014; Akpata et al., 2009
		Tropical (Northeast sedimentary-basement)	Cretaceous-Tertiary Chad Basin sediments; sandstone-clay sequences; localized basement outcrops	N = 13; Range- 0.05–5.00	5 of 13 cases (38.46%)	Fluorite and fluorapatite in clay-rich horizons; evaporative concentration; basement rock weathering	Various: Major ions, TDS, pH, EC, metal(loid)s, radionuclides, etc	Giwa et al., 2021; Oteze and Ayegbusi 2002; Thompson 1965; Dibal and Lar 2005
		Tropical (Northwest basement)	Precambrian basement complex; fractured metamorphic rocks; moderate saprolite development	N = 9; Range- 0.10–3.16	1 of 9 cases (11.11%)	Biotite, fluorite in basement rocks; shorter residence times than Northcentral	Various: Major ions, TDS, pH, EC, metal(loid)s, radionuclides, etc	Aminu and Amadi 2014; Akpata et al., 2009
		Tropical (Southern sedimentary: Southwest, Southeast, South-south)	Cretaceous-Recent coastal sediments; quartz-rich sandstones; kaolinitic clays; high permeability aquifers; basement rocks in the Southwest zone	N = 33; Range- 0.00–6.38*	5 of 33 cases (15.15%)	Trace fluorite and fluorapatite in clay horizons; diagenetic fluoride; localized basement exposure	Various major ions, TDS, pH, EC, metal(loid)s, radionuclides, etc	Emenike et al., 2018; Egbinola and Amanambu 2014; Ibe et al. 1999; Ogbu et al., 2012
**9**	Ghana, West Africa	Tropical (humid south) to semi-arid (north)	Crystalline basement (Birimian, granitoids); Voltaian Basin sedimentary rocks (sandstone, mudstone, limestone)	Range- 0.01–19.5	~ 13–27% in northern districts; ~ 15% national high-risk zones	Weathering of fluorite, fluorapatite, biotite, muscovite, amphiboles; ion exchange; evapotranspiration	Arsenic and nitrate	Sunkari et al., (2018, 2023a and 2023b, 2024, 2025a and b); Ganyaglo et al. (2019); Sunkari and Abu (2019); Zango et al., (2019, 2021, 2025); Atipoka et al. (2020); Abu et al. (2025); Dorleku et al. (2025)
**10**	Ethiopia	Hot and arid lowlands to cool, temperate highlands (10–40 °C)	Volcanic aquifers	N= 1438; Range- < 1.0 to 264.2	42.1%	Rift valley system	Na^+^, As, U, Fe and Mn	Tekle-Haimanot et al., 2006; Rango et al., 2012
**11**	Kenya	Semi-arid to sub-humid regions; volcanic Rift Valley	Volcanic aquifers of the East African Rift Valley (alkaline volcanic rocks, hydrothermal systems)	N = 70; Range- BDL-72; IQR = 0.95–5.74	58.5%	Weathering of fluoride-bearing volcanic and metamorphic minerals, volcanic emissions, hydrothermal water mixing	NA	Gevera et al., 2020 and 2022a and 2022b
**12**	Northern Tanzania, East Africa	East African Rift Valley and Volcanic areas	Tertiary Quaternary unconsolidated sediments, Tertiary Quaternary volcanic rocks, Precambrian Mobile/Orogenic Belt dominated by highly weathered metamorphic rocks, and Precambrian Craton dominated by highly weathered granitic rocks	N = 507, Range- 0.01–74.00; median-1.10; IQR- 2.90	42%	Titanites, Amphiboles, Hornblendes, and Biotites	NA	Ijumulana et al., 2020, 2022; 2024
**13**	Malawi	Hot springs and general groundwater areas; high temperatures contribute to elevated fluoride; emphasizes need for regular monitoring	Groundwater influenced by existing rocks, especially augen gneiss; bauxite, kaolinite, and gibbsite minerals present; rocks are primary geogenic source of fluoride	N = 40; Range- 0.29–3.75; IQR = 0.78–2.31	43.5%	NA	NA	Addison et al., 2020a, 2020b; Msonda et al., 2007; Sajidu et al., 2008; Addison, 2021; Andreah et al., 2023
**14**	Yemen	Semi-arid to arid conditions	Crystalline basement rocks/ sedimentary and volcanic rocks	NA	NA	NA	NA	Al-Amry, 2009; Aqeel et al., 2017; Kadir & Al-Maqtari, 2010; Salman, 2006; Viswanatham, 2008; UNICEF, 2008; Aizari et al., 2021; Al-Amry et al., 2020; NWRA/Taiz, 2006, 2008
**15**	Iran	Semi-ari d to arid	Alluvium, hard rock (Basalt and metamorphic rocks)	N = 1039, Range < 0.02–9.2 Mean: 0.51	28.3%	Volcanic and metamorphic rocks, Cold and thermal springs, fluorine- bearing clay minerals, shales, Ore deposits (Kiruna-type Fe oxide/apatite deposits, Pb–Zn, coal)	Nitrate, Arsenic, High salinity	Dehbandi et al. (2018), Balaghi Enalou et al (2018), Yousefi et al. (2019a), Asghari Moghaddam and Fijani (2008)
**16**	Türkiye, Europe	Dry summers and mild, rainy winters (Mediterranean climate) and old winters and hot/dry summers (continental climate)	Volcanic, carbonate	N = 209, range 0.1–57, average: 3.41	52%	Felsic volcanic rock, e.g. rhyolite	Boron, Arsenic	MTA, 2005
**17**	Estonia, Europe	Estonia lies in the northern part of the temperate climate zone and in the transition zone between maritime and continental climates. Estonia is situated in the north–western part of the East European Platform	The crystalline Paleoproterozoic basement is overlain by Neoproterozoic (Ediacaran) and Palaeozoic (Cambrian, Ordovician, Silurian, and Devonian) sedimentary rocks and covered by Quaternary deposits. Estonian sedimentary rocks form five aquifer systems—Middle Devonian, Devonian-Silurian, Silurian–Ordovician, Ordovician–Cambrian and Cambrian–Vendian	Devonian; N= 70, range 0.04- 2.0, median 0.49, IQR 0.50; Devonian-Silurian; N= 205, range 0.10-3.48, median 1.30, IQR 0.90; Silurian-Ordovician; N= 297, range 0.01-7.20, median 1.54, IQR 2.07; Ordovician-Cambrian; N= 117, range 0.10-3.0, median 1.10, IQR 0.60; Cambrian-Vendian; N= 107, range 0.04-1.88, median 0.60, IQR 0.34	Devonian 5.7%; Devonian-Silurian 35.6%; Silurian-Ordovician 50.8%; Ordovician-Cambrian 17.1%; Cambrian-Vendian 3.7%	Clayey carbonate rocks, K-bentonites	Boron	Uppin & Karro, 2013; Uppin & Karro, 2012; Haamer & Karro, 2006; Karro et al., 2009
**18**	Poland Opole Province, Paczków-Niemodlin Basin, Europe	Cold without dry seasons, avg. Temp. 8.7 °C, rainfall 639 mm	Neogene, Miocene, sands,	N = 43, range- 0.05–11.5; median- 1.51	60%	Long-term contact with Precambrian and Palaeozoic igneous and metamorphic rocks and their weathering products containing minerals rich with fluorine	Sometimes with high pH	Razowska-Jaworek L., Cudak J. (2022)
**19**	Mexico, North Central Plateau, North America	Semiarid to arid	Alluvial	N = 14,058 (covering all Mexico), range 0.03—27.9, median 3.5	40%	Felsic volcanic rock, e.g. rhyolite	Arsenic	Alarcón-Herrera et al., 2020
	Mexico, hydrothermal waters of the Transvolcanic Belt, North America	Temperate	Alluvial, fractured rock	N = 88, range 0.15—12	42%	Felsic and intermediate volcanic rocks	Arsenic	Hurtado & Gardea-Torresdey, 2004; Armienta & Segovia, 2008
	Mexico, Yucatan Peninsula, North America	humid	Carbonate	NA	NA	NA	NA	Alarcón-Herrera et al., 2020
**20**	Argentina (National Wide) South-America	Arid–semi-arid to humid. Alluvial fans in Andean Mountain environment, to large fluvial plains	Volcanic basaltic and andesitic rocks, granitoids at the mountain area. Loess like and fine sand Quaternary sediments at the humid plains area	N = 739, range 0.013–12.7; median 1.78	60%	Hydrothermal fluids, glass volcanic shards in sedimentary environment	Arsenic	Nicolli et al., 1989; Blarasin et al., 2011
**21**	Brazil, South America	Tropical	Paleomesozoic intracratonic sedimentary basins and fractured aquifers of the Precambrian crystalline basement	N = 112,849, range- BDL- > 1.444	1.3% above 1.444 mg L^-1^	Dissolution and hydrolysis of accessory minerals (fluorite, apatite, micas) in crystalline and sedimentary matrices and long residence time	Phosphate fertilizers and industrial effluents	Venturini et al. (2016), Lima et al. (2019), Fraga (1992), and Paulino et al. (2022)
**22**	Colombia / South America	Tropical; Andean, Pacific, Caribbean and inter-Andean regions; high geological and tectonic variability	Surface water–dominated supply with localized groundwater influence. Sedimentary rocks (sandstones, shales, marls) and volcanic rocks (andesites, basalts); tectonically active zones (e.g., Neiva fault)	N = 149 (Cauca region); range: 0.012–0.210; Other studies: 0.04–0.07 (Montería aqueduct); < 0.83 (Cauca rivers near Puracé volcano); median/IQR not reported	NA	Weathering of sedimentary and volcanic rocks; tectonic activity facilitating fluoride release; localized groundwater interaction	-	Misnaza-Castrillón et al., 2021; López-Salgado et al., 2016; Revelo-Mejía et al., 2021; Revelo-Mejía et al., 2022
**23**	United state Of America (Country wide), North America	Western USA: arid to semi-arid, low flow, limited flushing; Central-eastern USA: humid; California: hydrogeology and climate influence fluoride	25 national aquifers; Ordovician carbonate rocks (central-east); crystalline rocks (generally low fluoride, higher in west); shallow and deep aquifers, deeper wells show higher fluoride due to water–rock interaction	N = 38,105; ND-160; median = 0.2; IQR = 0.1–0.5	5.98%	Fluoride from mineral dissolution	NA	McMahon et al., 2020; Edmunds & Smedley, 2013; Zhang et al., 2017; Belitz et al., 2022; Chaudhuri & Ale, 2014; Reedy & Scanlon, 2020; Harkness & Jurgens, 2022; Rosecrans et al., 2022
**24**	Australia / Great Artesian Basin	Arid and semi-arid	Cadna-owie/Hooray Aquifer/ sedimentary -continental sandstone / fluvial and marine mudstone and siltstone	N = 4883 Range: 0.9 to 19 Median: 1.5 IQR 2.9	42%	High fluoride concentrations in the artesian groundwater have been attributed to groundwater being in contact with igneous (hydrogeological basement) rocks or with fine-grained sedimentary rocks. A likely primary mineralogical source is ubiquitous trace fluorapatite in igneous rocks or trace carbonate-rich fluorapatite in fine-grained sedimentary rocks. Primary fluoride may also be derived from mica group minerals such as biotite	NA	Ransley et al. (2015); Schafer (2020)

^*^The 6.38 mg L^−1^ maximum in Ibadan (Egbinola and Amanambu 2014) represents localized basement exposure and potential anthropogenic influence; typical sedimentary values < 1 mg L^−1^. Hydrogeologic characterisation of fluoride occurrence across Nigeria's contrasting geological provinces was based on compilation of studies in Supp. Table S4. *N* represents number of individual research reports (cases) per region. Overall range is the minimum and maximum values reported across all studies in a region. Percentage of studies reporting > 1.6 mg L^−1^ indicates proportion exceeding guideline limit. Co-contaminants represent parameters commonly reported alongside fluoride in the water systems within each province

In northern India, older alluviums and sandy plains, particularly in Delhi and Haryana, are largely affected (Ahada & Suthar, 2019; Ali et al., 2016, 2021; Ali & Ahmad, 2025; Duggal & Sharma, 2022; Gagandeep Singh et al., 2020). Micas in older alluviums are found to be a significant geogenic source in the groundwater of Delhi (Ali, 2022). Shallow unconfined aquifers (7–30 m deep) comprising alluvium, sand, and kankars (kankar is the local term for calcareous nodules) in Haryana report maximum fluoride up to 86.0 mg L^−1^ (min = 0.14 mg/L, N = 275; Garg et al., 2009). Punjab and Haryana, two major users of chemical fertilizers, also experience co-occurrence of fluoride with nitrate, as reported by Iqbal et al. (2023) in Punjab, Pakistan (fluoride from 0.06 to 7.9 mg L^−1^ and nitrate from 0.1 to 70.0 mg L^−1^, N = 161).

Central India, dominantly covered by Deccan basalt hard rocks, exhibits higher fluoride in deeper aquifers due to prolonged water–rock interaction (min = 0.06 mg L^−1^, max = 12.71 mg L^−1^, N = 101) (Pandith et al., 2016). Diverse rock formations including granite, gneiss, limestones, sandstones, and basalts contribute to fluoride enrichment. Hydrothermal fluorite mineralization in Proterozoic meta-sedimentary successions is a dominant source in limestones and overlying basalts, whereas fluorapatite (2.5–4.2 wt% F¯) is the primary fluoride source in granite gneiss aquifers (min = 0.23 mg L^−1^, max = 4.13 mg L^−1^, N = 57) (Dubey et al., 2021).

In western India, Gujarat’s mainland regions with alluvial deposits are more affected with fluoride than Saurashtra and Kutch, which comprise Deccan traps, limestone, sandstone, and recent alluvium (min = 0.1, max = 9.6 mg L^−1^, N = 6407) (Senthilkumar et al., 2021). In north Gujarat, fluoride is mainly derived from granitoid-granulite suite rocks (min = 0.17 mg L^−1^, max = 2.7 mg L^−1^, N = 43) (Pradhan & Biswal, 2018). Geothermal water in southern Gujarat shows fluoride levels of 2.3 – 8.0 mg L^−1^ due to the interaction with fluorine-bearing minerals (N = 3) (Shah et al., 2019). Rajasthan reports elevated fluoride in several localities (min = 0.1 mg L^−1^, max = 34.0 mg L^−1^) (Choubisa et al. 2023).

In eastern India, high fluoride in groundwater has been linked to biotite and fluorapatite in gneissic rocks, with numerous affected localities in Jharkhand (Patel et al., 2014; Srikanth et al., 2008; Thapa et al., 2019) and Odisha (Maitra et al., 2021; Sahu et al., 2020). Geothermal processes also contribute to fluoride in Odisha (Maitra et al., 2021; Paikaray & Mahajan, 2023). West Bengal reports co-occurrence of fluoride with arsenic, particularly in shallow aquifers (24–30.5 m), and found to decrease with depth (> 152 m). Raniganj sandstones, a primary aquifer in West Bengal, contain 248 mg/kg total fluoride (De et al., 2022; Gupta et al., 2012).

The northeastern states of India such as Assam, Meghalaya, Nagaland, Manipur, Mizoram, Arunachal Pradesh, Tripura, and Sikkim (see Supp Figure S2), have limited hydrogeological studies. Large geographic belts with elevated fluoride concentrations are associated with granitic, gneissic, volcanic rocks, and sediments of marine origin, which characterize the region’s geology (Singh et al., 2018). Several studies have documented high fluoride levels in drinking wells installed on the Brahmaputra alluvial plains (Bhattacharya et al., 2020; Das et al., 2003; Gogoi et al., 2021; Sharma et al., 2012; see Supp. Table S2).

The mechanisms controlling fluoride release in this region vary with redox conditions. In reducing environments, fluoride mobilization is predominantly driven by reductive dissolution, whereas in oxidizing environments, higher pH generally enhances fluoride release (Das et al., 2018; Kumar et al., 2016, 2020). Groundwater fluoride concentrations in Assam have been reported as high as 9.0 mg L^−1^ (Gogoi et al., 2021), 14.7 mg L^−1^ (Sahadevan & Chandrasekharam, 2008), and 17.13 mg L^−1^ (Hanse et al., 2019). Major fluorine-bearing minerals in the region include fluorite, biotite, and muscovite, derived from the weathering of hard rock aquifers. Fluorine content in granite, micaceous quartzite, and phyllite from Assam ranges from 670–1200, 270–340, and 578 mg/kg, respectively (Sahadevan & Chandrasekharam, 2008).

Southern India is dominated by crystalline hard rock aquifers (Saha et al., 2024; Subramaniyan et al., 2022), and groundwater in these aquifers frequently exhibits high fluoride concentrations. States such as Andhra Pradesh, Telangana, Karnataka, and Tamil Nadu report the largest number of districts endemic to high fluoride at the national level (https://cgwb.gov.in/). Fluorine-bearing minerals in these hard rocks commonly exceed the global average of 810 mg/kg (Wedepohl, 1969), making them significant sources of fluoride to groundwater through rock-water interactions. For example, granites from Hyderabad contain ~ 910 mg/kg fluoride (mean content; Ramamohana Rao et al., 1993).

Apart from natural sources, anthropogenic sources also contribute to elevated fluoride levels in groundwater. Brindha and Elango (2013) analysed fertilizers from southern India and reported maximum fluoride levels of 13 mg/kg. In Dharmapuri district, Tamil Nadu, charnockites and gneisses contain an average of 35.8 mg/kg and 59.4 mg/kg fluoride, respectively (Jagadeshan et al., 2015). A recent analysis of 13,585 groundwater samples from ~ 2,300 monitoring locations across Tamil Nadu reported fluoride concentrations from 0.01 to 5.0 mg L^−1^ (Brindha et al., 2023). This study also highlighted a critical limitation of national monitoring networks; high-fluoride areas are often underrepresented due to sparse and unevenly distributed observation wells. However, establishing high-density networks remains a major challenge in both developing and developed world.

#### Pakistan

Pakistan is characterised by an arid to semi-arid country with mix topography consisting of high snow peaks to low-lying coastal areas and complex geology (Acidic pluvial to unconsolidated deposits) (Supp. Figure S3). Northern, Western, and Southern part comprises mostly rigid mountains and the Eastern part comprises the Indus plain. Variable fluoride concentrations in the groundwater have been reported across different districts and sub-districts of Khyber Pakhtunkhwa (KPK), Sindh, Balochistan, Punjab, and Gilgit Baltistan (Farooqi et al., 2007; Pakistan Council of Research in Water Resources (PCRWR) 2017; Rashid et al., 2018). Both natural and anthropogenic sources contribute to fluoride in groundwater. Weathering of fluorine-bearing minerals is the primary driver of elevated fluoride concentrations across all provinces (Durrani & Farooqi, 2021; Muhammad & Ullah, 2022; Naseem et al., 2010), while anthropogenic sources have been reported in Lahore, Kasur, and Kalalan Wala districts of Punjab (Farooqi et al., 2009).

High fluoride content has been more frequently documented in finer clay aquifers compared to sandy aquifers (Durrani & Farooqi, 2021; Khattak et al., 2022). However, metamorphic and igneous rocks dominate as the main geological controls, contributing to the prevalence of fluoride in the groundwater (Supp. Table S3). In KPK, groundwater fluoride ranges from below detection limit (BDL) to 6.7 mg L^−1^. High groundwater fluoride has been reported in 12 districts, with the highest concentrations reported in Haripur, underlain by metamorphic rocks (Mehmood et al., 2021) (see Supp. Figure S3). In Sindh, fluoride level ranges from 0.06 to 44.4 mg L^−1^. Highest values are reported in Umarkot, followed by Mithi, Nagarparkar, Badin, and Karachi. Elevated groundwater fluoride in Sindh is largely attributed to the weathering of fluorine-bearing minerals in igneous rocks (Brahman et al., 2013; Rafique et al., 2008, 2009, 2015; Siddique et al., 2006; Talpur et al., 2020).

**Fig. 5:**
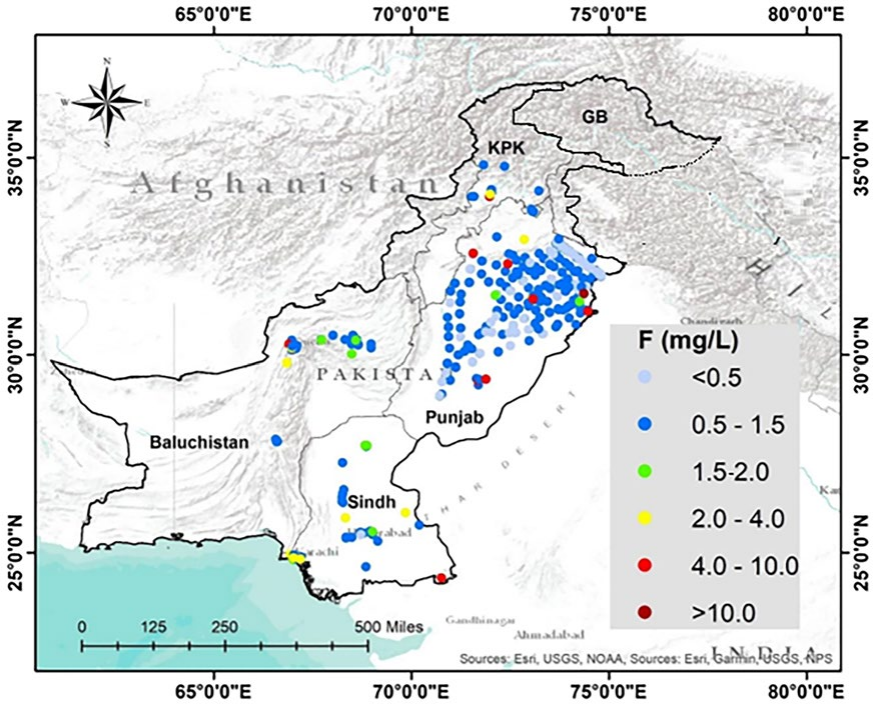
Fluoride concentration in groundwater of Pakistan (N = 21,427). Disputed boundaries are indicated by dotted lines based on United Nations cartographic conventions. The boundaries, names, and designations shown on this map are for academic purposes only and do not imply official endorsement or acceptance by the author or publisher. The author remains neutral with regard to jurisdictional claims and boundary disputes

In Balochistan, groundwater fluoride ranges from BDL-24.0 mg L^−1^, highest fluoride content has been reported in groundwater of Quetta, followed by Loralai, Sibi, and Mastung (see Fig. 5 and Supp. Figure S3) (Chandio et al., 2015; Durrani & Farooqi, 2021; Tahir & Rasheed, 2013). Although Balochistan being the least investigated province, over 7 districts are affected by high fluoride. The lithology of Quetta and Sibi includes unconsolidated to mixed carbonate sedimentary rocks while Mastung, Pringabad, Gwadar, and Mangochar are dominated by igneous rocks. Loralai has a mixed geology; sedimentary, metamorphic, and igneous rocks potentially contributing to fluoride in the groundwater. In Ziarat, despite the presence of igneous rocks, high fluoride has not been reported, which could be due to lower temperatures and higher seasonal precipitation.

In Punjab, groundwater fluoride ranges from BDL-21.0 mg L^−1^, with over 20 districts being affected with the highest fluoride levels reported from Lahore (Khattak et al., 2022). Punjab is dominated by unconsolidated sediments, evaporites in the centre, while igneous and carbonate sediments in the northwest. However, anthropogenic activities have contributed to elevated fluoride levels, with maximum values reaching up to 21.0 mg L^−1^ (Farooqi et al., 2009). Gilgit Baltistan, in northern Pakistan, generally has low fluoride concentration in the groundwater, which could be attributed to high precipitation and low temperatures, although elevated fluoride levels have been observed in only one district (Diamer) due to complex lithology and domination of igneous rocks (https://www.pcrwr.gov.pk/).

In an estimate, approximately 13 million people in Pakistan are affected by fluoride-contaminated drinking water (Ling et al., 2022). Health impacts of consuming groundwater with high fluoride are evidenced in Sibi, where chronic exposure has been linked to dental enamel abnormalities, bone deformities, joint pain, and crippling fluorosis (Chandio et al., 2021). Saeed et al. (2021) reported elevated oxidative stress and lower IQ levels among school children in fluoride-exposed areas, while Bibi et al. (2023) documented neuronal and non-neuronal fluoride toxicity affecting the cholinergic system. However, further research is required to fully understand these health impacts.

In summary, densely populated districts such as Lahore, Karachi, Sargodha, Depalpur, Peshawar, Bannu, and Quetta are particularly vulnerable to fluorosis due to high population density. Most of the population lacks knowledge about fluoride contaminated drinking water and its health implications. Mass level public awareness and installation of efficient water filters at public distribution points is needed to minimize the harmful effect of fluoride on public health.

A total number of 21,427 samples were used for developing groundwater fluoride maps of Pakistan (Table 3 and Fig. 5). Groundwater fluoride concentrations ranged from BDL-44.4 mg L^−1^. Nearly, 11% of the samples exceeded permissible limit of 1.5 mg L^−1^. Undesirable groundwater fluoride limits have been observed in studies conducted in the early twenty-first century (Khan et al., 2002; Smedley, 2001). Furthermore, the nationwide survey revealed that the groundwater fluoride levels surpass both lower and upper limit of WHO and National Drinking Water Quality Standards (NDWQS) in many localities of Pakistan (https://www.pcrwr.gov.pk/).

The hydrochemical conditions controlling groundwater fluoride are alkaline conditions, high dissolved solids, Na-HCO_3_ to Na-Cl water type caused by weathering, dissolution, and ion exchange of silicate minerals in the aquifer media. The fresh weathering, dissolution or recharge in the aquifers are represented by Na-HCO_3_ type water followed by the Na-Cl type water that indicates the precipitation of calcite minerals and the enhancement of fluoride, due to the influence of the evaporation process (Farooqi et al., 2007, 2009; Chandio et al., 2021; Masood et al., 2022; Durrani et al., 2021, 2025).

#### Sri Lanka

Sri Lanka is a tropical island at the southern tip of the Indian Peninsula, with a total land area of approximately 65,000 km^2^. A semi-arid climate prevails over two-thirds of the island, except for the high mountains and the southwestern terrain. Different geomorphological features and two major climatic domains largely govern the distribution of fluoride. Based on rainfall patterns, Sri Lanka can be divided into two main climatic regions: the wet zone (> 2500 mm/year) and the dry zone (< 1000 mm/year), with an intermediate zone lying between them.

Groundwater is the primary source of drinking water, especially for rural communities in the dry zone (Chandrajith et al., 2020). In the dry zone, groundwater occurs in shallow saprolite or deep fracture zones within high-grade metamorphic rocks, except in the northern and northwestern coastal belts, where sedimentary limestone predominates. Limited rainfall and prolonged dry periods create water scarcity, leading communities to rely on shallow dug wells (< 10 m) and deep tube wells that tap water-bearing weaker zones in crystalline rocks. The dry and intermediate climate zones are known for high-fluoride groundwater (Chandrajith et al., 2012, 2020; Dissanayake, 1996), which has significantly affected water quality and public health. As a result, a high prevalence of dental fluorosis and, in some cases, skeletal fluorosis has been reported. Additionally, rising incidences of chronic kidney disease of uncertain etiology are suspected to be linked to high fluoride in groundwater (Balasooriya et al., 2020; Chandrajith et al., 2011; Liyanage et al., 2022). Estimates suggest that in some areas, 80–90% of the population suffers from dental fluorosis (Nunn et al., 1994; Warnakulasuriya et al., 1992).

**Fig. 6:**
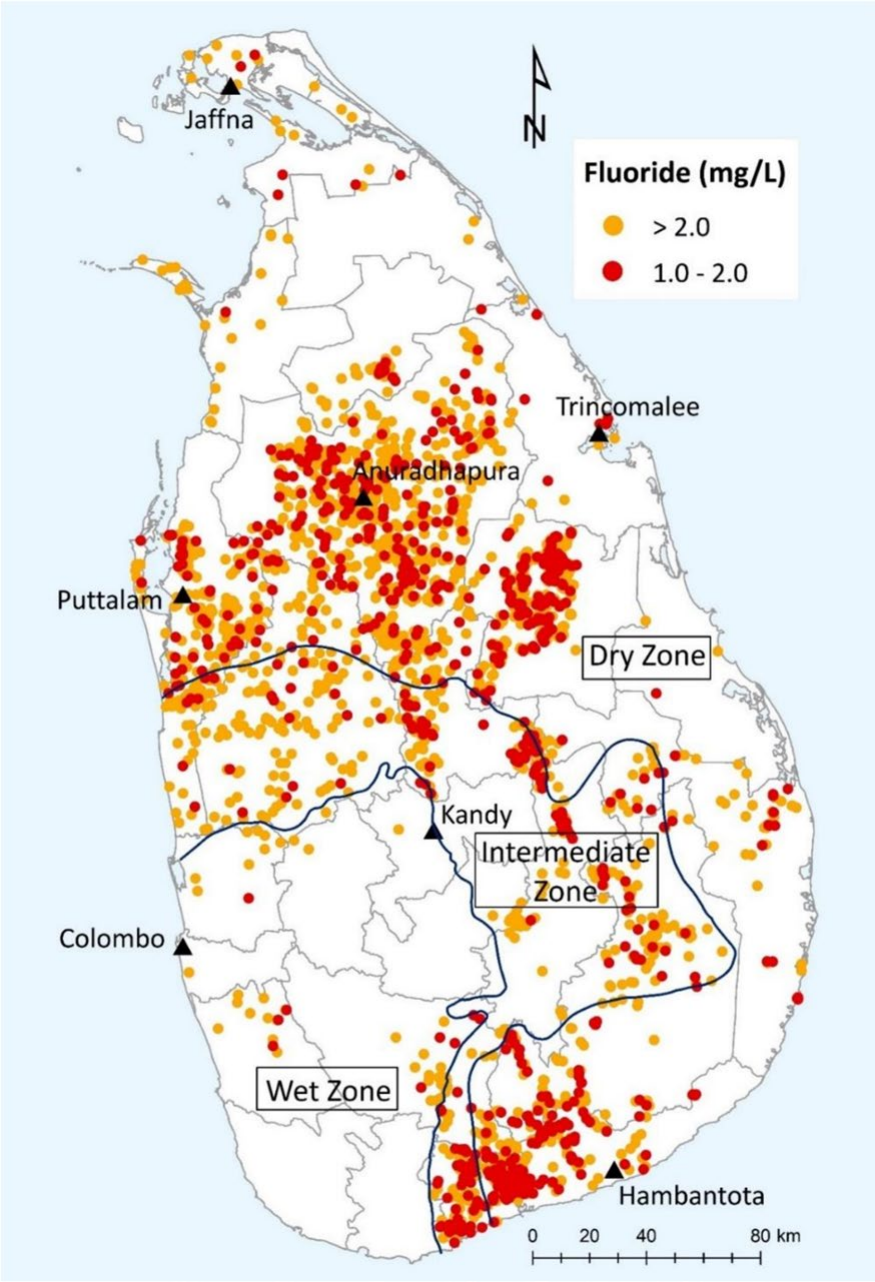
Distribution of high fluoride (> 1.0 mg/L) wells in Sri Lanka with respect to climatic boundaries (N = 1598); 26% groundwater samples are ≥ 1.5 mg/L of fluoride

Over the past two decades, substantial studies have examined groundwater in the dry zone. However, the wet zone remains under-investigated (Fig. 6). Data from nearly 6,107 wells in Sri Lanka indicate fluoride levels ranging from < 0.02 to 12.0 mg L^−1^, with 28% samples (N = 1710) below 0.5 mg L^−1^ and 9.7% (N = 592) above 2.0 mg L^−1^ (see Table 3; Ranasinghe et al., 2019). In dry-zone metamorphic terrain, over 50% of wells contained fluoride above 0.6 mg L^−1^, while 19% (N = 1181) had fluoride above 1.5 mg L^−1^ and 2.3% (N = 142) showed levels above 4.0 mg L^−1^ (Chandrajith et al., 2020). The highest average recorded in Monaragala District (1.4 ± 1.3 mg L^−1^) of Sri Lanka (Xu et al., 2021). In contrast, none of the wells in the wet zone showed a fluoride level above 1.5 mg L^−1^. Dry zone sedimentary aquifers in the north and northwest have lower fluoride levels, with the highest mean of 0.55 mg L^−1^ in Murunkan limestone aquifers and 0.35 mg L^−1^ in northern Jaffna (Chandrajith et al., 2016; Thilakerathne et al., 2015). Similarly, in the wet zone, the highest reported mean fluoride content is 0.55 mg L^−1^, with a mean of 0.46 mg L^−1^.

High-fluoride groundwater is generally associated with elevated dissolved mineral content (Rubasinghe et al., 2015; Xu et al., 2021). Although rocks and minerals in the wet zone are similar to those in the dry zone, the reported fluoride levels are much lower (Dissanayake, 1996). Thus, climate and hydrological conditions strongly influence the occurrence and distribution of fluoride. Low rainfall and high evaporation in the dry zone enhance fluoride concentrations, whereas high rainfall in the wet zone promotes chemical weathering and leaching of fluoride from soils and rocks.

As a result, a high prevalence of dental fluorosis and, in some cases, skeletal fluorosis has been reported. Additionally, rising incidences of chronic kidney disease of uncertain etiology are suspected to be linked to high fluoride in groundwater (Balasooriya et al., 2020; Chandrajith et al., 2011; Liyanage et al., 2022). Estimates suggest that in some areas, 80–90% of the population suffers from dental fluorosis (Nunn et al., 1994; Warnakulasuriya et al., 1992).

Fluoride content in Sri Lanka’s metamorphic rocks ranges from 95 to 1440 mg/kg (Dharmagunawardhane & Dissanayake, 1993). Groundwater extracted from charnockites gneiss, calc-gneiss, biotite gneiss, and granulite generally has higher fluoride than that from quartzite and crystalline limestone aquifers. Deep groundwater from the fractured crystalline rocks has longer residence times, allowing extended water–rock interaction and greater dissolution of fluorine-bearing minerals. In contrast, wet-zone aquifers experience greater fluoride leaching due to higher rainfall and weathering (Dissanayake, 1996). Even within the same aquifer type, fluoride content differs drastically between dry and wet zones (Chandrajith et al., 2020). Pronounced spatial heterogeneity in groundwater fluoride concentrations is evident even over very short distances. For example, wells exhibiting elevated fluoride concentrations (> 2.0 mg L^−1^) were observed within approximately 500 m of wells containing low fluoride levels (< 0.5 mg L^−1^) (Ranasinghe et al., 2019). The analysis further showed that 26% (N = 1598) of dentally unsafe groundwater sources (wells with ≥ 1.5 mg L^−1^ F¯) had access to an alternative low-fluoride source (< 1.0 mg L^−1^) within a 500 m radius. In addition, 39% of high-fluoride wells (≥ 2.0 mg L^−1^) were located within 500 m of a well with fluoride concentrations below 1.5 mg L^−1^ when the WHO guideline value of 1.5 mg L^−1^ for optimal fluoride levels was applied. These findings highlight strong small-scale spatial variability in fluoride distribution and suggest that localized mitigation through well switching may be feasible in certain settings.

#### China

Groundwater is an essential source of drinking water for millions of people in China. However, in recent years, there is a growing concern regarding elevated fluoride levels in groundwater (He et al., 2020a, 2020b; Kumar et al., 2020). The distribution of groundwater fluoride in China is highly variable, showing wide spatial and temporal variations. It is estimated that over 68 million people in China consume elevated levels of fluoride through drinking groundwater, mainly in the northern, north-eastern, and north-western regions, particularly in arid and semi-arid basins (He et al., 2020a, 2020b; (see Supp. Figure S4). This non-uniform distribution is largely attributed to geological factors, including the presence of fluorine-bearing minerals in surrounding rocks (Fuge, 2019; Li et al., 2024). Elevated fluoride in groundwater can be classified into three types based on occurrence: shallow high-fluoride groundwater, deep high-fluoride groundwater, and high-fluoride geothermal water (He et al., 2020a, 2020b; Wang et al., 2019).

High fluoride in shallow aquifers is commonly observed in north-eastern, north-western, and northern China, as well as in parts of the Chinese Loess Plateau (Wen et al., 2013). Numerous studies have confirmed that the natural occurrence of fluorine-rich minerals in geological formations is a primary factor contributing to elevated fluoride in shallow groundwater, such as in the Taiyuan Basin (Li et al., 2011). Wu et al. (2015) also found elevated fluoride levels in middle and lower confined aquifers in Shizuishan City, Northwest China. The main sources of fluoride include natural weathering of rocks and minerals, as well as anthropogenic activities such as agriculture and industrial discharges. Aquifer leakage has also been identified as a significant factor influencing fluoride concentrations (Wu et al., 2015). Shallow groundwater with fluoride concentrations up to 9.52 mg L^−1^ is widespread in the Guanzhong Basin, North-Western China, primarily due to natural factors such as climate, hydrogeochemical conditions, and geological structures (Deng et al., 2009).

Deep high-fluoride groundwater has been recorded in several regions, particularly in the coastal plains of Bohai and the Yellow Sea, as well as in alluvial plains and basins like Yuncheng, Tarim, and Junggar (He et al., 2020a, 2020b; Wang et al., 2019). In the Shendong mining area, fluoride concentrations were very high, ranging from 0.12 to 13.92 mg L^−1^ in the wet season and 0.20 to 17.58 mg L^−1^ in the dry season, exceeding the Chinese drinking water guideline (1.0 mg L^−1^) by more than tenfold. Hao et al. (2022) observed that anthropogenic activities, precipitation, evaporation, and mineral dissolution are key factors affecting deep groundwater fluoride (see Table 3). High fluoride concentrations in deep groundwater have also been reported in the Datong Basin (Northern China), ranging from 1.5 to 8.69 mg L^−1^, where the solubility of carbonate and fluorine-bearing minerals along with evapotranspiration primarily dominate (Wang et al., 2023a, 2023b). In the North China Plain, fluoride tends to be released more easily under certain geochemical conditions, particularly when the Na/Ca ratio is high and iron and aluminium oxides are present (Han et al., 2021).

High-fluoride geothermal water poses major challenges in several regions (Liu et al., 2023; Wang et al., 2019), particularly in the Qinghai-Tibet Plateau (Zhao et al., 2023). Studies of confined geothermal water in faulted basins of the Qinghai-Tibet Plateau reported fluoride levels up to 5.7 mg L^−1^. Enrichment of fluoride in geothermal water is primarily influenced by natural factors, such as ion exchange, pH, and mineral saturation (Liu et al., 2023). For example, geothermal water in the Yangbajing Geothermal Field, Tibet, contains high fluoride levels ranging from 17.9 to 19.6 mg L^−1^, attributed to a mixture of deep geothermal fluids, leaching of fluoride, and silicate minerals (Sun et al., 2015). Huang et al. (2023) reported that geothermal groundwater in fault zone areas of Southeast China contains fluoride levels nearly ten times higher than the WHO safe limit, primarily due to dissolution of fluorine-bearing minerals, high temperatures, cation exchange, and alkaline conditions. Furthermore, Xu et al. (2019) studied Xi’an geothermal water in the Guanzhong Basin and found maximum fluoride levels up to 14.8 mg L^−1^. This is attributed to geogenic processes including cation exchange, mineral dissolution, and mixing processes.

#### Mongolia

In Mongolia, only limited studies have been conducted on fluoride contamination. The Khangai region in the east is marked by low fluoride concentrations, whereas the Gobi region exhibits higher fluoride levels in groundwater. The territory of Mongolia can generally be divided into regions where groundwater is insufficiently enriched with fluoride and regions where it is sufficiently enriched. Groundwater in the southeastern region is sufficiently enriched in fluoride due to fluorine-rich deposits. Fluorite mineralization from geologic formations in the southeast exhibit a major spatial control on groundwater fluoride concentrations (Baatarkhuu et al., 1991).

**Fig. 7:**
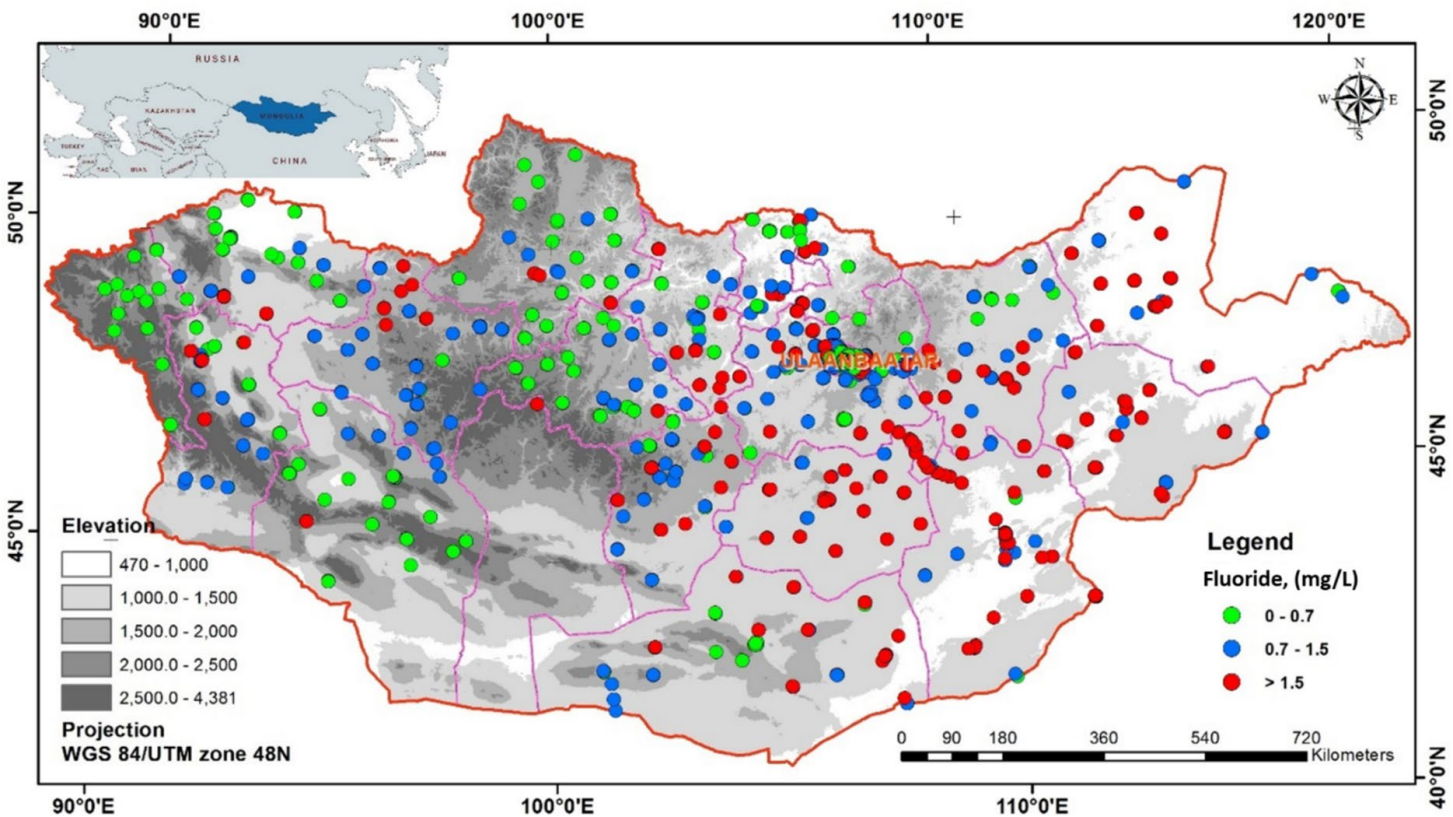
Fluoride distribution in groundwater of Mongolia (N = 1030)

A drinking water quality study including fluoride was carried out between 2019 and 2022, where a total of 1,030 samples were analysed to generate the fluoride distribution map of Mongolia. The median concentration was 0.94 mg L^−1^, with a maximum value of 7.2 mg L^−1^. Concentrations exceeding 1.5 mg L^−1^ were observed in 27% of the samples (see Table 3 and Fig. 7). It was observed that fluoride concentrations in western and central parts of the country were below or within the drinking water standard. However, groundwater in the eastern and Gobi regions had high fluoride concentration (Fig. 7). Elevated fluoride levels are likely related to the local geology, particularly fluorite and phosphorite ore deposits, which are widespread in these regions (Baatarkhuu et al., 1991). Long residence times in aquifers and slow groundwater movement contributed to high fluoride concentrations due to prolonged water–rock interaction (Brunt et al., 2004). The Gobi region’s arid climate, with limited precipitation and almost no recharge to groundwater, further increases fluoride concentrations through evaporation and lack of dilution.

#### South Korea

The South Korean peninsula, located in East Asia between eastern China and Japan, is an ideal region to study the geologic controls of fluoride chemistry due to its complex geology. The geology of the country comprises Precambrian massif rocks, sedimentary rocks of Palaeozoic to Cenozoic ages, and granitic intrusions and volcanic rocks largely of Jurassic and Cretaceous age (Chough et al., 2000).

Fluoride concentration and distribution in South Korean groundwater have been examined in a few studies (Chae et al., 2006a, 2007, 2008; Kim et al., 2014, 2020; Kim & Jeong, 2005; Lee et al., 2022). These studies indicate that fluoride concentrations exceeding the Korean drinking water standard (1.5 mg L^−1^) are frequently encountered in groundwater, limiting its direct use. The average fluoride concentrations in groundwater from granite areas tend to increase with depth: deeper wells (> 300 m) had an average and median fluoride concentration of 4.5 mg L^−1^ and 2.6 mg L^−1^, respectively, whereas shallow wells (100–300 m) averaged only 0.5 mg L^−1^. This suggests that groundwater from deeper granite wells requires treatment before drinking. Multivariate statistical analyses of bottled mineral water from < 200 m deep wells showed that these waters are mostly Ca-HCO_3_ type with low total dissolved solids (TDS), reflecting their shallow-depth circulation to avoid the excess of fluoride levels (Lee et al., 2022).

**Fig. 8:**
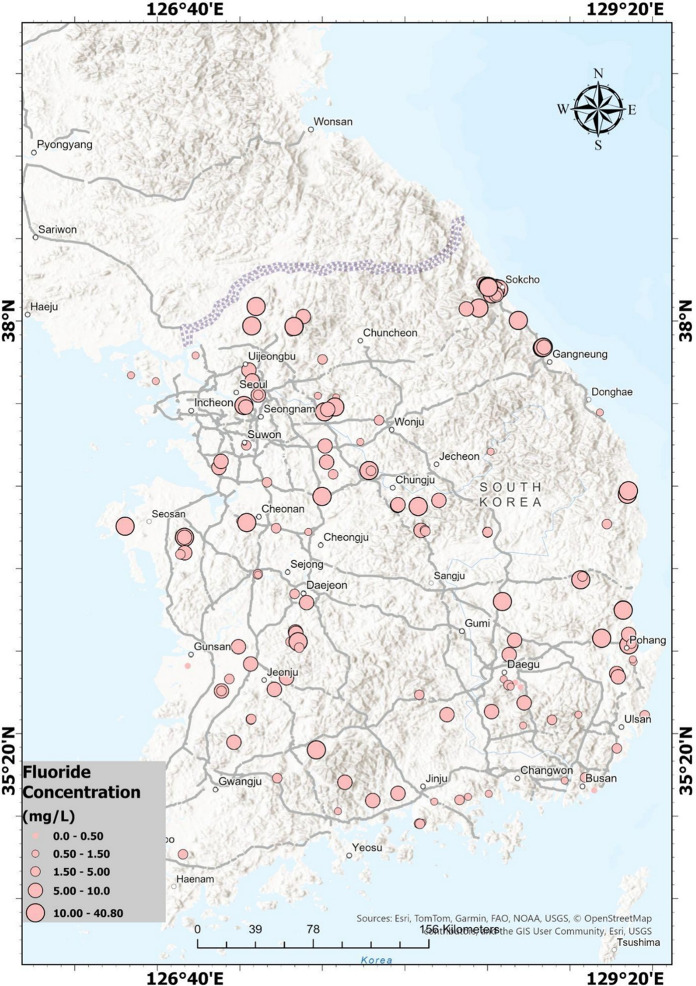
Fluoride distribution in deep thermal groundwater of South Korea (N = 204)

A comprehensive understanding of fluorine geochemistry in deep groundwater was conducted by Chae et al. (2007). Using 355 hydrochemical datasets from deep boreholes (average depth = 624 ± 262 m) in hot spring areas, the study found that: i) mean, median, and maximum fluoride concentrations were very high, reaching 4.4 mg L^−1^, 5.7 mg L^−1^, and 40.8 mg L^−1^, respectively (Table 3 and Fig. 8); ii) fluoride levels were higher in Precambrian metamorphic rocks, mainly granitic gneiss (mean 8.7 mg L^−1^; median 7.1 mg L^−1^), and Mesozoic granitoids (mean 6.0 mg L^−1^; median 5.6 mg L^−1^), compared to sedimentary rocks (mean 3.6 mg L^−1^; median 1.6 mg L^−1^) and volcanic rocks (mean 2.9 mg L^−1^; median 2.1 mg L^−1^). Similar enrichment of fluoride in granite areas was also reported in rural areas of the central part of South Korea (Chae et al., 2008).

Chae et al. (2006b) investigated fluoride enrichment in granite groundwater using batch dissolution experiments with Mesozoic granite and its biotite in pure water for 1200 h. Key findings are: i) high fluoride concentrations (up to 6.0–10.0 mg L^−1^) were leached from granite and biotite; ii) correlations between ions suggested fluoride originates from dissolution of fluorine-bearing biotite; iii) after ~ 500 h, fluoride concentration gradually decreased due to supersaturation with respect to fluorite due to dissolution of Ca^2+^ bearing plagioclase; iv) fluoride leaching from biotite increased gradually until fluorite saturation, influenced by Ca^2+^ removal via adsorption or cation exchange on secondary clay surfaces. The study concluded that biotite in Korean granites is a significant source of fluoride (Chae et al., 2006b).

A hydrogeochemical study in southeastern South Korea showed high fluoride enrichment in groundwater, often along faults that facilitate deep groundwater circulation (Kim & Jeong, 2005). Kim et al. (2014) also observed that fluoride enrichment is characteristic of deeply circulated, alkaline Na-Ca-HCO_3_ type groundwater with prolonged water–rock interaction through granitic rocks. Compared to Na-HCO_3_ type groundwater, Ca-HCO_3_ type groundwater generally exhibits lower fluoride concentrations (Chae et al., 2007; Kim et al., 2020). In Ca-HCO_3_ waters type, increasing TDS and Ca^2+^ is attributed to gradual carbonate and plagioclase dissolution. In contrast, alkaline Na-HCO_3_ type groundwater is enriched in fluoride and Na⁺ due to silicate dissolution and removal of Ca^2+^ by calcite precipitation and cation exchange. In summary, 1) high fluoride enrichments in South Korean fractured bedrock aquifers are frequently encountered in the deep, alkaline Na–HCO_3_ water facies and 2) deep groundwater with higher natural Ca^2+^ contains less fluoride than waters with lower Ca^2+^, likely due to varying degrees of fluorite saturation.

### Africa

In Africa, elevated fluoride levels have been reported in several countries, including Nigeria, Ghana, Ethiopia, Kenya, Tanzania, and Malawi. Among these countries, Ethiopia, Kenya, Tanzania, and Malawi are located near the East African Rift Valley (EARV), which is recognized as a major geogenic source of fluoride contamination in groundwater. The entire East African Rift Valley is known as a high-fluoride region. Elevated concentrations above the WHO safe limit have been reported from Ethiopia in the north (Dagnaw et al., 2017; Gizaw, 1996; Kebede et al., 2016), throughout Uganda (Egor & Birungi, 2020; Robinson et al., 2005; Rwenyonyi et al., 2001), Tanzania in the central region (Bosshard-Stadlin et al., 2017; Memba et al., 2021; Tomašek et al., 2022), and Malawi in the south (Addison et al., 2020c; Msonda et al., 2007). This section provides a detailed examination of fluoride contamination in countries such as Nigeria, Ghana, Ethiopia, Kenya, Tanzania, and Malawi, covering its sources, genesis, and mechanisms of mobilization.

#### Nigeria

Nigeria is a tropical West African country with an area of approximately 923,773 km^2^ and a population of more than 200 million (Ajibola 2000). The country is underlain by two major geological provinces: Northern Nigeria and parts of the southwest are dominated by Precambrian to Jurassic crystalline basement rocks, whereas the southern regions are predominantly covered by Cretaceous to Recent sedimentary sequences (Egbueri et al., 2025). This geological variation creates distinct hydrogeological settings that govern fluoride occurrence, mobilization, and concentration across the six geopolitical zones of Nigeria (see Supp Table S4; Supp. Figure S5). Studies have been conducted on fluoride contamination in Nigerian water resources since 1954 (Wilson, 1954), though research remains unevenly distributed. Supp. Figure S5 reveals that northern zones account for approximately 53% of documented fluoride studies (Northcentral 24%, Northeast 17%, Northwest 12%), whilst southern zones contribute around 47% (Southwest 17%, Southeast 16%, South-south 14%). This geographical bias partly reflects actual contamination patterns, as northern basement terrains exhibit systematically elevated fluoride concentrations compared to southern sedimentary provinces. However, this highlights research gaps in southern Nigeria where fluoride contamination, though generally lower, remains inadequately characterized in many localities.

The elevated fluoride concentrations reported from northern Nigerian groundwater systems is mainly driven by mineralogical composition of Precambrian basement complexes and associated volcanic rocks (Akpata et al., 2009; Dibal et al., 2012; Giwa et al., 2021; Goyit et al., 2018). These crystalline rocks contain abundant fluorine-bearing minerals (Lar & Gusikit, 2015; Uriah et al., 2014). The Jos Plateau volcanic province in Northcentral Nigeria and Langtang area have crystalline rock geology with abundance of fluorine-bearing minerals such that Younger Granite intrusions and associated volcanic rocks host exceptionally high fluoride levels ranging from 0.12 to 10.30 mg L^−1^ (Dibal et al., 2012; Goyit et al., 2018; Hyeladi et al. 2014). Similarly, the Panyam and Kerang volcanic provinces exhibit fluoride concentrations between 0.12 and 0.59 mg L^−1^ in groundwater and springs (Dibal and Lar 2005; Lar & Gusikit, 2015). The release of fluoride from these geological sources is primarily through chemical weathering processes which is further accelerated by the tropical climate conditions of Nigeria. The high temperatures (annual mean 27–32 °C) and substantial rainfall (annual totals 500–3000 mm varying north to south) promote intensive mineral dissolution reactions. Fluorite dissolution proceeding via the equilibrium reaction is the direct source of fluoride in groundwater (see Eq. 1).

However, progressive water–rock interaction leads to increase in the calcium concentrations, which further suppresses fluorite dissolution and causes precipitation of secondary CaF_2_ when saturation is exceeded, thus alter the groundwater chemistry. This dynamic explains why intermediate fluoride concentrations (1.0–3.0 mg L^−1^) are more common than extreme values in Nigeria (see Supp. Table 4 and Table 3), despite abundant fluorite in source rocks. Fluoride is also released through fluorapatite weathering via incongruent dissolution facilitated by acidic conditions generated through CO_2_ dissolution and organic acid production in tropical soils (Eq. 8; Dorozhkin, 2012; Chaïrat et al., 2007b; Tõnsuaadu et al., 2021; Rey et al., 2008; Borgnino et al., 2013):

8 $${\mathrm{Ca}}_{{5}} {\mathrm{(PO}}_{{4}} {)}_{{3}} {\mathrm{F}}_{{\mathrm{(S)}}} + {\mathrm{6H}}_{{\mathrm{(aq)}}}^{ + } \to 5{\mathrm{Ca}}_{{\mathrm{(aq)}}}^{{2 + }} + 3{\mathrm{H}}_{{2}} {\mathrm{PO}}_{{\mathrm{4(aq)}}}^{ - } + {\mathrm{F}}_{{\mathrm{(aq)}}}^{ - }$$

**Table 4:** Country-wise specific applications of various defluoridation technologies, highlighting commonly used methods, their advantages and limitations, operational performance, costs, and region-specific implementation factors. (abbreviations used include AA (Activated Alumina), RO (Reverse Osmosis), ED (Electrodialysis), POU (Point of Use), O&M (Operation and Maintenance), USD (United States Dollar), and F⁻ (fluoride ion)

Country/regions	Common technologies	Advantages	Disadvantages	Costs	Specific factors	Operational Performance (% removal; capacity)	O&M Cost (USD/m^3^)	Waste/Brine Management	Supply-chain & Acceptability
India	Nalgonda, activated alumina, bone char, reverse osmosis, adsorption; Defluoridation	Nalgonda and bone char (low cost and simple), activated alumina (high efficiency and reusable), reverse osmosis (high efficiency)	Nalgonda (labor intensive), activated alumina (cost and requires regeneration), bone char (taste/odor), reverse osmosis (high cost)	Very low cost (Nalgonda) Moderate (activated alumina) Low cost (bone char)	Nalgonda for communities, activated alumina for domestic use, and RO in urban areas (Choubisa, 2023; Smet & Wijk, 2002)	Nalgonda: 70–90% removal (Suneetha et al., 2008); AA: 85–95%, 1.0–1.5 g F⁻/kg (Ghorai & Pant, 2005); Bone char: 80–95%, 1.5–3.5 g F⁻/kg (Ayoob et al., 2008); RO: > 95% (Mohapatra et al., 2009)	Nalgonda: 0.02–0.05 USD/ m^3^; AA: 0.10–0.30 USD/m^3^; Bone char: 0.05–0.15 USD/ m^3^; RO: 0.50–1.50 USD/m^3^ (Yadav et al., 2018)	Nalgonda: Al/F sludge requires lined disposal (NEERI, 1987); AA: NaOH regeneration brine needs neutralization; Bone char: spent media landfilled; RO: 20–30% reject brine (Jagtap et al., 2012)	AA via UNICEF/govt programs; bone char faces cultural resistance in communities; RO limited to urban areas (Meenakshi & Maheshwari, 2006)
Tanzania	Nalgonda, bone char; Defluoridation	Low cost and simple	Nalgonda (labor-intensive) Bone char (taste/odor)	Very low cost (Nalgonda) Low cost (bone char)	Community and household levels (Dahi, 1996; Mbabaye et al., 2017)	Nalgonda: 70–85% removal; Bone char: 85–95%, 0.75–2.0 g F^−^/kg (Kaseva, 2006; Mbabaye et al., 2017)	Nalgonda: 0.01–0.04 USD/ m^3^; Bone char: 0.03–0.10 USD/ m^3^ (Dahi, 2016)	Nalgonda: sludge disposed in pits; Bone char: spent char used as soil amendment (Jacobsen, 2007)	Local bone char production established; Nalgonda chemicals (alum, lime) available; good community acceptance after sensitization (Fawell et al., 2006)
China	Adsorption, precipitation, ion exchange; mixed (Defluoridation/fluoridation)	Large-scale and effective	Sludge formation	Moderate-High	Focus on innovation and stricter fluoride standards (Wang & Reardon, 2001)	Modified AA/rare earth: 90–98%, 5–15 g F⁻/kg; Precipitation: 60–80%; Ion exchange: 95–99% (Bhatnagar et al., 2011; Tang et al., 2009)	Adsorption: 0.15–0.40 USD/ m^3^; Precipitation: 0.10–0.25 USD/ m^3^; Ion exchange: 0.30–0.80 USD/ m^3^ (Yadav et al., 2018)	CaF_2_ sludge for industrial reuse; ion exchange regeneration brine requires treatment (Mohapatra et al., 2009)	Strong domestic manufacturing; government-subsidized programs; stricter 1.0 mg/L standard (WHO, 2017)
Sri Lanka	Calcined clay/brick filters	Very low-cost and locally available materials	Possesses short life with variable efficiency	Very low	Low-cost, locally made, for household use (Padmasiri & Dissanayake, 1995; Smet & Wijk, 2002)	40–70% removal (variable); 5–20 L/d; 0.5–1.5 g F^−^/kg; filter life 3–6 months (Padmasiri & Dissanayake, 1995)	0.01–0.03 USD/ m^3^ (excluding labor) (Ayoob et al., 2008)	Spent clay/brick disposed locally; minimal environmental concern (Smet & Wijk, 2002)	Materials locally abundant; traditional technology; good acceptance but requires frequent replacement (Dissanayake, 1991)
Ethiopia, Kenya, Tanzania, Ghana, South Africa	Bone char, Nalgonda, activated alumina, reverse osmosis; Defluoridation, except limited water fluoridation in Ghana and water fluoridation in South Africa	Nalgonda and bone char (low cost and simple), activated alumina (high efficiency and reusable), reverse osmosis (high efficiency)	Nalgonda (labor intensive), activated alumina (cost and requires regeneration), bone char (taste/odor), reverse osmosis (high cost)	Very low cost (Nalgonda) Moderate (activated alumina) Low cost (bone char) High cost (reverse osmosis)	Depends on local context and resources (Ndé-Tchoupé et al., 2015; Smet & Wijk, 2002)	Bone char: 85–95%, 1.5–3 g F^−^/kg (Korir et al., 2009); Nalgonda: 70–90% (Yami et al., 2018); AA: 85–95%; RO: > 95%; 10–1000 L/d (Fawell et al., 2006)	Bone char: 0.02–0.08 USD/ m^3^; Nalgonda: 0.01–0.05 USD/ m^3^; AA: 0.15–0.35 USD/ m^3^; RO: 0.40–1.20 USD/ m^3^ (Dahi, 2016)	Bone char: agricultural reuse common; Nalgonda sludge: pit disposal; AA: regeneration waste needs treatment; RO: brine disposal challenge in arid areas (Ndé-Tchoupé et al., 2015)	Bone char well-established in Kenya/Tanzania (CDN, Nakuru); cultural issues in Muslim areas; AA/RO limited by cost and supply chains; NGO support critical (Fawell et al., 2006; Jacobsen, 2007)
Estonia, Finland	Monitoring for small-scale treatment	–	–	–	No large-scale artificial defluoridation; focus on monitoring (Seppä et al., 2000 and 2002; Indermitte et al., 2007)	N/A (primarily monitoring); small POU filters where needed: > 90% removal (Seppä et al., 2000 and 2002)	N/A (minimal treatment); POU systems: 0.50–2.00 USD/ m^3^ where used (Indermitte et al., 2007)	Minimal; small-scale cartridge disposal through municipal waste	Low F⁻ prevalence; alternative water sources preferred; commercial POU available but limited demand (Seppä et al., 2000 and 2002)
Venezuela	Salt fluoridation	–	–	–	Switched from water to salt fluoridation (Estupiñán-Day, 2005)	N/A (fluoridation, not defluoridation)	N/A (fluoridation program)	N/A	Salt fluoridation program established; no defluoridation needed due to naturally low F⁻ (WHO, 2017)
Developed countries	Reverse osmosis, electrodialysis, distillation	Very high efficiency	High cost and energy	High cost	Advanced but costly, mainly in urban/wealthier settings (Coerver et al., 2021; Smet & Wijk, 2002)	RO: 95–99%, > 1000 L/d/unit; ED: 85–95%; Distillation: > 99%; industrial scale: 100–10,000 m^3^/d (AWWA, 2011; Mohapatra et al., 2009)	RO: 0.30–1.00 USD/ m^3^; ED: 0.40–1.50 USD/ m^3^; Distillation: 1.00–3.00 USD/ m^3^ (Missimer et al., 2021; Voutchkov, 2018)	RO/ED: 15–25% reject brine, often to sewer or evaporation ponds; membrane replacement every 3–5 years (Panagopoulos et al., 2019)	Commercial systems readily available; regulatory compliance standard; high consumer acceptance; energy costs main constraint (USEPA, 2005)

Under more acidic conditions (i.e., pH < 5), complete protonation may occur (Eq. 9; Chaïrat et al., 2007a, 2007b; Wagman et al., 1982; Tõnsuaadu et al., 2021; Rey et al., 2008; Schafer et al., 2018):

9 $${\mathrm{Ca}}_{{5}} \left( {{\mathrm{PO}}_{{4}} } \right)_{{3}} {\mathrm{F}}_{{\mathrm{(s)}}} + \, 9{\mathrm{H}}^{ + }_{{\mathrm{(aq)}}} \to 5{\mathrm{Ca}}^{2 + }_{{\mathrm{(aq)}}} + 3{\mathrm{H}}_{{3}} {\mathrm{PO}}_{{\mathrm{4(aq)}}} + {\mathrm{F}}^{ - }_{{\mathrm{(aq)}}}$$

The phosphate released through this pathway competes with fluoride for adsorption sites on iron and aluminium oxide surfaces abundant in tropical weathering profiles, potentially enhancing the mobility of fluoride. This mechanism is more relevant in northern Nigerian basement terrains where deep weathering profiles (saprolite thickness often > 30 m) provide extensive reactive surface area for sustained fluorapatite dissolution over prolonged residence times.

The tropical weathering in Nigeria promotes rapid biotite alteration in the upper saprolite zone, creating a continuous fluoride flux to circulating groundwater (Ojanuga, 1973; Tijani et al., 2006; Yunusa et al., 2024). Biotite with significant modal abundance of fluoride (typically 10–25% in the Nigerian basement granitoids) can also act as an important geogenic source (Issa, 2022; Onipe et al., 2020; Babatunde, 2021; Kamaunji et al., 2024; Adabanija et al., 2020).

The northcentral zone, where volcanic CO_2_ and silicate weathering lead to release of naturally alkaline groundwater, fluoride concentrations frequently exceed 2.0 mg/L (Dibal et al., 2012; Goyit et al., 2018). Additionally, the inverse, negative, and weak relationship between calcium and fluoride has been observed consistently in Nigerian groundwater datasets, with high-fluoride waters characteristically exhibiting low calcium despite extensive water–rock interaction (Emenike et al., 2018; Igibah et al., 2022; Iwar et al., 2021).

Nigerian basement aquifers generally have higher concentration of fluoride due to longer residence times of water which leads to progressive dissolution and accumulation of fluorine-bearing minerals (Dibal et al., 2012; Lar & Gusikit, 2015; Uriah et al., 2014). Deep confined aquifers (> 100 m depth) in fractured basement rocks have residence times estimated at decades to centuries, although quantitative age-dating is limited in Nigerian groundwater studies. These systems show systematically higher fluoride (typically > 1.5 mg L^-1^) compared to shallow unconfined aquifers (< 30 m depth) with residence times of months to few years (typically < 1.0 mg/L) despite similar mineralogical compositions (Dibal et al., 2012; Hyeladi et al. 2014). This pattern reflects cumulative mineral dissolution along flow paths, with progressive increase in the fluoride concentrations as the water migrates deeper into the fractured basement systems.

The observation by Dibal et al. (2012) that boreholes and hand-dug wells show similar fluoride concentrations in some northern localities, initially appears contradictory to residence time control. However, this may be due to the localized hydrogeologic conditions where shallow weathered zones (regolith aquifers) have experienced sufficiently prolonged water–rock interaction leading to elevated fluoride levels which are comparable to deeper systems. In tropical basement terrains, the thick saprolite mantle provides extensive reactive surface area where even relatively young groundwater can acquire elevated fluoride through rapid mineral weathering under high temperature conditions. Further, a vertical hydraulic connectivity through fracture networks may permit mixing between shallow and deep groundwater components, homogenizing fluoride concentrations across depth intervals in some settings. Reaction progress indicators (such as silica, total dissolved solids, sodium/calcium ratios) corroborate residence time effects on the evolution of fluoride concentrations. Groundwater samples with high fluoride consistently exhibit elevated silica, total dissolved solids, and sodium/calcium ratios, which is a characteristic of advanced water–rock interaction and hydrogeochemical maturation (Akpata et al., 2009; Lar & Gusikit, 2015). Conversely, low-fluoride samples typically exhibit dilute compositions with lower silica and total dissolved solids, indicating limited water–rock interaction consistent with short residence times and rapid recharge-discharge cycling.

Table 3 depicts the interrelationships between geological setting, aquifer characteristics, and fluoride occurrence across the contrasting hydrogeologic provinces of Nigeria. The northcentral zone exhibits the highest fluoride prevalence (having more cases with > 1.6 mg L^-1^) owing to its distinctive volcanic-basement geology. As stated earlier, the Jos Plateau volcanic province hosts Younger Granite ring complexes and associated volcanic rocks enriched in fluorine-bearing minerals. Aquifers in this region are made up of fractured granite, weathered saprolite, and volcanic rock sequences where groundwater typically circulates at moderate to slow velocities, allowing extensive water–rock interaction. The co-contaminants include nitrate (NO_3_) and soil heavy metal(loids) associated with radioactive granite intrusions through shared alkaline, reducing groundwater chemistry (Lar & Gusikit, 2015). The northeast shows moderate levels of fluoride which is controlled by sedimentary-basement transitions. The Gombe and Maiduguri localities overlie Cretaceous-Tertiary sedimentary basins where groundwater circulates through sandstone aquifers interbedded with fluorine-bearing clay horizons. Fluoride ranges from BDL to 5.0 mg L^-1^ with approximately most of the regions reporting values > 1.6 mg L^-1^ (Table 3). The exceptionally high value of 5.0 mg L^-1^ reported by Oteze and Ayegbusi (2002) for Maiduguri likely reflects stagnant groundwater conditions in semi-confined aquifer zones with prolonged residence times exceeding several decades. High pH (> 5.7), increased conductivity, and high tropical temperatures (> 28 °C) could also lead to high fluoride concentration through evaporative concentration effects in the semi-arid regions (Giwa et al., 2021). The Northwest exhibits lower fluoride despite basement geology similar to northcentral Nigeria. This apparent contradiction reflects differences in mineralogy and groundwater flow regimes. Northwestern basement rocks contain less abundant fluorine-bearing minerals and the aquifer systems in this region experience rapid recharge-discharge cycles driven by seasonal monsoonal rainfall. Consequently, the median fluoride levels have been observed to be lower than northcentral Nigeria (Aminu and Amadi 2014; Akpata et al., 2009). The Southern sedimentary provinces of Nigeria show systematically lower fluoride across all the three zones (Southwest, Southeast, South-south). Fluoride concentrations up to 6.38 mg L^-1^ was reported from southern parts, with extreme values concentrated in the Ibadan region which may be due to localized conditions (Egbinola and Amanambu 2014). Typical southern sedimentary values were < 1.0 mg L^−1^ (see Supp. Table S4). These lower levels of fluoride can be attributed to the aquifer mineralogy which is dominated by quartz-rich sandstones, kaolinitic clays, and minor carbonate cements. Fluoride sources in this region include trace fluorite and fluorapatite in clay-rich horizons and diagenetic fluoride incorporation during sediment lithification (Ibe et al. 1999; Uriah et al., 2014). However, these sources are quantitatively insufficient to release high concentration of fluoride, due to the limited availability of reactive mineral content and generally short groundwater residence times (years to few decades) in highly transmissive sand aquifers. The notable exception occurs in southwestern Ibadan where Egbinola and Amanambu (2014) reported BDL–6.38 mg L^−1^ of fluoride, with the high end-member values attributed to localized basement rock exposure through sedimentary cover along with potential anthropogenic contamination from industrial activities.

Geological settings and conditions are the main drivers of fluoride in water systems of Nigeria. However, anthropogenic activities also significantly mobilize and redistribute geogenic fluoride by altering groundwater flow regimes, recharge chemistry, and redox conditions. Intensive groundwater pumping for domestic, agricultural, and industrial supplies leads to changes in the natural flow paths and mixing relationships in aquifer systems. This can mobilize fluoride ions into the deeper fractured basement intervals, bringing it into actively pumped horizons where fluoride concentrations were lower previously (Lar & Gusikit, 2015). Additionally, irrigation return flows and irrigation water applications can also mobilize previously accumulated fluoride in soil profiles, flushing it into underlying aquifers during recharge events. The net effect enhances fluoride concentrations in shallow groundwater beneath irrigated areas relative to undisturbed settings. This process is relevant in northern Nigerian agricultural zones. Nevertheless, the quantitative documentation of this mechanism is still limited in Nigerian studies.

Another anthropogenic factor driving the release and mobilization of fluoride is the application of phosphate fertilizer which acts as a direct source (commercial fertilizers often contain fluoride as impurity in phosphate rock sources) along with promoting the mobilization of geogenic fluoride indirectly. The direct inputs are relatively minor given the typical fertilizer application rates per year. However, phosphate additions alter soil and groundwater chemistry in ways that enhance geogenic fluoride mobility. It has been observed that phosphate competes with fluoride for adsorption sites on iron and aluminium oxyhydroxides, releasing previously adsorbed fluoride (Cai et al., 2012; Habuda-Stanić et al., 2014; Li et al., 2016; Sujana & Anand, 2010; Sujana et al., 2009). This is further explained through Eq. 10:

10 $$\equiv Fe - F_{(surf)} + PO_{4(aq)}^{3 - } \rightleftharpoons \equiv Fe - PO_{4(surf)} + F_{(aq)}^{ - }$$

This ligand exchange process operates most effectively under near-neutral to slightly alkaline pH conditions typical of Nigerian agricultural soils receiving lime amendments alongside fertilizer inputs. Consequently, the agricultural zones where irrigation is practiced may exhibit elevated fluoride despite underlying geology not inherently predisposed to high concentrations (Ndukwe et al., 2019). On the other hand, mining activities in Nigeria expose fresh mineral surfaces and alter weathering rates. These disturbed materials can release fluoride more rapidly than undisturbed bedrock due to increased reactive surface area and enhanced water infiltration through the fractured waste dumps. Additionally, mine dewatering operations lower water tables and alter groundwater flow directions, potentially mobilising isolated high-fluoride zones. However, quantifying the specific contributions of mining to fluoride contamination is yet challenging given the pervasive natural fluoride enrichment in Jos Plateau water systems (Lar & Gusikit, 2015) and other regions across Nigeria. Bright and Chibuzo (2018) noted that water treatment processes influence fluoride concentrations in municipal supplies for Enugu, Southeastern Nigeria, suggesting occasional anthropogenic modification of the natural levels. However, the predominance of naturally elevated fluoride in northern basement terrains versus consistently low fluoride in southern sedimentary provinces despite similar industrial development patterns indicates that geogenic processes dominate Nigerian fluoride distribution (Akpata et al., 2009; Dibal et al., 2012).

#### Ghana

Research on groundwater fluoride in Ghana dates back to the 1990s’, when studies were conducted to determine fluoride levels in groundwater sources (Apambire et al., 1997). Elevated fluoride concentrations have been reported mainly in the Upper East, North East, and Northern regions (Yidana et al., 2012). High fluoride levels in these regions are primarily attributed to the geological composition of rocks containing abundant fluorine-bearing minerals (Sunkari et al., 2018). In addition, anthropogenic activities such as mining, industrial activities, and the use of fluorine-containing fertilizers have also been reported to contribute to groundwater fluoride contamination (Zango et al., 2019).

Numerous studies in Ghana were conducted to investigate fluorosis-endemic areas and assess the effects of groundwater fluoride on public health (Araya et al., 2022; Sunkari & Ambushe, 2024; Sunkari et al., 2022, 2023a; 2023b; Zakaria et al., 2021). Most of these studies focus on local scales, including villages, municipalities, and districts in northern Ghana. Several regions, including Ashanti, Central, Bono East, Ahafo, Oti, Volta, Western North, and Western regions, are yet to be extensively studied for fluoride levels. These areas, dominated by carbonate lithologies, granitoids, and volcanic rocks, are likely to contain elevated fluoride in groundwater.

**Fig. 9:**
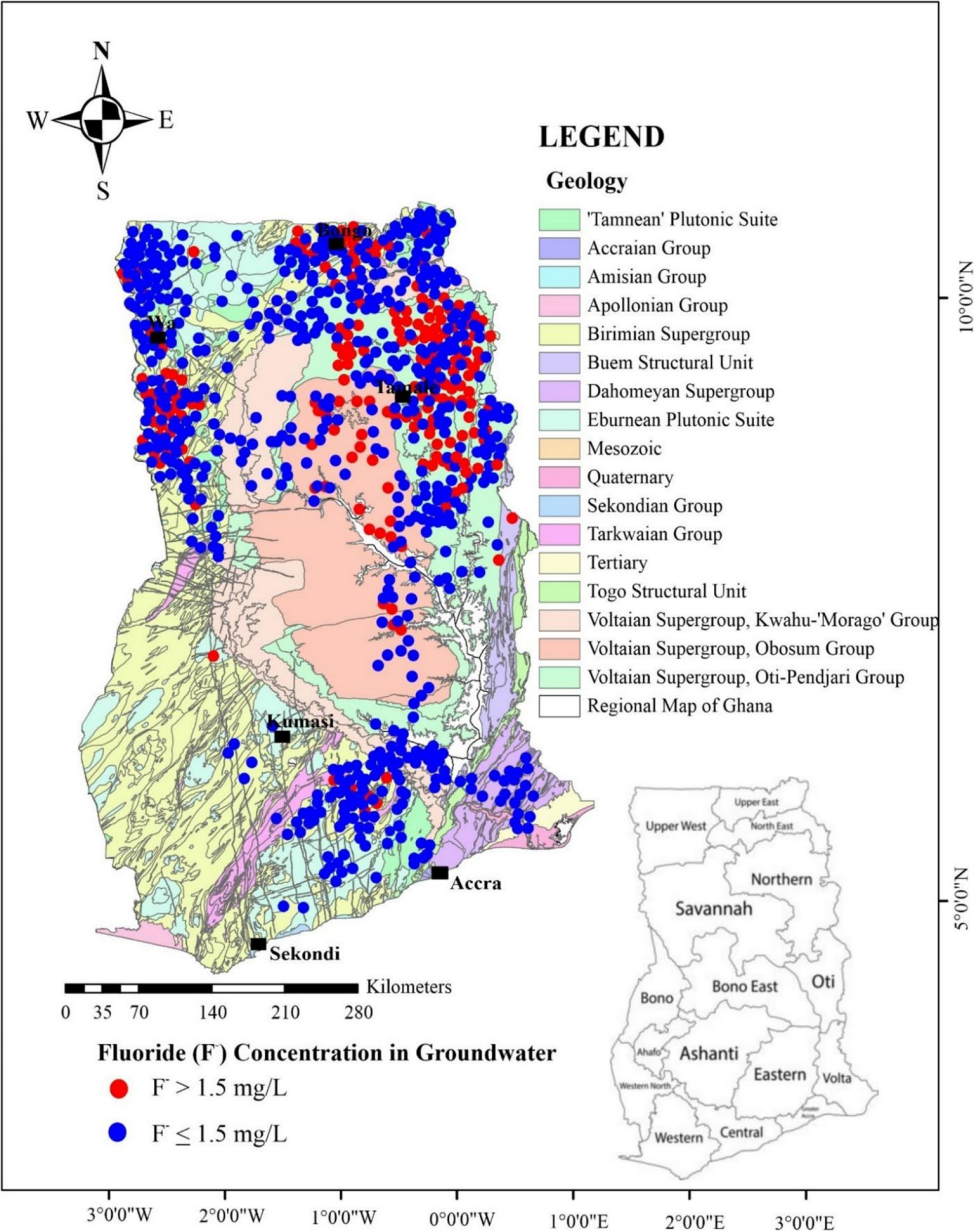
Fluorosis endemic zones in relation to geological provinces of Ghana, based on groundwater fluoride concentration data (N = 3,234) compiled from Araya et al. (2022) and Sunkari and Ambushe (2024; open access)

Geologically, Ghana is composed of the Voltaian Supergroup, Birimian Supergroup, Tarkwaian Supergroup, Buem Structural Unit, Togo Series, Dahomeyan, and Tertiary Sedimentary Basins (Fig. 9). High fluoride concentrations have been reported in aquifers within the Voltaian Supergroup (Sunkari et al., 2020, 2023a & 2023b; Zango et al., 2019) and the Birimian Supergroup (Craig et al., 2015a; Sunkari & Abu, 2019; Sunkari et al., 2018; Zango et al., 2021). However, limited documentation exists regarding high fluoride in other geological formations.

Rocks in northern Ghana are known to contain fluorine-bearing minerals such as fluorite, sellaite, apatite, muscovite, and hornblende (Apambire et al., 1997; Sunkari et al., 2018). Fluoride concentrations in groundwater are generally positively correlated with HCO_3_^¯^ and Na^+^, and inversely correlated with Ca^2+^ (Sunkari & Abu, 2019; Zango et al., 2019, 2021). For example, in the Upper East Region, alkaline water with pH up to 8.6, high HCO_3_^¯^, and moderate EC facilitates the dissolution of fluorite, resulting in elevated fluoride levels (Apambire et al., 1997). Climatic conditions also influence fluoride mobilization; in the Northeast Region, prevailing arid conditions slow groundwater movement, prolonged reaction time with fluorine-bearing rocks, enhances the dissolution of fluoride (Zango et al., 2019).

Several fluoride hotspots have been identified in Ghana (see Table 3, Fig. 9 and Supp. Table S5). In the Upper East Region, the Bongo, Bawku, Vea, and Anayari catchments exhibit fluoride concentrations ranging from 0.10 to 5.0 mg L^−1^, exceeding the WHO guideline (Craig et al., 2015a; Sunkari & Abu, 2019; Zango et al., 2021). The North-East Region, extending from north of Bongo District to eastern Gonja District, is considered the most fluorosis-endemic region in Ghana (Araya et al., 2022; Sunkari & Ambushe, 2024), with fluoride levels ranging from 0.01 to 13.29 mg L^−1^ (Araya et al., 2022; Sunkari & Ambushe, 2024; Sunkari et al., 2022; Zango et al., 2019). This region is largely underlain by Birimian meta-sediments and Upper Voltaian sediments, enriched in fluorine-bearing minerals.

Another hotspot is the Northern Region, particularly the Savelugu-Nanton and Gushegu districts, where fluoride concentrations in drinking water reach 4.1 mg L^−1^, with some sources exceeding 11.0 mg L^−1^ (Anku et al., 2009; Salifu et al., 2012; Sunkari et al., 2023a & 2023b; Yidana et al., 2012). Supp. Table S5 provides details of studies conducted in Ghana’s fluorosis-endemic zones.

#### Ethiopia

Groundwater is a major source of drinking water in many regions of Ethiopia. Fluoride levels exceeding the WHO guideline (1.5 mg L^−1^) have been recorded across the country, but are significantly higher in the Rift Valley system and lowland areas affected by recent volcanic activity (Kloos & Tekle Haimanot, 1999; Onipe et al., 2020; Tekle-Haimanot, 2005). Tekle-Haimanot (2005) reported that, out of 10 million people living along the Ethiopian Rift Valley, about 8.5 million are exposed to fluorosis. The Rift Valley system spans several nations, including Ethiopia, and approximately 41% of high fluoride occurrences in the country are within this rift system (Table 3). Extensive fault networks in the valley create conditions that allow surface water to percolate deeply (Eyayu et al., 2018). Fluoride dissolves more rapidly on the Rift Valley floor due to the region’s significant hydrothermal activity.

The Rift Valley of Ethiopia consists mainly of younger volcanic rocks associated with active volcanoes, containing fluorite and fluorapatite as major components of the basalts. The presence of acidic volcanic rocks and the high temperature of geothermal fluids are considered major drivers of elevated fluoride levels in Ethiopian groundwater (Ayenew, 2008; Onipe et al., 2020). Bianchini et al. (2020) reported that fluoride occurrence in Main Ethiopian Rift waters is unrelated to anthropogenic activities. In Ethiopia, natural processes, such as rock weathering, are the primary sources of fluoride in groundwater. In some areas, rocks contain minerals like fluorite, which release fluoride ions upon weathering. According to Brunt et al. (2004), fluoride concentration in groundwater varies regionally depending on factors such as aquifer material, pH, geology, weathering rate, aquifer depth, contact time, rainfall, and temperature. Temperature, in particular, has been identified as a major driver of groundwater fluoride levels in Ethiopia.

Rango et al. (2012) analysed water samples from various sources, including groundwater wells, cold springs, geothermal springs, and lakes in the Ziway-Shala basin, the Abaya-Chamo basin, and a small catchment in Awasa within the central Main Ethiopian Rift system. Fluoride concentrations were recorded in the range of 1.1–68.0 mg L^−1^ in groundwater, 2.0–65.0 mg L^−1^ in geothermal springs, 0.2–22.0 mg L^−1^ in cold springs, and 1.8–435.0 mg L^−1^ in lakes. Fluoride concentrations exceeding 5.0 mg L^−1^ are common in lakes, boreholes, hot springs, and shallow wells within the rift valley system, whereas lower levels (< 1.5 mg L^−1^) are typically found in cold springs and rivers (Kloos & Tekle Haimanot, 1999; Tekle-Haimanot et al., 2006). Supp. Figure S6 illustrates the recorded fluoride levels within and outside the Rift Valley system.

Generally, deep boreholes exhibit higher fluoride levels than shallow wells (Onipe et al., 2020; Tekle-Haimanot et al., 2006). Similarly, hot springs record higher fluoride concentrations than cold springs (Supp. Table S6), indicating that water temperature plays a key role in fluoride mineralization. Haji et al. (2018) reported similar findings in the southern part of the Main Ethiopian Rift Valley, where hot springs reached fluoride levels up to 57.5 mg L^−1^ at 81 °C, compared to 1.7 mg L^−1^ in cold springs at 22.5 °C.

#### Kenya

High fluoride in Kenyan drinking water resources has been known and documented for quite some time. Early scientific works date back to the 1950s’ (Williamson, 1953), when high concentrations were reported in boreholes, shallow wells, and lake water. Williamson (1953) noted the highest concentrations of fluoride in waters within the Rift Valley region, reporting levels as high as 450 mg L^−1^ in Lake Nakuru and widespread dental fluorosis, mostly affecting children. Later, Nair et al. (1984) observed that more than 50% of groundwater samples from across the country had fluoride levels greater than 1.0 mg L^−1^, with one-fifth exceeding 5.0 mg L^−1^.

In the Kenyan Rift Valley, fluoride concentrations of up to 5.9 mg L^−1^ have been reported in 50% of boreholes in Turkana County in the north (Mbugua et al., 2022; Rusiniak et al., 2021), where high fluoride levels were associated with geogenic sources and, in part, anthropogenic sources such as oil drilling. Alkaline volcanic rocks and sediments in the Rift Valley can accumulate high fluoride in minerals and volatiles (Mwiathi et al., 2022). Fluoride concentrations between 3,750 and 6,000 mg/kg were reported in cordierite and muscovite present in vitreous volcanic rocks in Nakuru County (Olaka et al., 2016). Volcanic emissions and the mixing of hydrothermal waters have also been reported to contribute to high fluoride in Rift Valley groundwater (Mwiathi et al., 2022; Olaka et al., 2016). Lower fluoride concentrations in secondary fluoride minerals (including illite and kaolinite) compared to primary minerals (cordierite and muscovite) suggest that rock alteration is a major process governing the release of fluoride (Olaka et al., 2016).

**Fig. 10:**
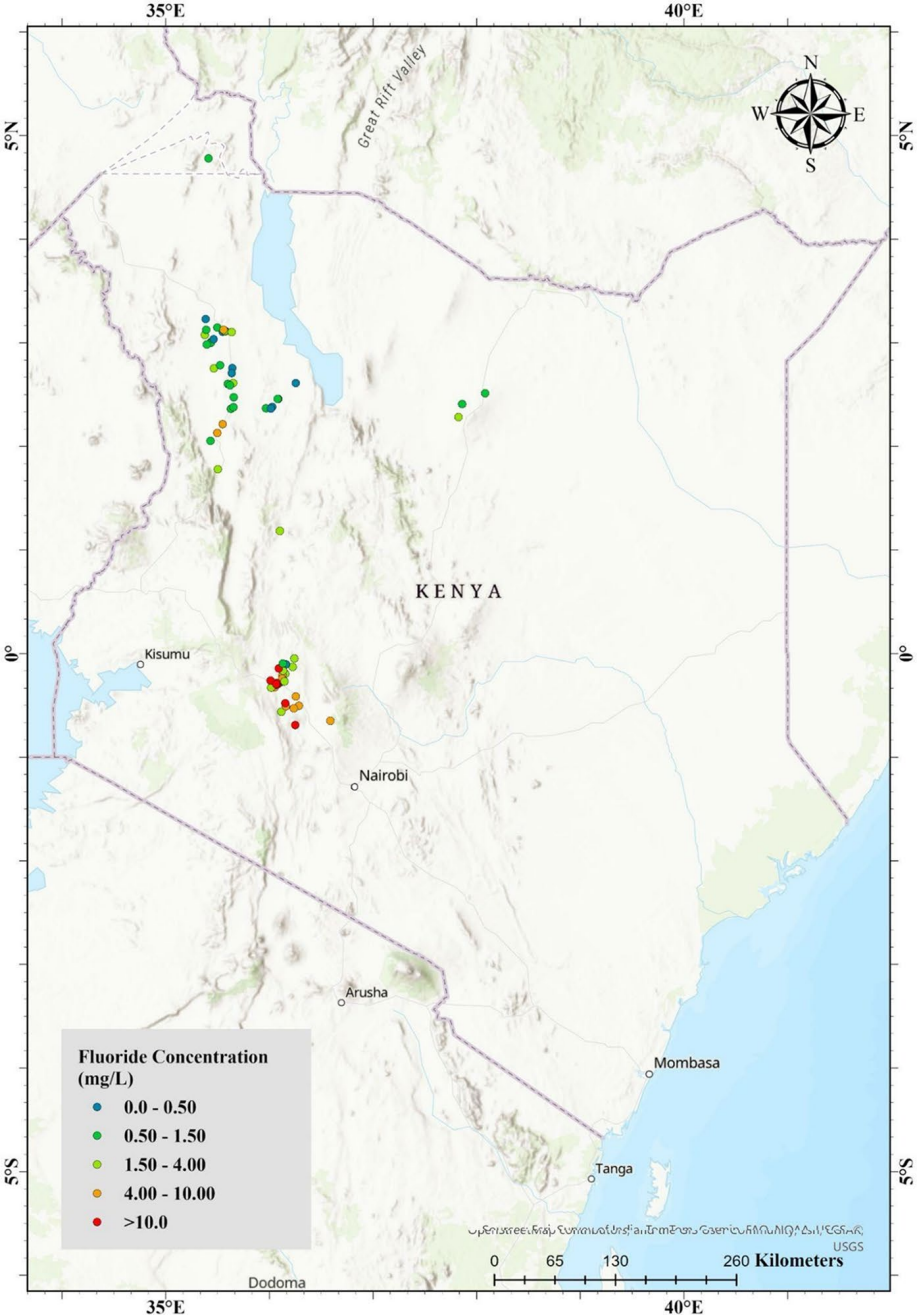
Fluoride distribution in different aquifers in Kenya (N = 70)

In the central Rift Valley, within Nakuru, Naivasha, and Baringo Counties, high fluoride concentrations of up to 75.0 mg L^−1^ have been reported in up to 73% of both shallow and deep wells (Gevera & Mouri, 2018; Jirsa et al., 2013; Moturi et al., 2002; Mutonga, 2014). In the southern Rift Valley, concentrations of up to 15.0 mg L^−1^ have been reported in Kajiado County (Induswe et al., 2018). High fluoride is also reported in other regions covered by Rift Valley volcanic rocks, such as Kiambu, Nairobi, and Machakos Counties, with boreholes showing fluoride concentrations up to 10.5 mg L^−1^ (Coetsiers et al., 2008; James, 2016; Nzeve & Mbate, 2021; Olonga et al., 2015). Rift Valley lakes exhibit higher fluoride concentrations (up to 140 mg L^−1^) than groundwater or rivers (Gaciri & Davies, 1993; Olaka et al., 2016; Fig. 10 and Table 3).

Another high-fluoride region in Kenya is the area covered by metamorphic rocks of the Mozambique Mobile Belt (MMB), which runs north–south in the eastern part of the country. Fluoride concentrations of up to 8.2 mg L^−1^ have been reported in over 50% of shallow wells and boreholes in Kitui, Machakos, and Makueni Counties (Gevera et al., 2020; Mailu, 1994; Mbithi, 2017; Nair et al., 1984). Although these high fluoride levels were known for some time (Nair et al., 1984), water management reports in the 2000s (Japan International Cooperation Agency [JICA], 2004) classified some boreholes above the WHO safe limit as safe, thereby increasing the risk of exposure for local communities. In the MMB metamorphic region, water-soluble fluoride concentrations of up to 3.47 mg/kg were reported in farm soils in Makueni County, where fluoride-rich minerals, including apatite, biotite, and muscovite, constitute 23% of the soil mineralogy (Gevera et al., 2022a).

Minor occurrences of fluoride in Kenyan drinking water, with less spatial extent compared to the Rift Valley and the MMB, have been reported in Kisumu County in the western part of the country, where concentrations of up to 11.0 mg L^−1^ were reported in 30–81% of shallow and deep wells (Kanoti et al., 2019).

#### Tanzania

**Fig. 11:**
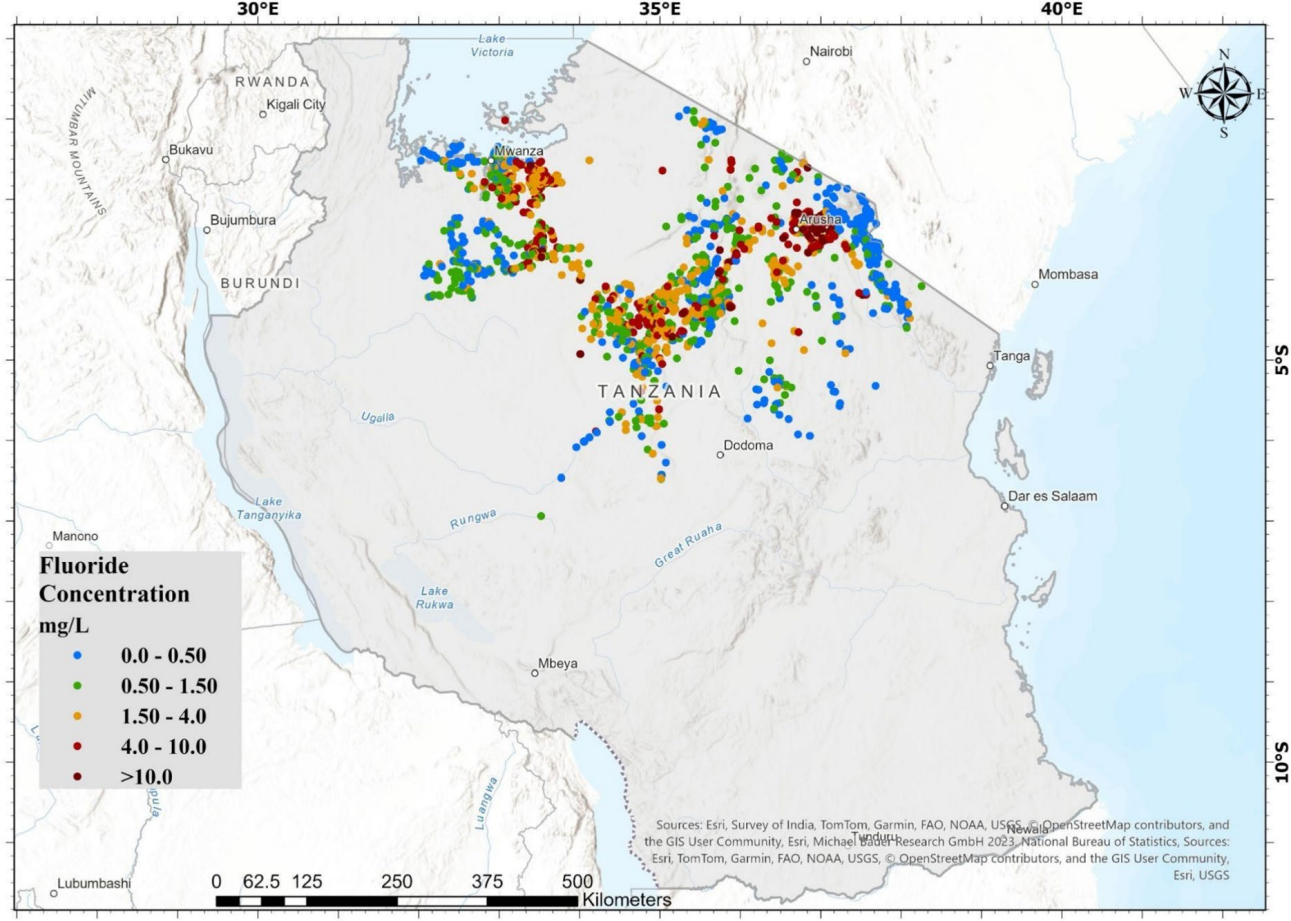
Fluoride distribution in the groundwater of Tanzania (N = 2491)

In some regions of Tanzania, around 85% of water demand is met by groundwater accessed through shallow wells, boreholes, and springs, where elevated concentrations of fluoride have rendered approximately 70% of the sources unsafe, especially for drinking purposes. Most groundwater sources used for drinking in these regions exceed WHO limits. Regions close to or within the EARV and volcanic areas are highly contaminated. Past studies have reported high fluoride levels in around 53% of Tanzanian regions (Mjengera & Mkongo, 2003). The regions severely affected by excessive fluoride in their water sources include Mara, Geita, Mwanza, Shinyanga, Simiyu, Singida, Arusha, Kilimanjaro, and Manyara. Other regions moderately affected include Tanga, Dodoma, Tabora, Kigoma, Katavi, and Rukwa (see Fig. 11, Supp. Figure S7 and Table 3). Fluoride levels in these regions range between 0.01 and 74.0 mg L^−1^, especially in EARV and volcanic areas (Ijumulana et al., 2020). About 90% of the population in these regions exhibit mild to severe dental fluorosis due to prolonged consumption of groundwater with fluoride exceeding 1.5 mg L^−1^ (Vuhahula et al., 2009).

Fluoride occurrence in the EARV regions and volcanic areas is spatially variable, affecting local communities differently, particularly in rural areas where people rely on untreated water. Fluoride in these regions is mobilized from Tertiary–Quaternary volcanic rocks and Tertiary–Quaternary unconsolidated sediments around major stratovolcanoes and within the EARV graben, respectively (Bennett et al., 2021, 2022; Tomašek et al., 2022; Supp. Figure S8). Geochemical processes triggering the severity of fluoride risk include weathering of fluorine-bearing volcanic rocks, dissolution of fluoride-rich minerals, evaporation, geothermal water mixing, ion exchange, and evapo-transpiration (Berger et al., 2016; Ijumulana et al., 2020; Olaka et al., 2016; Vithanage & Bhattacharya, 2015).

The high spatial variability of fluoride concentrations in groundwater in the EARV regions and volcanic areas is controlled by the natural slope, which determines the contact time between water and rock during recharge (Ghiglieri et al., 2010; Ijumulana et al., 2022). This brings variability in fluoride concentration, which is commonly reported globally. However, some groundwater sources have extremely low fluoride levels, even below 0.5 mg L^−1^, posing a risk of dental caries (Dissanayake, 1991). Usually, these low levels occur in perched aquifers within primary recharge zones around stratovolcanoes or within river valleys. For example, a study by Ijumulana et al. (2021) reported the lowest fluoride levels around Mt. Kilimanjaro, where approximately 1 million people are at risk of dental caries.

Like Kenya, Malawi, and Ethiopia, groundwater with high fluoride concentrations is also reported from the EARV, particularly in northern and southwestern Tanzania. It is estimated that about 90% of the population living in the EARV regions is affected by dental fluorosis (Ijumulana et al., 2022; Kimambo et al., 2019; Ligate et al., 2021). Gutierrez et al. (2023) reported that about 200 million people, mainly in rural areas, are exposed to fluorosis. In this context, Kimambo et al. (2019) reviewed fluoride in African groundwater and found high levels mainly in the EARV. Bennett et al. (2022) and Ligate et al. (2021) reported that the Tanzanian permissible limit is 4.0 mg L^−1^, which is higher than the WHO safe limit. A similar high permissible limit exists in Malawi (6.0 mg L^−1^; Addison et al., 2020a). These elevated limits reflect the widespread occurrence of high-fluoride drinking water sources and limitations in complying with WHO (2017) guidelines.

Furthermore, Ijumulana et al. (2021) reported elevated fluoride levels in groundwater mainly within the EARV and in mountainous terrains such as Meru and Kilimanjaro. Ijumulana et al. (2020) also reported very high fluoride levels in the vicinity of EARV but also found that fluoride levels vary widely and safe aquifers also occur in the region. Nakayama et al. (2022) found that the extent of weathered aquifers is proportional to fluoride levels along the EARV” with “extent of weathered aquifers is positively associated with fluoride levels along the EARV, highlighting the importance of water-rock interaction and weathering intensity. Ijumulana et al. (2020) observed that fluoride is largely derived from geogenic sources dominated by fluorine-bearing minerals, which is consistent with findings by Bennett et al. (2022). Along the flanks of Mount Meru, low fluoride occurs in pyroclastic and lava deposits, whereas high fluoride is mainly associated with debris avalanches, ash, alluvial fans, and lake deposits” with “low fluoride occurs predominantly in pyroclastic and lava deposits, whereas elevated fluoride concentrations in groundwater are associated with ash-rich sediments, alluvial fans, and lacustrine deposits. (Bennett et al., 2022).

#### Malawi

Addison et al. (2020a) thoroughly examined fluoride levels in Malawi’s water resources, reporting very high fluoride concentrations in hot springs. Their studies have significantly advanced the understanding of fluoride distribution in the country (Addison et al., 2020a, 2020b; Table 3), highlighting the need for regular groundwater monitoring. Msonda et al. (2007) found that over 50% of the water samples in their study were contaminated with fluoride from geogenic sources.

**Fig. 12:**
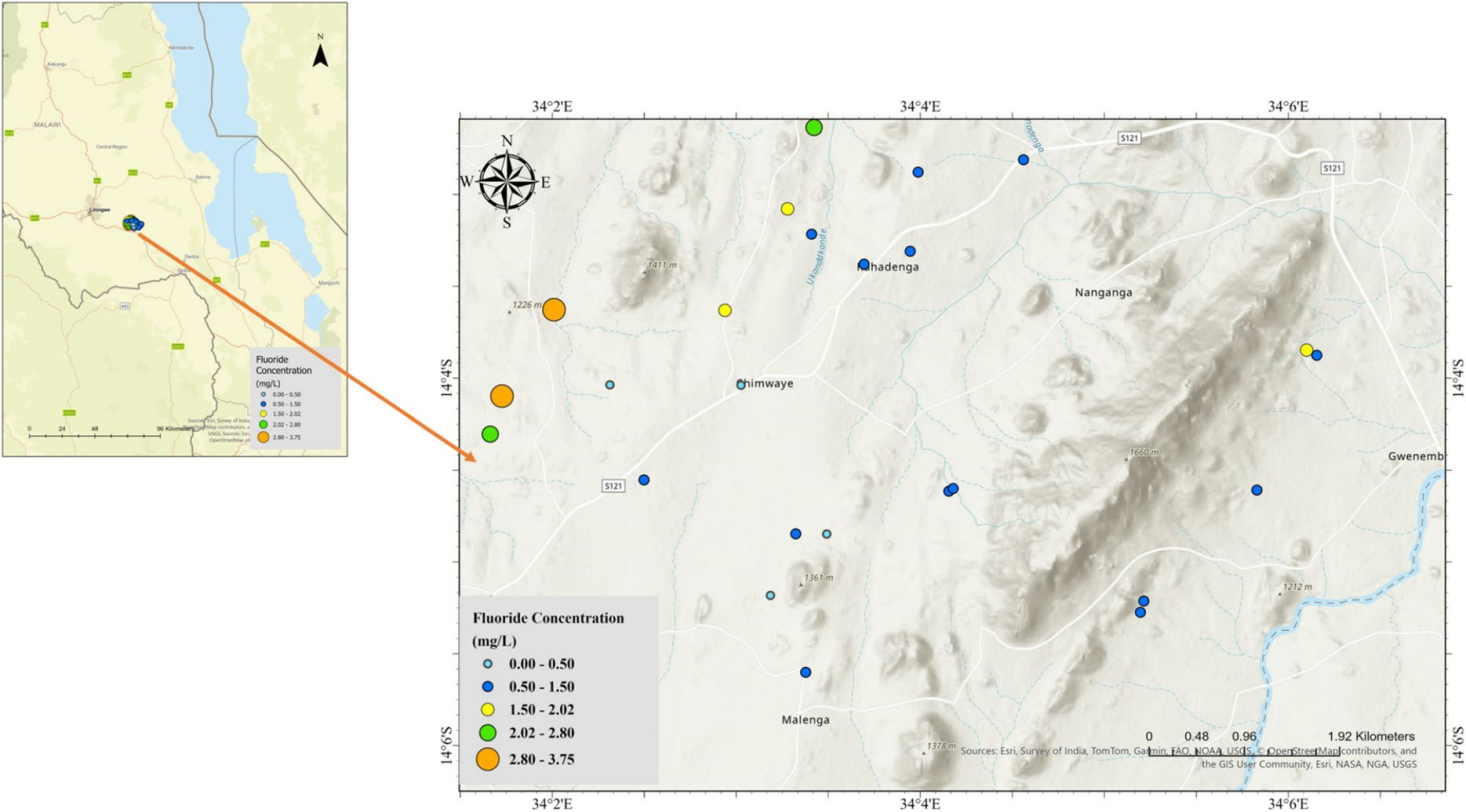
Fluoride in the groundwater of Malawi (Addison, 2021). (N = 40)

Sajidu et al. (2008) observed that bauxite could be highly effective for defluoridation in Malawi, due to the presence of kaolinite and gibbsite minerals, which can remove up to 94% of fluoride under specific pH conditions. Addison et al. (2020b) identified that the existing rocks are the primary source of fluoride and quantified the geological control on fluoride contamination. The authors also observed that groundwater occurring in augen gneiss is more contaminated than groundwater occurs in other lithologies. Andreah et al. (2023) also reported elevated fluoride concentrations in Malawi. Addison (2021) produced a fluoride prediction map for Malawi (Fig. 12) and emphasized the need to reduce the current maximum permissible fluoride level of 6.0 mg L^−1^.

### Middle East

#### Yemen

Yemen is one of the Middle Eastern countries severely affected by fluorosis (Abdulmohsen Saleh Al-Amry, 2009; Aqeel et al., 2017; Kadir & Al-Maqtari, 2010; Salman, 2006; Table 3). Considerable attention has been given to the large number of children and adolescents affected, with dental fluorosis identified as the most prevalent in the country (Aqeel et al., 2017; Kadir & Al-Maqtari, 2010; Salman, 2006).

Most rural areas in Yemen rely primarily on groundwater from wells for drinking and domestic use. Viswanatham (2008) reported that several wells in rural areas have extremely high fluoride concentrations (up to 32 mg L^−1^), although the sources of such elevated levels remain undocumented. Other studies indicate that the highest fluoride concentrations occur in the governorates of Sana’a, Ibb, Dhamar, and Taiz (UNICEF, 2008; Van der, 1997). In some parts of Dhamar Governorate, fluoride concentrations in groundwater wells were found to reach up to 1.84 mg L^−1^ (Aizari et al., 2021). Adolescents living in southern provinces like Taiz and Al-Dhalea were about twice as likely to develop dental fluorosis compared to those in other regions. Recent studies reported fluoride concentrations in some groundwater wells in the Al-Dhalea basin, Al-Dhalea Province, as high as 18.0 mg L^−1^ (Al-Amry et al., 2020).

The Natural Water Resources Authority of Taiz (NWRA/Taiz) conducted two consecutive studies on drinking water fluoride levels across all 14 districts of Taiz Governorate. The results indicated that water in eight districts are within the safe fluoride limits, whereas in Mawyah and At-Taizyah districts, fluoride concentrations ranged from 1.7 to 10.0 mg L^−1^, respectively (Supp. Figure S9). Aqeel et al. (2017) further studied fluoride contamination in Taiz City, revealing high fluoride levels in over 80% of the city, with concentrations reaching up to 3.6 mg L^−1^ (Supp. Figure S10).

#### Iran

**Fig. 13:**
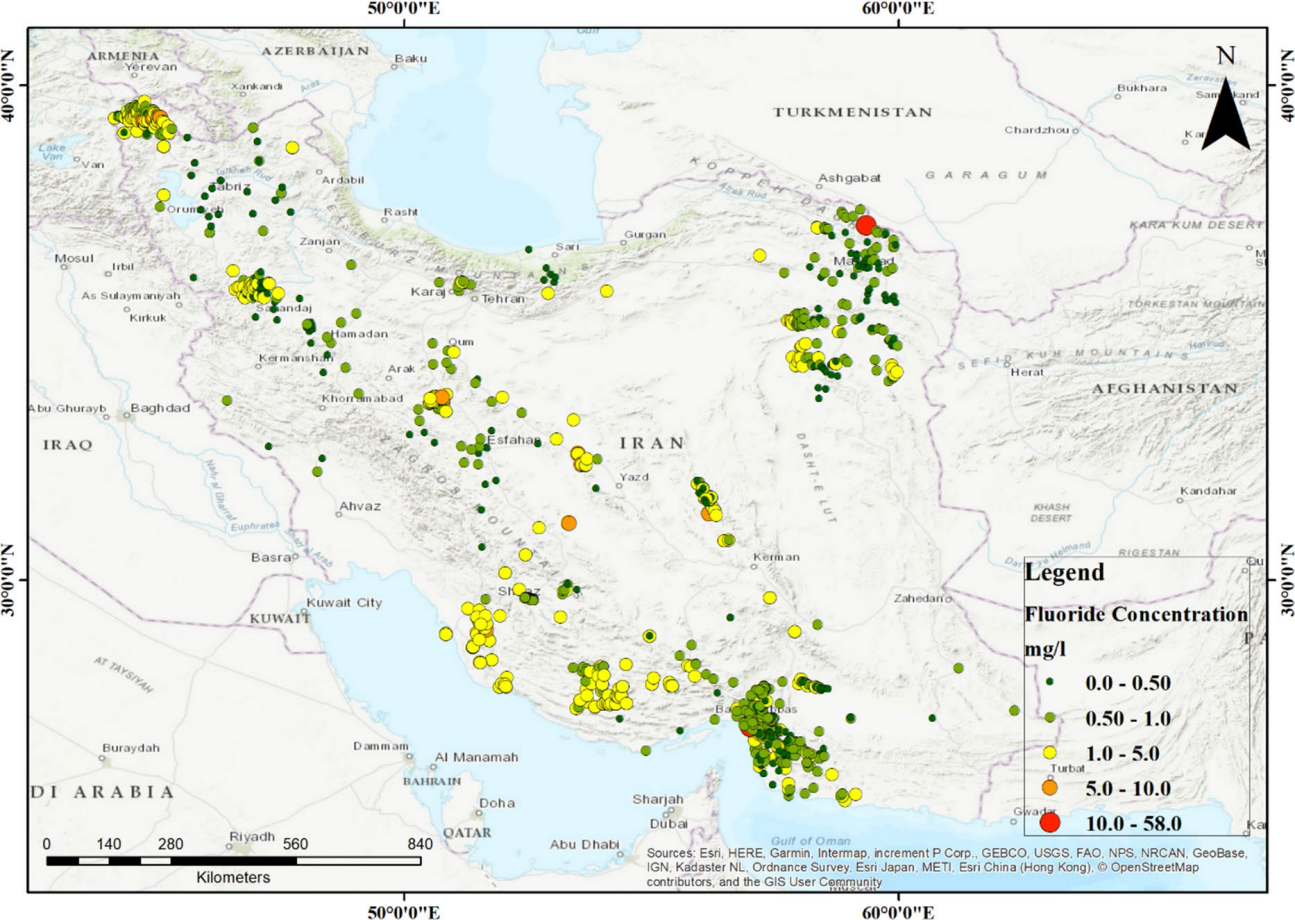
Fluoride in the groundwater of Iran (N = 1039)

Groundwater is the major source of drinking water in Iran, and fluoride exposure is frequently reported. Typically, fluoride concentrations in groundwater are below 1.0 mg L^−1^ in most regions (see Table 3). Mesdaghinia et al. (2010) reported a mean nationwide fluoride value of 0.47 mg L^−1^. A systematic review of national and sub-national drinking water fluoride concentrations from 1990 to 2015 showed an average of 0.65 ± 0.38 mg L^−1^ (Taghipour et al., 2016). More recent reviews indicate an average of 0.51 mg L^−1^ in the country (Keramati et al., 2019; Fig. 13).

Fluoride anomalies in Iran’s groundwater are primarily geogenic, although hydrogeochemical and climatic factors also play significant roles. In certain industrial areas, anthropogenic activities such as use of fertilizer and aluminum smelting have reported to have negligible effects (Dobaradaran et al., 2014; Mohsen et al., 2021).

Numerous studies have documented the health impacts of fluoride in drinking water across Iran, including dental and skeletal fluorosis in high-fluoride areas and increased risk of dental caries in low-fluoride regions (Aghaei et al., 2015; Dehbandi et al., 2018; Derakhshani et al., 2017; Kheradpisheh et al., 2018; Sabokseir & Golkari, 2023; Seraj et al., 2012). Abtahi et al. (2019) used disability-adjusted life years (DALYs) to assess the burden of skeletal and dental fluorosis. DALYs attributable to dental caries could be reduced through water fluoridation in provinces such as Tehran, Isfahan, Khorasan Razavi, East Azarbaijan, and Guilan, where fluoride levels are below the safe limit. Conversely, DALYs were concentrated in Fars, Bushehr, West Azarbaijan, and Hormozgan, where fluoride-related health problems are most prevalent. Due to Iran’s climatic and temperature diversity, as well as its varied demography, dietary habits, and ethnic composition, water intake rates differ substantially across the country. Consequently, the level of fluoride exposure for individuals varies in different regions of Iran. This is also true for other countries. A national study (Zazouli et al., 2017) directly addresses this issue by quantifying the relationship between climate and optimal fluoride concentration in drinking water where the authors calculated province-specific optimal fluoride concentrations to be in the range of 0.64–1.04 mg L^−1^. It was found that warmer provinces such as Hormozgan, Khuzestan, and Alborz had optimal values (approximately 0.64–0.66 mg L^−1^). This evidence clearly shows that climatic conditions, which directly affect how much water people consume, need to be considered when assessing fluoride exposure risk (HQ). To practically demonstrate this variability and its impact on risk assessment, we have included a worked example in the Appendix (see Supp. Table S7) that explicitly examines how the Hazard Quotient (HQ) varies with changes in water intake (simulating different climatic conditions) and dietary fluoride contributions.

The Bazargan-Poldasht belt, extending from northern West Azarbaijan to eastern Türkiye near the Tendurek volcano, is identified as a prominent endemic fluorosis zone (Yousefi et al., 2019a). Fluoride concentrations in this belt range from 0.3 to 11.4 mg L^−1^ (Aslani et al., 2019; Yousefi et al., 2018, 2019b). Interaction between groundwater and basaltic rock is likely the primary cause of elevated fluoride, facilitated by high HCO_3_^¯^ and Na^+^ concentrations, which enhance weathering of fluorine-rich minerals such as apatite and fluorapatite (Asghari Moghaddam & Fijani, 2008). Additionally, Amiri and Berndtsson (2020) showed that fluorine-bearing minerals play a pivotal role in causing high fluoride concentrations (> 1.5 mg L^−1^), especially in shallow drinking wells of the Urmia Lake aquifer.

High fluoride levels in eastern and northeastern Iran are mainly observed in North Khorasan, Razavi Khorasan, and South Khorasan provinces. In Razavi Khorasan, drinking water fluoride ranges from 0.05 to 2.31 mg L^−1^ (Ghaderpoori et al., 2018; Nourafrouz et al., 2020; Qasemi et al., 2019a, 2019b, 2022). Some areas, including Bardaskan, Khaf, Gonabad, Bajestan, and Sabzevar, exceed WHO safe limits, mostly due to geogenic sources. In contrast, southwestern regions such as Kakhk County exhibit significantly lower fluoride levels, ranging from 0.05 to 0.77 mg L^−1^ (Qasemi et al., 2023).

The fluoride-enriched zone in southeastern Iran includes Sistan and Baluchestan provinces and the southeastern region of Kerman province. Fluoride concentrations in groundwater here range from 0.13 to 4.8 mg L^−1^ and are predominantly associated with Na-Cl, Mg-Cl, and Na-SO_4_ water types (Abbasnejad et al., 2023; Biglari et al., 2016; Naderi et al., 2020). Monitoring in Sistan and Baluchestan indicates the main hotspot of fluoride is in the north and central province, particularly Zahedan County (Biglari et al., 2016). Cold and thermal springs around Bazman volcano exhibit 0.5–3.75 mg L^−1^ fluoride (average 1.66 mg L^−1^; Naderi et al., 2020).

Southern Iran, encompassing Fars, Bushehr, and Hormozgan provinces, is another major fluoride hotspot. In Fars, fluoride in the groundwater ranges from 1.6–6.36 mg L^−1^ (Aghniaei et al., 2017; Amini et al., 2016; Dehghani et al., 2018). Bushehr, with the highest average fluoride in Iran, shows hotspots in Dahtestan, Dayyer, and Tang-e Eram counties, with fluoride up to 4.0 mg L^−1^ (Battaleb-Looie et al., 2013; Dobaradaran et al., 2008, 2018; Keshavarz et al., 2015; Ramezani et al., 2004). Hormozgan’s drinking water fluoride ranges from 0.1–0.9 mg L^−1^; while the risk of fluorosis is negligible, fluoridation is recommended for areas below 0.5 mg L^−1^ (Mohammadpour et al., 2022). High-fluoride groundwater is linked to fluorine-bearing clay minerals, micas, apophyllite, dolomite, evaporites from salt diapirs, and numerous thermal springs. This is intensified by specific water types (Na-Cl, Mg-Cl, Ca–Cl, Na-SO_4_) and chemical evolution along the groundwater flow paths (Balaghi Enalou et al., 2018; Battaleb-Looie & Moore, 2010; Rezaei et al., 2017). Evaporation driven by high temperatures and arid/semi-arid climates also contributes to high fluoride, as confirmed by stable isotope studies (Balaghi Enalou et al., 2018).

Fluoride concentrations in western and central-western Iran have been reported from Lorestan, Hamedan, and Markazi provinces. Rashnoodi et al. (2019) in Lorestan province reported fluoride concentrations ranging from 0.67 to 1.9 mg L^−1^, with over one-third of samples exceeding 1.5 mg L^−1^. Similarly, Salehi Seifabadi et al. (2023) studied groundwater samples from Kabudarahang, Hamadan province, reporting fluoride concentrations between 0.2 and 2.3 mg L^−1^, linked to interactions of water with existing volcanic and alkaline rocks.

Central Iran hosts an endemic fluorosis belt encompassing the provinces of Isfahan, Yazd, and Kerman. Within this belt, the Bahabad-Kouhbanan-Zarand (BKZ) zone is notable for exceptionally high groundwater fluoride concentrations, ranging from 0.1 to 6.0 mg L^−1^, with an average of 0.82 mg L^−1^ (Dehbandi et al., 2017, 2018). In over 1,000 groundwater samples from Yazd province, fluoride concentrations ranged from 0.02 to 1.96 mg L^−1^, averaging 0.66 mg L^−1^, with the highest values observed in Meybod and Ashkezar cities, near industrial areas (Fallahzadeh et al., 2018). Dehbandi et al. (2018) also reported a maximum concentration of 2.35 mg L^−1^ in Bahabad county, in southern Yazd province, known for a high prevalence of dental fluorosis. The most extreme case in Yazd province was in Ardakan county, where fluoride concentrations ranged from 0.9 to 6.0 mg L^−1^, averaging 2.92 mg L^−1^ (Mirzabeygi Rad Fard et al., 2018).

Kerman province shows an even wider range of fluoride concentrations compared to other provinces, ranging from 0.05 to 10.8 mg L^−1^ in Kouhbanan (Fekri & Kasmaei, 2013; Pazand, 2016) and 0.2 to 3.45 mg L^−1^ in Zarand (Dehbandi et al., 2018). Generally, the primary sources of fluoride in central Iran are geogenic, originating from metamorphic and intermediate-acidic igneous rocks, shales, Kiruna-type Fe oxide/apatite deposits, carbonate-hosted Pb–Zn ore deposits, and coal deposits (Dehbandi et al., 2017; Keshavarzi et al., 2010). Evaporation and ion exchange are the main mechanisms controlling fluoride enrichment in the groundwater system (Amiri et al., 2024; Dehbandi et al., 2017, 2018).

Northern Iran, including Mazandaran, Golestan, and Guilan provinces, has a temperate rainforest climate with the highest precipitation levels in the country. For example, Mahmoodlu et al. (2023) reported fluoride concentrations in Golestan province ranged from 0.03 to 0.83 mg L^−1^. To prevent health issues related to fluoride deficiency, water fluoridation is recommended in these areas (Mohammadi et al., 2015). In Sari city, the capital of Mazandaran province, fluoride concentrations in drinking water varied between 0.17 and 0.32 mg L^−1^, with all sources exhibiting fluoride deficiency (Ramezani et al., 2009).

### Europe

#### Türkiye

The first case of endemic dental fluorosis in Türkiye was reported in 1955 in Isparta Province, southwestern Anatolia. In this region, pyroxene, hornblende, biotite, fluorapatite, and glassy groundmass minerals in volcanic rocks were identified as major sources of fluoride (Oruc, 2008). Severe dental and skeletal fluorosis have also been reported in the eastern towns of Doğubeyazıt and Çaldıran, where natural water contains 2.5–12.5 mg L^−1^ fluoride (Şendil & Bayşu, 1973). At the foothills of the young Tendürek volcano, fluoride transported by fumaroles or escaping from devitrified lavas is thought to accumulate on mineral surfaces and subsequently exchange with OH^−^ in high pH fluids (Koç & Karademir, 2021).

In central-west Türkiye, in Kızılcaören village of Beylikova city (Eskişehir Province), drinking water with fluoride concentrations range from 3.9 to 4.8 mg L^−1^, causing widespread dental and skeletal fluorosis. Fluorspar minerals in nearby watersheds are believed to be responsible for the high fluoride levels (Fidanci et al., 1998). In Güllü village (Eşme-Usak, south-central Türkiye), residents aged 10–30 exhibited mild to moderate speckled enamel, with deep well water containing 0.7–2.0 mg L^−1^ fluoride (Table 3). Amorphous microscopic fluorite in the limestone of Lake Pliocene is considered a possible source of fluoride in water (Can, 2022).

Very high fluoride concentrations were detected in the public water supply in 1990 across many areas of the city. Since 1995, with the introduction of water from Lake Egirdir, fluoride levels in public water in several districts decreased to WHO safe limits by 2003. Fluoride levels in Lake Gölcük and nearby groundwater remain high (1.4–4.6 mg L^−1^), exceeding WHO guidelines due to the dissolution of fluoride-containing volcanic fluorites. Fluoride concentrations vary seasonally depending on contact between groundwater and volcanic rocks (Davraz et al., 2008).

In northeastern Türkiye, Vural and Gundogdu (2020) analysed stream waters in the Gökdere Valley Drainage Network (Gümüşhane Province), reported fluoride concentrations from 0.19 to 9.87 mg L^−1^, averaging 3.7 mg L^−1^. In southeastern Türkiye, wells at 100–150 m depth in Sarım and Kataş villages (Şanlıurfa Province) had 1.95 mg L^−1^ fluoride (Karabulut & Atasoy, 2019). Tokatli and Güner (2018) reported 0.006–0.57 mg L^−1^ fluoride in Havsa district, while Baba and Ayyildiz (2006) found 3.9–4.8 mg L^−1^ in Beylikova villages and 2.0–9.2 mg L^−1^ in other areas.

The Bayındır-Kaman (Kırşehir) area has groundwater fluoride around 2.6 mg L^−1^. Other areas with fluoride contamination include Doğubeyazit (Ağrı), Muradiye (Van), and Habiller (Edirne). In Güllü village (Uşak), 13 surface and groundwater samples contained 0.7–2.0 mg L^−1^ fluoride (average 1.35, median 1.60; Baba & Ayyildiz, 2006). Varol et al. (2021) studied Salda Lake Basin (Burdur) and found fluoride between 0.01–5.87 mg L^−1^ in the wet season and 0.03–0.27 mg L^−1^ in the dry season. Hydrochemical facies of Savcılı-Büyükoba (Kırşehir) geothermal waters are Na-Cl type, whereas cold waters are Ca-HCO_3_ type. In 2014, fluoride concentrations ranged 0.09–7.9 mg L^−1^ (Yurteri & Simsek, 2017).

**Fig. 14:**
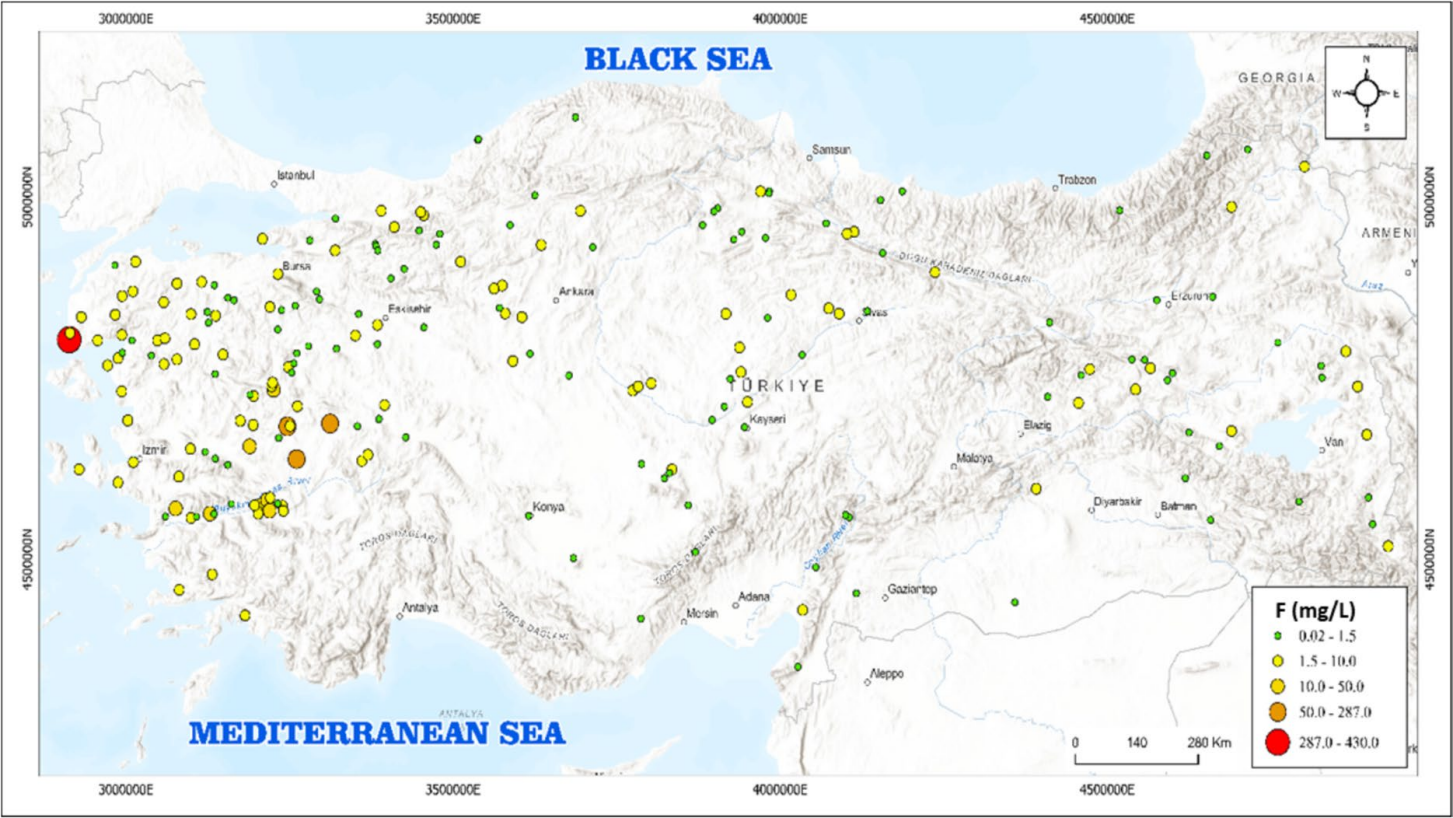
Distribution of fluoride in thermal and mineral water spring groups in Türkiye. The map in this study was drawn based on fluoride values measured (N = 200). The fluoride values were measured in both cold and warm waters. Türkiye has higher fluoride values, especially in warm waters. This is related to the geological structure of the area

Figure 14 shows fluoride distribution in thermal and mineral water springs across Türkiye, ranging from 0.02 mg L^−1^ to 430 mg L^−1^. The maximum concentration of 430 mg L^−1^ was recorded in a geothermal spring in Çanakkale Province (Tuzla district, northwestern Türkiye), which is not used for drinking.

#### Estonia

Estonia is situated on the north-western margin of the East European Platform. The chemical composition of groundwater used for drinking is closely related to local geology. Water–rock interactions can increase the concentration of certain dissolved chemical species, including fluoride, necessitating treatment to ensure safe and high-quality drinking water. Water management in Estonia, including drinking water quality, is strictly regulated by law (Water Act, 2019) and aligns with European Union requirements. About 1,300 waterworks operate under state supervision, ensuring that delivered water is regularly and thoroughly monitored. According to EU Directive 98/83/EC (European Union, 1998) and Estonian drinking water standards (Minister of Social Affairs: Estonia, 2019), the maximum permissible fluoride concentration in drinking water is 1.5 mg L^−1^.

Analysis of the regional distribution of fluoride in tap water (Karro et al., 2006) shows significant variation across the country. Southern and north-eastern Estonia is characterized by low fluoride levels (< 0.8 mg L^−1^). Northern and central Estonia generally receive water with optimal fluoride concentrations (0.7–1.3 mg L^−1^). Elevated fluoride levels are most commonly observed in southwestern and western Estonia, with concentrations reaching up to 6.95 mg L^−1^.

**Fig. 15:**
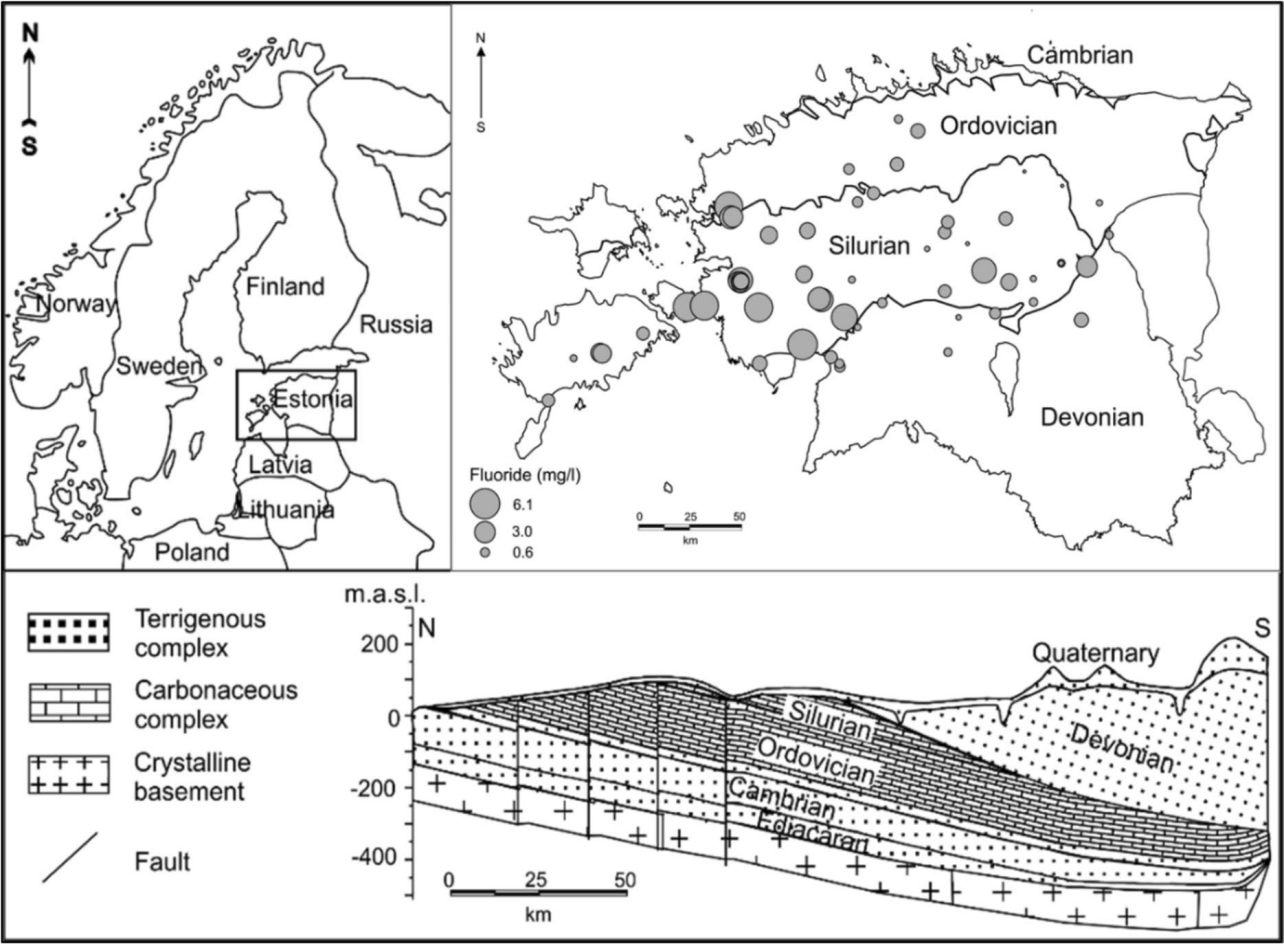
Location, geological map and a north–south geological cross-section of Estonia. The spatial distribution of fluoride in Silurian-Ordovician aquifer system is shown in the background of the geological map (N = 60)

Low-fluoride areas in southern Estonia correspond to the outcrop of Devonian sedimentary rocks, where the primary drinking water source is the Middle-Devonian terrigenous aquifer system (Fig. 15). Northern Estonia relies on Cambrian–Vendian and Ordovician–Cambrian terrigenous aquifer systems, often accessed through wells penetrating Ordovician carbonate rocks. The highest fluoride concentrations are observed in areas underlain by Silurian and Ordovician limestones and dolomites, where the Silurian–Ordovician aquifer system is the only drinking water source. Naturally high fluoride is also found along the northern outcrop line of Devonian rocks, where hydraulically connected Devonian and Silurian strata form the Devonian–Silurian aquifer system.

Tap water analyses provide essential information for assessing potential health risks. In many cases, groundwater from different hydrogeological units is mixed within water supply systems, making it difficult to directly associate fluoride concentrations with specific aquifers. Nevertheless, in certain regions, the fluoride content of drinking water closely reflects the local geology.

Statistics of fluoride concentrations in Estonian aquifer systems are summarized in Table 3. Low-fluoride waters are typically found in the Cambrian–Vendian and Middle-Devonian aquifer systems (Karro et al., 2006). Fluoride concentrations in the Ordovician–Cambrian aquifer system range from 0.1 to 3.0 mg L^−1^, with relatively few wells exceeding the permissible limits (Karro et al., 2006; Veeperv & Karro, 2019; Table 3). The most significant water quality issues are observed in Silurian–Ordovician carbonaceous aquifers, where fluoride concentrations can reach up to 7.2 mg L^−1^ (Karro et al., 2009; Uppin & Karro, 2013).

Groundwater in Silurian and Ordovician carbonate rocks is mainly Ca-Mg-HCO_3_-type. Due to the high Ca content, only low amounts of fluoride are mobilized in shallow zones. Generally, groundwater pH, along with Na and Cl concentrations, increases with depth, and the water evolves toward a Na-Cl-HCO_3_ water type. Consequently, geochemically favourable conditions for high dissolved fluoride occur in the deeper parts of the Silurian–Ordovician aquifer system. Groundwater analyses show that the highest fluoride concentrations are found in wells 150–200 m deep, while the lowest values occur in shallow wells (< 30 m) drilled into the upper fractured portion of the carbonate rocks (Karro & Rosentau, 2005; Karro et al., 2006).

The Silurian–Ordovician aquifer system is comprised of various carbonate rocks, including limestones, dolomites, and marlstones with clay-rich interlayers, as well as clay-altered volcanic ash beds (K-bentonites). Leaching of these host rocks is the major natural source of fluoride in groundwater. Analyses indicate that fluorine concentrations in Estonian limestones and dolomites range between 100 and 500 mg/kg and could reach up to 1000 mg/kg in marlstones. Fluorine content correlates with the terrigenous (clay) fraction, being highest in marlstones and domerites (Uppin & Karro, 2012). K-bentonites are especially fluoride-rich, containing up to 4500 mg/kg (Haamer & Karro, 2006).

Batch dissolution tests show that fluoride concentrations in leachates are strongly correlated with the illite/illite–smectite fraction of rock samples, suggesting that clay minerals and associated ion-exchange and adsorption processes are the main sources of fluoride in groundwater. Additionally, the release of fluoride ions is controlled by the saturation state of fluorite and calcite (Uppin & Karro, 2013).

#### Poland

High fluoride content in groundwater in Poland has been well recognized in the Neogene basin “Paczków-Niemodlin,” where fluoride concentrations often exceed safe limits. Elevated fluoride has severely impacted water quality and public health, resulting in high incidences of dental fluorosis and osteoporosis. Hydrochemical studies conducted by the Polish Geological Institute–National Research Institute (NRI) helped identify and delineate the extent of the fluoride anomaly in groundwater extracted from this basin.

**Fig. 16:**
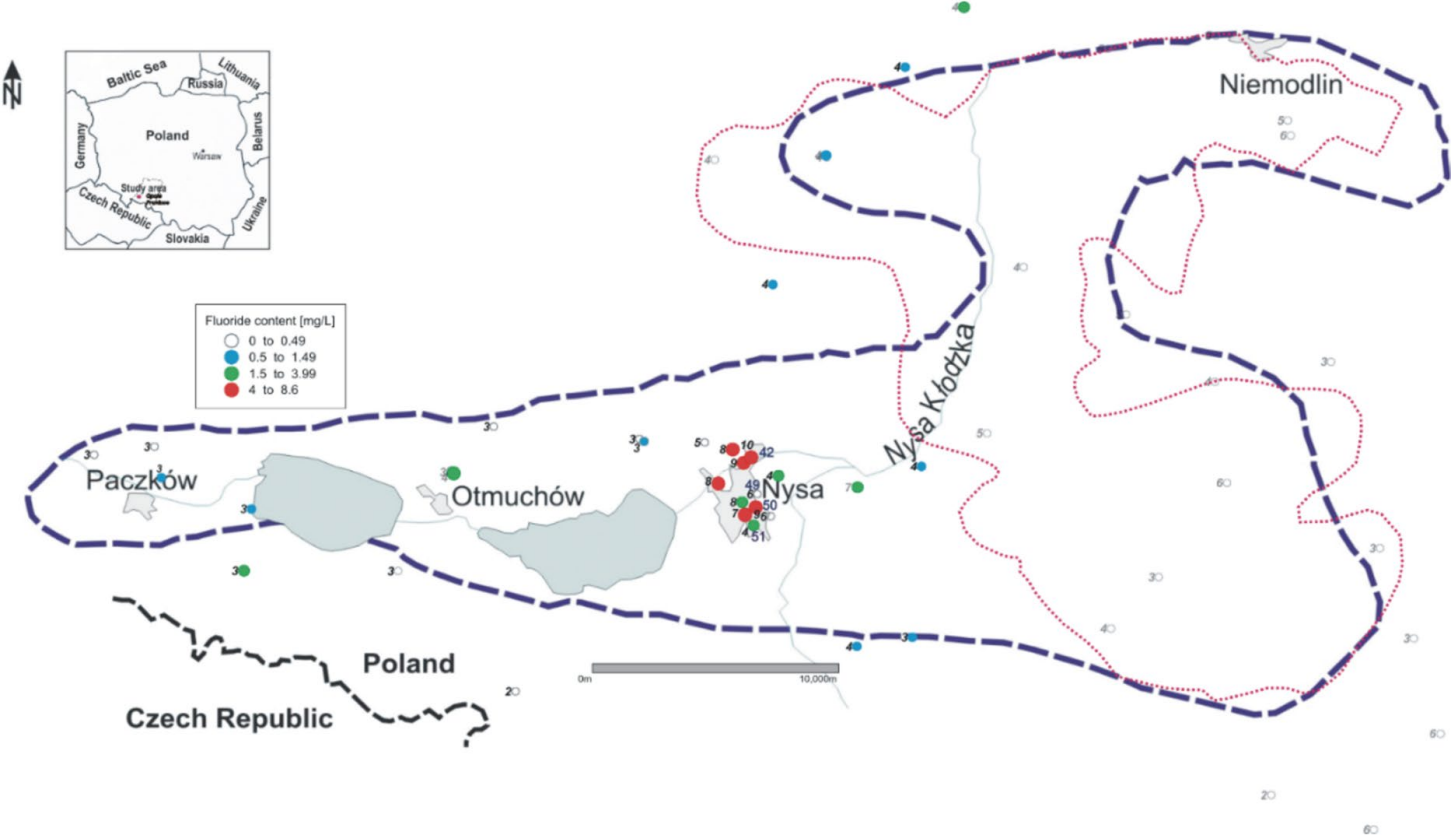
Fluoride content in groundwaters in the Neogene basin “Paczków-Niemodlin”, Poland (N = 52)

The Neogene groundwater basin “Paczków-Niemodlin,” covering an area of about 740 km^2^ in the southern part of the Opole province, is one of Poland’s main groundwater basins. Elevated fluoride concentrations were first observed during preliminary hydrogeological studies, published in 2009 (Razowska-Jaworek & Cudak, 2009), with the most comprehensive study conducted in 2012–2013 (Razowska-Jaworek & Cudak, 2022) to support aquifer protection. The major concern in the basin is groundwater contamination with fluoride, which can reach a maximum of 8.6 mg L^−1^ (Razowska-Jaworek et al., 2013), though an earlier study reported levels as high as 11.5 mg L^−1^ (Koślacz, 1989). The highest fluoride concentrations are observed in the western part of the basin, where 60% of analyses exceed 1.5 mg L^−1^ (see Table 3 and Fig. 16). In this area, fluoride levels in aquifers range from 3.0 to 6.3 mg L^−1^, with a median of 4.5 mg L^−1^ (Razowska-Jaworek & Cudak, 2022). The study also confirmed that fluoride concentrations increase with decreasing elevation of aquifers across the Neogene basin. Due to long groundwater residence times, fluoride levels remain stable, as observed over the period 1978–2019 (Fig. 16).

The primary source of high fluoride in these waters can be attributed to longer interaction with Precambrian and Paleozoic igneous and metamorphic rocks, and their weathering products, which contain fluorine-rich minerals. The presence of fluorine anomalies near large fault zones, which act as preferential pathways for deep water circulation from the Sudety Mountains, contributes to the inflow of fluoride-rich water into the Neogene aquifers (Razowska-Jaworek & Cudak, 2022).

Given the high fluoride content in groundwater (Supp. Figure S11), studies on dental fluorosis were conducted in Nysa town. Before 1997, the town’s water supply network used two sources: a surface intake on the Nysa Kłodzka River and wells from the Neogene aquifer. The effects of long-term consumption of fluoride-rich water were studied in residents of districts supplied by Neogene wells. High-intensity conditions, such as dental fluorosis and osteoporosis, were observed.

A detailed study of children aged 7–15 years in eight primary schools supplied by Neogene wells revealed significant damage to tooth enamel (Razowska-Jaworek & Cudak, 2022). Schools receiving water from wells with fluoride concentrations of 4.0–7.0 mg L^−1^ had the highest prevalence of fluorosis, ranging from 47.5 to 56.6%. In contrast, schools primarily supplied with surface water exhibited the lowest percentages of fluorosis in children.

These detailed studies enabled a relatively precise delineation of the Neogene basin “Paczków-Niemodlin.” Areas beyond the newly defined basin boundary still showed elevated fluoride in groundwater. The vertical extent of the basin was also limited by excluding lower aquifers with fluoride contamination. The high fluoride concentrations exceeding permissible drinking water limits and the resulting widespread fluorosis in Nysa led to the closure of the deepest wells in this Neogene basin.

### Latin America

#### Mexico

**Fig. 17:**
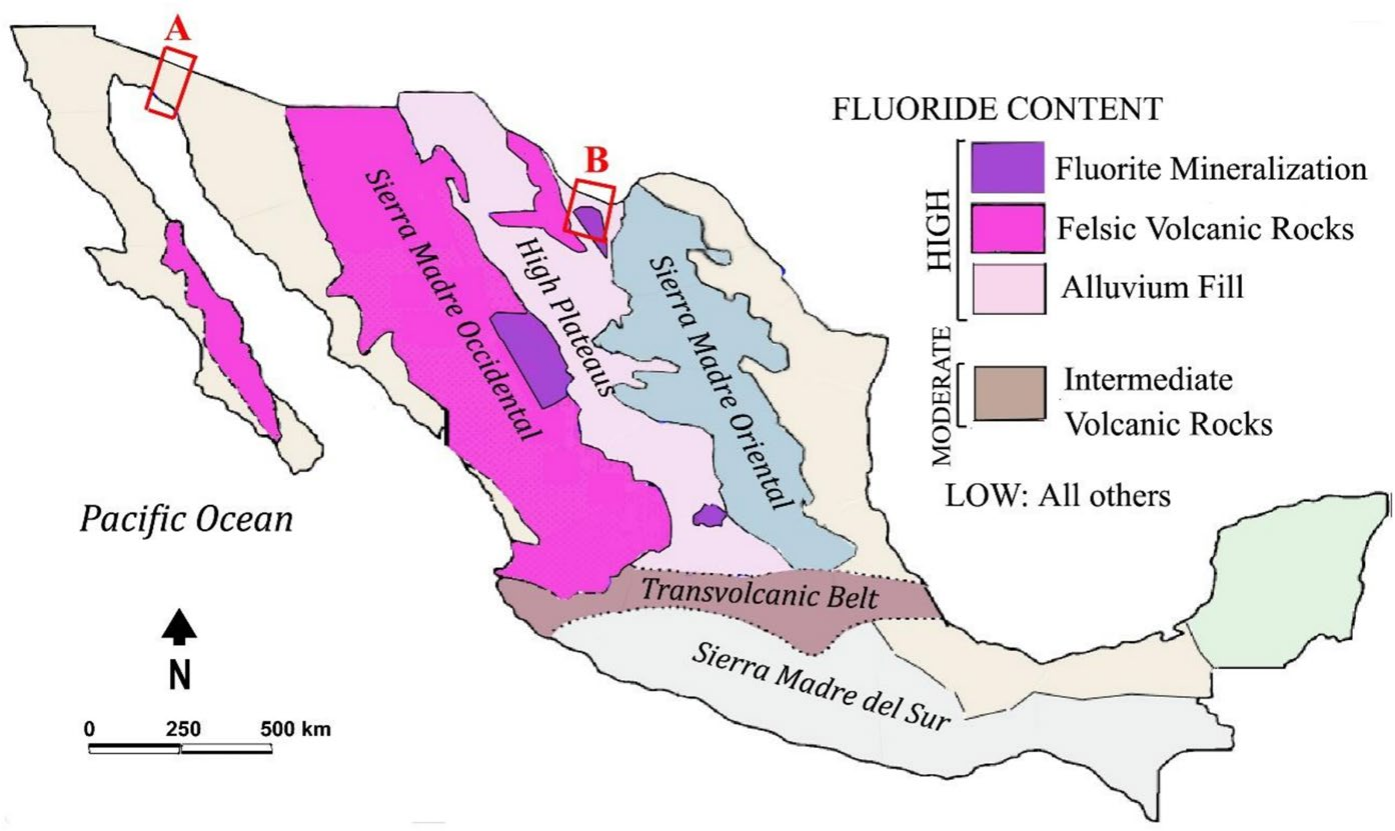
Map of Mexico showing regions of various (high, moderate, low) fluoride content and their relation to geological (type of rock) and geographical (elevation) factors. Included are the location of three main fluorite mineral deposits and two areas (boxes A and B) that require additional testing. *Note*: Sierra is a Spanish word for Mountain Range

Mexico is largely known for endemic fluorosis. Fluorosis was first reported in the mid-1900s in central Mexico following an increase in incidences of stained teeth, a common sign of the fluorosis condition. The source of fluoride was traced to the ingestion of groundwater with high fluoride concentrations (> 1.5 mg L^−1^), influenced by increased intake of fluoride-rich water (Alarcón-Herrera et al., 2001; Grimaldo et al., 1995). Once testing of fluoride in municipal drinking water wells became mandatory, the distribution and extent of fluoride concentrations became clearer (Alarcón-Herrera et al., 2020), showing wide variation across the country but concentrating in two regions: north-central Mexico and hydrothermal areas (Armienta & Segovia, 2008). The north-central region corresponds to an elevated plateau bounded by the Sierra Madre Occidental to the west and the Transvolcanic Belt in southern Mexico (see Fig. 17 and Table 3), whereas hydrothermal waters are common in the Transvolcanic Belt and surrounding areas. In contrast, low-elevation coastal areas and wells installed in limestone aquifers, such as the Yucatán Peninsula, contain little to no fluoride.

**Fig. 18:**
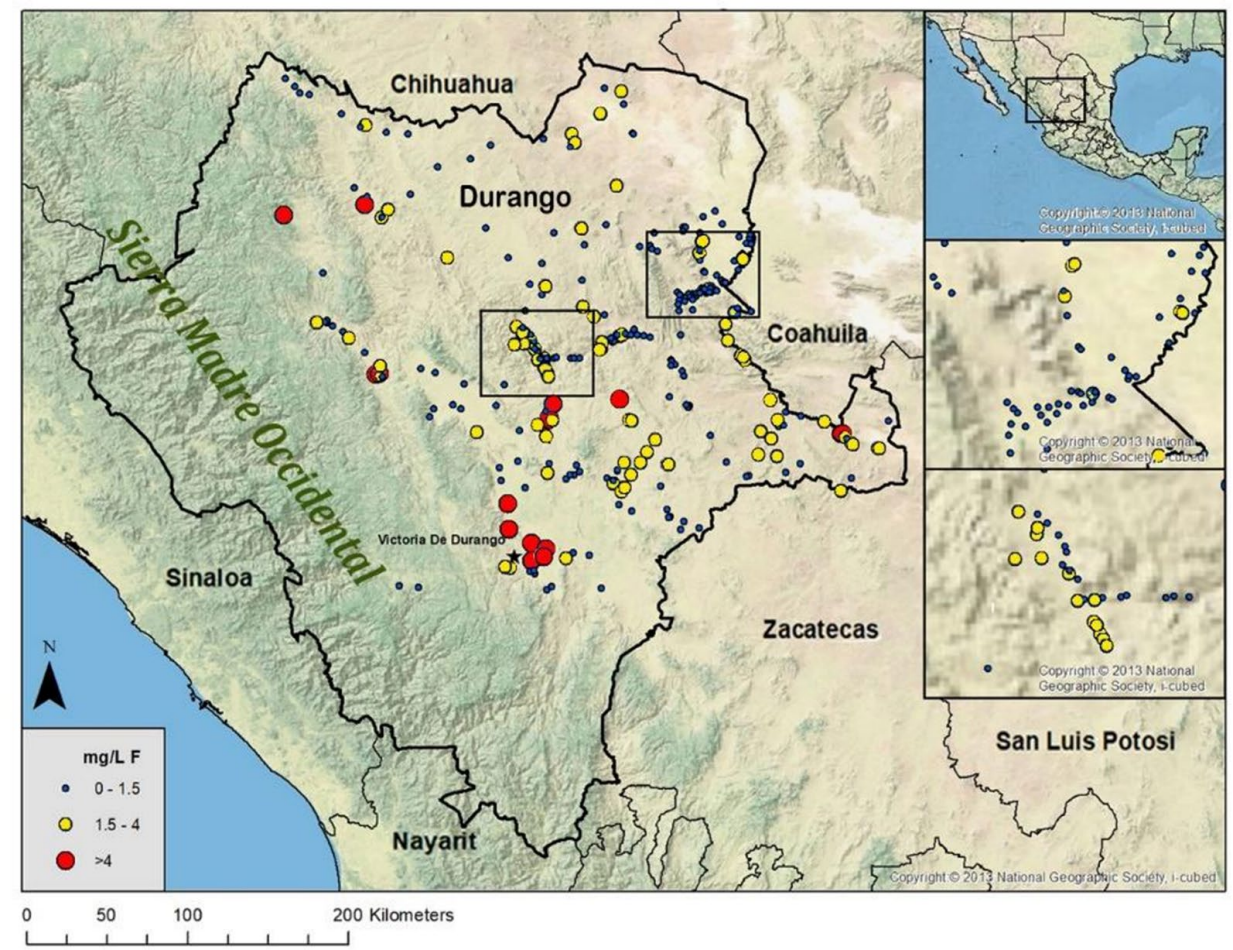
Distribution of fluoride concentrations in the state of Durango in north-central Mexico (N = 302). Different concentrations scatter within the area, pointing to alluvial fill and not the fluorite mineral deposits as their likely source

The north-central states of Chihuahua, Durango, Zacatecas, San Luis Potosí, and Aguascalientes are located on an arid to semi-arid plateau. Groundwater in this region contains fluoride concentrations between 0.03 and 27.9 mg L^−1^, with 40% of wells surpassing WHO guidelines (Gutiérrez et al., 2023; Martínez-Acuña et al., 2016; Navarro et al., 2017). Values of 3.0–4.0 mg L^−1^ are common, whereas concentrations > 4.0 mg L^−1^ are infrequent (Fig. 18). Alarcón-Herrera et al. (2020) found a positive correlation between aridity and fluoride concentration, supporting the premise that evaporation plays a pivotal role in enriching fluoride in these aquifers. In the Transvolcanic Belt bordering this plateau, fluoride concentrations reach up to 12.0 mg L^−1^, with nearly 42% of wells exceeding 1.5 mg L^−1^, where hydrothermal waters are abundant (Hurtado & Gardea-Torresdey, 2004).

Studies on the possible sources of fluoride indicate a geogenic origin associated with the weathering of felsic volcanic rocks (rhyolite, tuff, ignimbrite) that outcrop in the Sierra Madre Occidental (Fig. 17), and, to a lesser extent, with intermediate volcanic rocks in the Transvolcanic Belt. Rapid cooling of volcanic rocks can trap fluoride within the matrix rather than as discrete minerals. Additionally, hydrothermal fluids rich in fluoride crystallized after cooling, sometimes interacting with limestone host rocks, forming fluorite deposits within or surrounding the central high plateau (González-Partida et al., 2019; Fig. 17). The second major region with high fluoride corresponds to hydrothermal waters of the Transvolcanic Belt, where active volcanism allows hot mineralized water to dissolve host rocks and release fluoride and other solutes (Armienta & Segovia, 2008; Cauich-Kau et al., 2022; Hurtado & Gardea-Torresdey, 2004).

The geology of north-central Mexico is complex, but the presence of fluoride can be summarized as follows: felsic (and to a lesser extent intermediate) volcanic rocks and their fragments in alluvial fill weather as they interact with water. This region, at approximately 1,200 m above sea level, is bounded by the Sierra Madre Oriental, Sierra Madre Occidental, and the Transvolcanic Belt (Fig. 18). This setting allows for the accumulation of thick alluvial deposits in basins, forming unconfined aquifers where fluoride and other solutes, including arsenic, accumulate in the groundwater. In arid and semi-arid areas, these solutes, along with calcium and sodium ions, are further enriched by evaporation (Alarcón-Herrera et al., 2020; Gutiérrez et al., 2023). Due to heterogeneity in alluvial deposits, fluoride concentrations vary widely from well to well.

#### Argentina

Due to the known health problems of fluoride, a law regulating the provision for fluoridation or defluoridation of public water supply throughout Argentina was sanctioned in 1975 (Poder Legislativo, 1975). As per the law, the regulation sets the fluoride content in drinking water within ranges established in the Alimentary Argentine Code (AAC) (National Executive Power, 1969). In that regulation, minimum and maximum fluoride concentrations are defined, varying according to water temperature. For example, considering an average annual temperature of 21 °C, the lower limit is 0.7 mg L^−1^ and the upper limit is 1.2 mg L^−1^. A range of 0.7–1.3 mg L^−1^ can be considered applicable for most of the country. Both regulations indicate a wide variation in fluoride levels and climatic conditions across a total continental area of 2.78 million km^2^ (excluding the Antarctic claim).

**Fig. 19:**
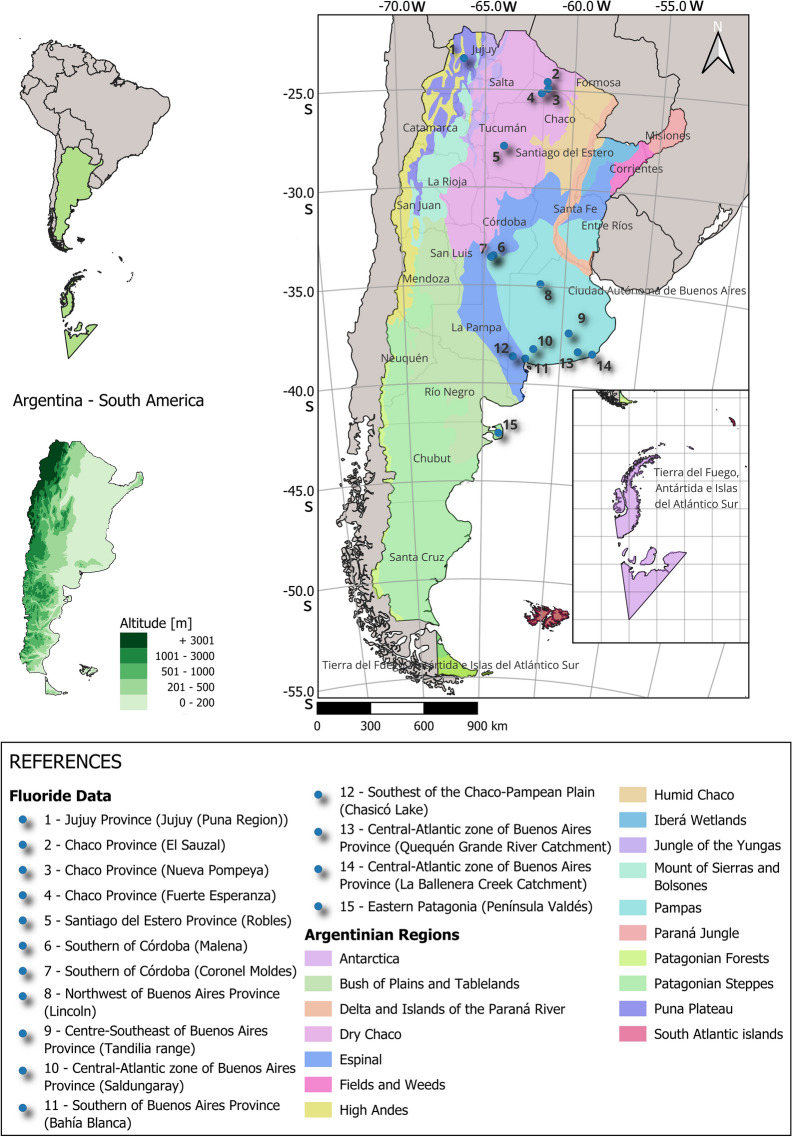
Geographical regions of Argentina and the location of the sites where fluoride contents and sources have been analyzed

Although fluoride concentrations in drinking water are widely reported, no national-scale distribution map is available. Maps at Province scale level exist, such as for Córdoba (Blarasin et al., 2014) and Tucumán (Durán et al., 2017). In southern Córdoba, groundwater from the phreatic aquifer often exceeds 2.0 mg L^−1^ of fluoride, reaching a maximum of 12.4 mg L^−1^ (Gómez et al., 2009; Blarasin et al., 2014; Fig. 19 and Table 3). In the northern and northwestern areas, fluoride concentrations fall within recommended limits. In Tucumán, most areas show fluoride below the established standard of AAC. Surface water may have lower fluoride than groundwater, as in Chaco Province (Trinelli et al., 2018), while in the Quequén Grande River catchment (Buenos Aires Province), river water and groundwater have similar fluoride concentrations (Martínez et al., 2012). Lakes may show higher fluoride due to evaporation (Puntoriero et al., 2014).

The Pampean Plain (~ 1.2 M km^2^) has been extensively studied, as it hosts a large proportion of the population and shows significant fluoride levels due to geological and geochemical conditions. In southern Córdoba, fluoride ranges from 0.5–12.0 mg L^−1^ (average 3.5 mg L^−1^, N = 40) (Gomez et al., 2009), and 1.53–8.75 mg L^−1^ (Blarasin et al., 2011). Southern Buenos Aires Province shows fluoride in groundwater: 0.4–12.7 mg L^−1^ (average 3.9 mg L^-1^) and surface water: 0.6–6.5 mg L^−1^ (average 3.6 mg L^−1^) (Espósito et al., 2013). In the Central-Atlantic zone, Quequén River catchment fluoride is 0.5–3.7 mg L^−1^ (average 2.1 mg L^−1^, N = 353) (Martínez et al., 2012), and La Ballenera Creek 1.1–2.5 mg L^−1^ (average 1.9 mg L^−1^) (Calvi et al., 2016). Inland regions reported fluoride in the range of 2.0–4.0 mg L^−1^ (Alconada-Magliano et al., 2017; Kruse & Ainchil, 2003). In the Tandilia Range, 0.44–1.68 mg L^−1^ (median 0.88 mg L^-1^) of fluoride was recorded (Barranquero et al., 2017).

Outside the Pampean Plain, fluoride levels are lower. In eastern Patagonia (Valdés Peninsula), fluoride level is in the range of 0.31–4.90 mg L^−1^ (N = 17), with ~ 23% exceeding the drinking water limit (Alvarez & Carol, 2019). In Tucumán and Santiago del Estero, ~ 50% of groundwater samples exceeded the limit (Rondano Gómez et al., 2020). In Puna, fluoride ranged from 0.20–3.80 mg L^−1^, with one anomalous 54.0 mg L^−1^; with 80% of samples exceeded the limit (Avila Carreras et al., 2008).

Volcanic ashes incorporated into sedimentary sequences are a major source of fluoride in the groundwater globally (Stewart et al., 2006). In Argentina, volcanic ashes are the main source in both the Pampean Plain (Nicolli et al., 1989) and South-Andean zone (Bia et al., 2020). Elevated fluoride is most common in wells tapping Quaternary sedimentary aquifers. The Pampean Plain is covered by loess-like volcanic sediments (~ 1.3 M km^2^) (Teruggi, 1957) rich in alumino-silicates, volcanic glass (Pye, 1995; Tricart, 1973; Zárate, 2003), and calcareous concretions or banks (Teruggi, 1957; Tricart, 1973). Southeast Córdoba loess samples showed sand and silt fractions of feldspars (40–75%) and volcanic glass (25–50%) (Nicolli et al., 1989).

Fluoride correlates with arsenic (and vanadium when measured) in many studies (Alcaine et al., 2020; Alvarez & Carol, 2019; Francisca & Carro Perez, 2009; Nicolli et al., 2012; Puntoriero et al., 2014; Zabala et al., 2016), supporting volcanic glass dissolution as the main source. Fluoride release is favoured in alkaline waters. In Puna, Andean volcanoes may contribute a separate source. Overall, high fluoride affects ~ 1 M km^2^ of Argentina, mainly in groundwater from Quaternary sedimentary aquifers formed by loess-like volcanic deposits. Maximum concentrations of fluoride reach close to ~ 12.0 mg L^−1^, about ten times the accepted limit (Espósito et al., 2013; Gómez et al., 2009).

#### Brazil

Brazil is a predominantly tropical country, and most of its population depends partially or entirely on groundwater supplies. Fluoride in drinking water has been a recognized public health issue in Brazil since 1974, when Federal Law No. 6.050/74 mandated water treatment plants to fluoridate water supplies (Presidência da República, Subchefia para Assuntos Jurídicos, 1974). The maximum allowable fluoride concentration in drinking water is 1.5 mg L^−1^, as established by the Brazilian Ministry of Health Ordinance No. 888 of 2021. The State of Rio Grande do Sul enforces a more restrictive standard, allowing water with fluoride concentrations between 0.6 and 0.9 mg L^−1^, targeting 0.8 mg L^−1^ as the ideal content (Ordinance No. 10/1999). The best risk–benefit ratio for fluoride concentration, which balances dental caries prevention and fluorosis risk, is generally between 0.5 and 0.8 mg L^−1^, depending on local climatic conditions (Adas et al., 2012; Bellé et al., 2009; Brito et al., 2016; Paulino et al., 2022).

Considering that low fluoride content does not prevent tooth decay while high concentrations cause fluorosis, studies on the optimal risk–benefit ratio are widespread in Brazil. For example, nearly 1,200 abstracts published between 2005 and 2020 by the Sociedade Brasileira de Pesquisa Odontológica (Brazilian Society of Dental Research) addressed fluorosis in cities with diverse socio-economic and environmental contexts (Soares et al., 2022). Fluoride concentrations have been assessed in small cities with fewer than 35,000 residents (Gonçalves et al., 2022; Queiroz et al., 2010) as well as in large cities approaching or exceeding 1 million residents (Bellé et al., 2009; de Moura et al., 2016; do Carmo et al., 2010; Martins et al., 2018; Modesto et al., 1999). However, studies in Brazil’s interior regions, such as the Norte (Amazon) (Gonçalves et al., 2013; Rebelo et al., 2020; Santos et al., 2002) and Centro-Oeste (Pantanal/wetland; Ferreira, 2019; Magalhães & Migliorini, 2021), are relatively rare.

**Fig. 20:**
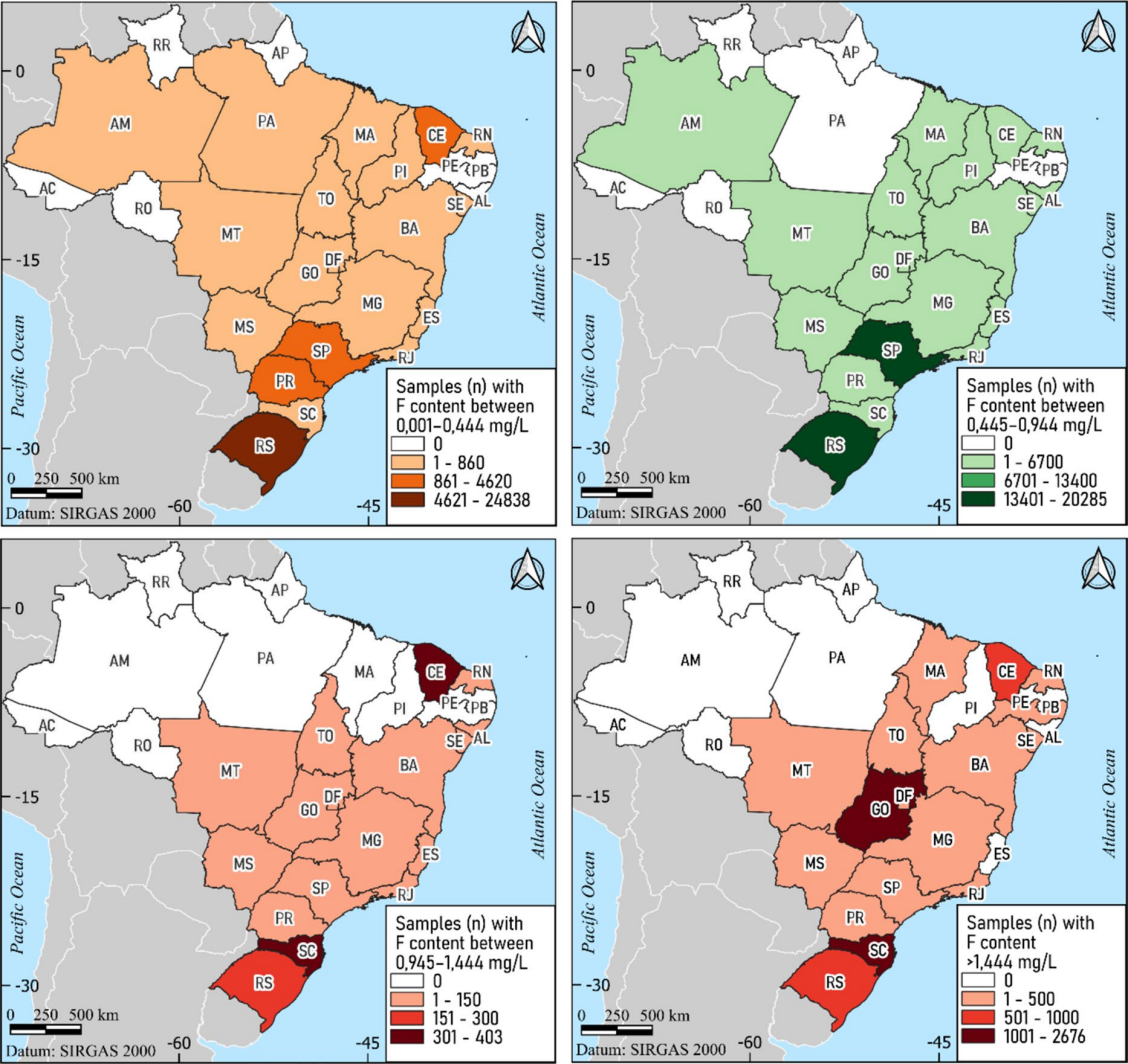
Fluoride content in water supply systems in Brazilian states (Brazilian States: AC: Acre; AL: Alagoas; AP: Amapá; AM: Amazonas; BA: Bahia; CE: Ceará; DF: Distrito Federal; ES: Espírito Santo; GO: Goiás; MA: Maranhão; MT: Mato Grosso; MS: Mato Grosso do Sul; MG: Minas Gerais; PA: Pará; PB: Paraíba; PR: Paraná; PE: Pernambuco; PI: Piauí; RJ: Rio de Janeiro; RN: Rio Grande do Norte; RS: Rio Grande do Sul; RO: Rondônia; RR: Roraima; SC: Santa Catarina; SP: São Paulo; SE: Sergipe; TO: Tocantins.). Data from SISAGUA Surveillance of Water Quality for Human Consumption.) and were filtered by Paulino et al., (2022). A total of 112,849 samples were studied

Paulino et al. (2022) evaluated fluoride content in 1,810 Brazilian cities using records from the Sistema de Informação de Vigilância da Qualidade da Água para Consumo Humano (Information System for Surveillance of Water Quality for Human Consumption). The database included 112,849 records, of which 7,359 showed high fluoride content (0.945–1.44 mg L^−1^) and 1,502 exceeded 1.44 mg L^−1^. States with the most records of very high fluoride were Santa Catarina (N = 403), Goiás (N = 320), Rio Grande do Sul (N = 284), and Ceará (N = 153) (see Fig. 20 and Table 3). In Ceará, Almeida (2022) analysed 30 groundwater samples from nine cities, finding no sample exceeding 0.7 mg L^−1^ and 56.7% (N = 17) below 0.5 mg L^−1^, including treated water.

Interestingly, Almeida (2022) and Paulino et al. (2022) studied the same state (Ceará, population ~ 9 million) but reported different results, illustrating the high spatial variability of fluoride concentrations. Lima et al. (2021) observed similar variability in Paraíba State. In a study of 571 groundwater samples collected by FUNASA (Brazilian National Health Foundation), Lima (2020) reported 51 samples with fluoride concentrations between 1.0 and 1.5 mg L^−1^ and 68 samples above 1.5 mg L^−1^, distributed across 28 towns. The highest concentration (9.0 mg L^−1^) was detected in São José do Rio do Peixe, a town underlain by metagranitoid biotite and amphibolite bedrock. This area is reported to be endemic for dental fluorosis (Lima, 2020).

**Fig. 21:**
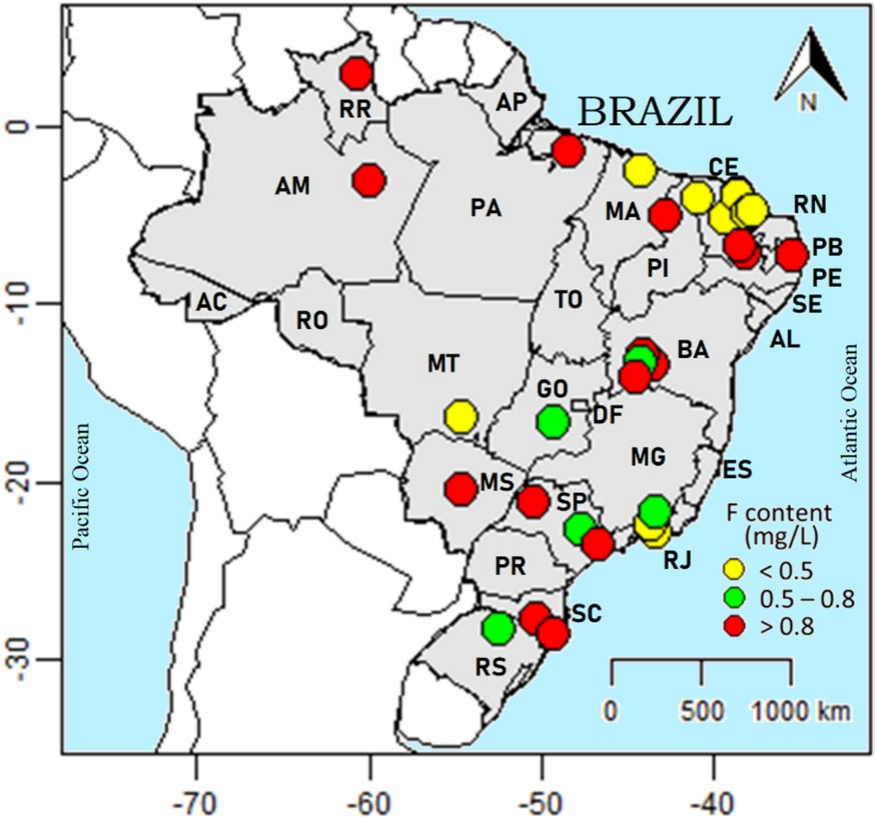
Spatial representation of fluoride content in drinking natural and treated water in Brazil. Each point represents a town. Considerable variation in fluoride content is observed in geographically close towns. Coastal states have more data available than inland states. Data from the authors discussed in this text and not from SISAGUA

Such substantial regional and local variability, as well as differences between groundwater and surface water, highlights the complexity of fluoride distribution in Brazil (Fig. 20). A literature review identified 16 studies analysing fluoride content in Brazilian cities, confirming high spatial variation (Fig. 21). Brazil’s geological and climatic diversity makes nationwide assessments challenging; local environmental characteristics must be considered when evaluating fluoride risk. Frazão et al. (2011) provide recommended fluoride concentrations for each of the 26 Brazilian state capitals and the Federal District, accounting for maximum average temperatures.

In western Bahia State, Neoproterozoic pelito-carbonate rocks of the Bambuí Group overlie an Archaean–Paleoproterozoic gneissic-migmatitic basement (Misi et al., 2007). Groundwater from this region, intended for human consumption, has been found to exceed 0.8 mg L^−1^ and 1.5 mg L^−1^ of fluoride in some areas (Coutinho, 2014; Gonçalves et al., 2022), putting the population at higher risk of fluorosis. Water exceeding 1.5 mg L^−1^ requires treatment before human consumption.

Kath et al. (2015) analysed groundwater in a granite basement mine cut by acidic Eo-Paleozoic subvolcanic dikes and the Cocal fluorite vein. Fluoride concentrations at 300 m and 150 m depths were 7.3 and 1.67 mg L^−1^, respectively. The highest concentration (14.29 mg L^−1^) was recorded in tailings dam water. Treated drinking water supplied by the local company contained only 0.42 mg L^−1^. These findings highlight the need for careful management of deep and mining-related waters to prevent high fluoride exposure via surface drainage.

São Paulo State, the most populous in Brazil with 44 million residents, relies on groundwater for 80% of city water supply. Natural groundwater often exceeds 1.5 mg L^−1^ fluoride, contributing to dental fluorosis (Adas et al., 2012; Catani et al., 2007; Martins et al., 2018). The state’s geological diversity leads to varied fluoride sources, including weathering and remobilization of fluorine precipitated from ancient hydrothermal fluids, particularly during the Eocene (Martins et al., 2018).

#### Colombia

Colombia is a country located in South America, bordered by the Caribbean Sea and Pacific Ocean, and by Venezuela, Brazil, Peru, and Ecuador. The geology of areas with high fluoride concentrations varies considerably but is generally associated with sedimentary and volcanic rocks. For example, in the Department of Boyacá, sedimentary rocks such as sandstones, shales, and marls dominate, whereas in the Department of Nariño, volcanic rocks like andesites and basalts are prevalent. These areas are often located in regions of high tectonic activity, which can substantially contribute to the release of fluoride into water and soils (Escalante, 1990; Gobernación del Cauca, 2019). For instance, in the Department of Huila, the Neiva geological fault is believed to be responsible for fluoride release into groundwater (see Table 3 and Supp. Figure S12).

It is important to note that geology is not the sole factor influencing high fluoride concentrations in water and food. Several other sources include agricultural and mining activities, industrial discharges, and natural sources such as deposits of fluorine-containing minerals like fluorspar or cryolite. Additionally, volcanic emissions and weathering of fluorine-rich rocks may also elevate fluoride levels in the environment (Revelo-Mejía et al., 2023).

A study by Misnaza Castrillón et al. (2021) characterized Colombia in terms of drinking water sources and dental fluorosis rates. Using data from the Subsistema de Información de Vigilancia de Calidad de Agua Potable (SIVICAP) and the Sistema de Vigilancia en Salud Pública (Sivigila) for the period 2012–2018, the study confirmed that the Andean region is most at risk of elevated fluoride concentrations (Alvarez & Carol, 2019). In arid regions, groundwater is often oxidizing with neutral to alkaline pH, favouring silica dissolution and the release of anions such as fluoride. However, despite localized high-fluoride areas, Colombia overall cannot be considered a fluorosis-endemic country.

In a study by López-Salgado et al. (2016), fluoride content in water from the aqueduct of the Proactiva company in Los Garzones (Montería) ranged from 0.04 to 0.07 mg L^−1^, which is notable considering that the company performs fluoridation. Similarly, Revelo-Mejía et al. (2022) reported fluoride concentrations in surface water in the Cauca region below 0.83 mg L^−1^, even in areas near the Puracé volcano.

Another study by Revelo-Mejía et al. (2021) measured fluoride in 149 water samples from various aqueducts in Cauca. Fluoride concentrations were found to be below 0.3 mg L^−1^. Urban areas, such as Santander de Quilichao, had lower fluoride levels (0.027–0.068 mg L^−1^) compared to rural areas, while the municipality of Cajibío recorded 0.082–0.186 mg L^−1^. The highest levels were observed in Timbío (0.12–0.21 mg L^−1^). These values are insufficient to trigger dental fluorosis or to provide protection against dental caries.

Overall, these data suggest that surface water sources (rivers, aqueducts, lakes) generally contain lower fluoride concentrations than groundwater sources, such as water galleries. Fluoride contamination is localized in certain regions of Colombia and does not affect the entire country.

### United States of America

**Fig. 22:**
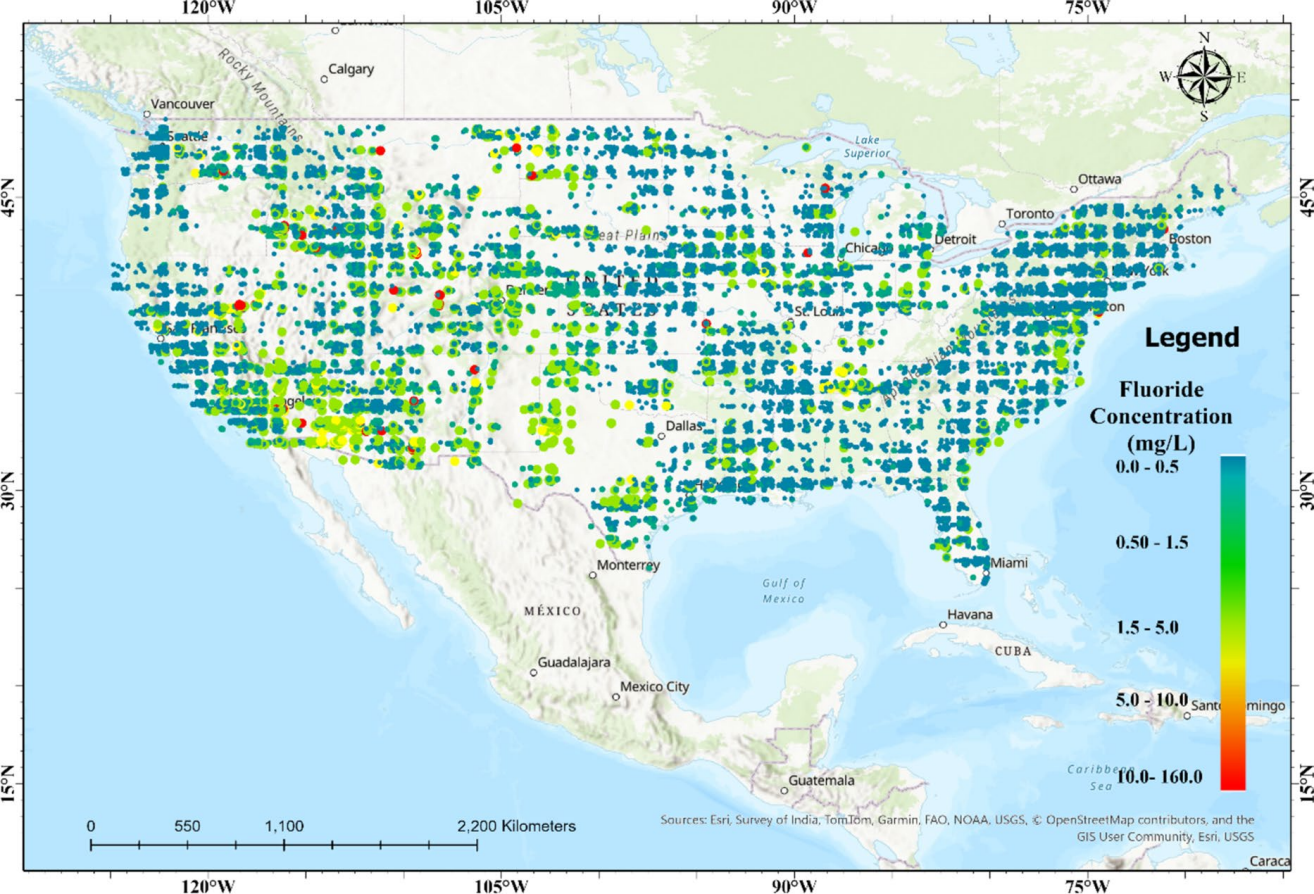
Fluoride concentrations in the groundwater of USA (data retrieved from USGS; https://www.usgs.gov/) (N = 38,105)

In the United States of America (USA), the U.S. Department of Public Health Service (2015) recommends 0.7 mg L^−1^ as the optimal level of fluoride in drinking water. A detailed investigation of over 38,000 wells found that 10.9% of wells exceeded this optimal limit (McMahon et al., 2020). The study showed that fluoride levels were generally higher in the western part of the country compared to the eastern part, and in many cases exceeded the WHO limit (see Fig. 22 and Table 3).

Some localities in the central-eastern areas also exhibited fluoride concentrations above the recommended limit, likely due to Ordovician carbonate rock deposits (McMahon et al., 2020). McMahon et al. (2020) noted that groundwater in crystalline rock regions tends to have lower fluoride concentrations, whereas in western USA, crystalline rocks could contribute to elevated fluoride. Deeper wells were reported to have higher fluoride concentrations than shallow wells, highlighting that fluoride solubility increases with temperature at greater depths (Edmunds & Smedley, 2013; Zhang et al., 2017).

A national assessment of 25 aquifers indicated elevated fluoride levels under favourable geochemical conditions, such as Na-HCO_3_ water, alkaline pH, increased salinity, and calcite precipitation (Belitz et al., 2022). Two potential aquifers in humid climates posed a risk of elevated fluoride exposure to more than half of the population.

Long-term assessment of groundwater in Texas (Chaudhuri & Ale, 2014) revealed that high fluoride in shallow aquifers, particularly in western Texas, was attributed to agricultural practices. In other parts of Texas, high fluoride in deeper aquifers was likely due to prolonged water–rock interaction and mineral dissolution. Analysis of Texas groundwater from 1929 to 2019 indicated that 19% of samples exceeded 2.0 mg L^−1^ fluoride, and 4.6% exceeded 4.0 mg L^−1^ (Reedy & Scanlon, 2020). Semi-arid climates with low flow and limited flushing contributed to elevated fluoride levels.

Harkness and Jurgens (2022) observed that fluoride concentrations in the highly complex hydrogeological regions of California are primarily controlled by hydrogeology, climate, and groundwater chemistry. Rosecrans et al. (2022) applied random forest models to predict fluoride concentrations in untreated groundwater in parts of the USA. The model performed well at the regional scale and was useful in identifying areas vulnerable to elevated fluoride in unmonitored regions.

### Australia

Concentration of fluoride in public drinking water in Australia is required to be below the Australia Drinking Water Guidelines maximum permissible limit of 1.5 mg L^−1^ (NHMRC, 2011). Monitoring of fluoride in drinking water sources is conducted mainly by state government entities and analytical results are available online (https://www.watercorporation.com.au/About-us/Our-performance/Drinking-water-quality). Elevated fluoride in drinking water sources is not a major problem for major drinking water supplies in Australia. This may be mainly due to the fact that most of the drinking water sources for the major population centres such as Sydney, Melbourne, Brisbane, and Adelaide are surface water based (COA, 2001). The exception is Perth which currently sources approximately 100 GL/a of groundwater from the Superficial, Leederville, and Yarragadee aquifers of the Perth Basin as part of an integrated public water supply which also includes desalination, surface water, and groundwater replenishment sources (Davidson, 1995; https://www.watercorporation.com.au/Our-water). Groundwater from the Perth Basin is, however, naturally low in fluoride and fluoridation of Perth’s drinking water supply is undertaken to achieve an optimal range of 0.7 to 0.9 mg L^−1^ with a maximum permissible concentration of 1.0 mg L^−1^ (https://www.watercorporation.com.au).

Podgorski and Berg (2022) present the risk of fluoride in groundwater around the globe including Australia based on hydrogeological and other related factors (see Supp. Figure S13). This mapping shows that areas of central Australia which are sparsely populated have the highest likelihood of elevated fluoride. Ransley et al., (2015) present the distribution of fluoride in the Cadna-owie/Hooray Aquifer, which is the shallowest and most-exploited aquifer of the Great Artesian Basin of Australia (see Supp. Figure S14 and Table 3). While the Great Artesian Basin is generally only used for small drinking water supplies, the interpolated fluoride distribution (N = 4883) shows extensive areas of elevated fluoride (> 1.5 mg L^−1^) within the Cadna-owie/Hooray Aquifer (Ransley et al., 2015). High fluoride concentrations in the Cadna-owie/Hooray Aquifer have been attributed to groundwater being in contact with igneous (hydrogeological basement) rocks or associated with fine-grained sedimentary rocks. A likely primary mineralogical source for dissolved fluoride is ubiquitous trace fluorapatite in igneous rocks or trace carbonate-rich fluorapatite in fine-grained sedimentary rocks (Schafer, 2020). Primary fluoride may also be derived from mica group minerals (e.g. biotite) (Ransley et al., 2015; Table 3).

## Defluoridation techniques adopted in the studied countries

Several defluoridation methods, such as membrane separation, adsorption, ion-exchange, chemical coagulation–precipitation, and electrocoagulation, have been devised to maintain fluoride within the permissible limit globally at the laboratory scale (Table 4; He et al., 2020a, 2020b; Kumar et al., 2022a, 2024; Lacson et al., 2021). For instance, fluoride adsorption on activated charcoal and activated alumina, ion-exchange using resins, precipitation of fluoride with aluminum salts (Nalgonda process), reverse osmosis, electrodialysis, and nanofiltration are a few commonly applied defluoridation techniques (Addar et al., 2024; Kumar et al., 2023). These methods can be implemented from household to community scale, primarily for decentralized water supply systems, depending on the target population and the chosen treatment method. However, electrodialysis, reverse osmosis, and nanofiltration are sophisticated approaches that require advanced technology and high costs, making them unsuitable for community-level application in developing countries. In rural areas lacking piped water supply, community-based defluoridation interventions remain a promising option. Yet, challenges often arise from limited economic support, sustaining the continuous efficiency of defluoridation units, insufficient long-term community participation, and the disposal or regeneration of fluoride-rich sludge (Table 4).

In India, phosphoric acid–crushed limestone treatment (PACLT), a practical, affordable, safe, and environmentally friendly technique for fluoride treatment, namely “Fluoride Nilogon” was developed by researchers from Northeast Assam (Gogoi et al., 2015; Mohan et al., 2020). In this method, fluoride is removed through the precipitation of fluorite, fluorapatite, and physisorption by hydroxyapatite generated in situ from the reaction between calcium and phosphate ions in the reactor in plug-flow (batch) mode (Gogoi et al., 2015). In certain fluoride-affected regions of the East Karbi Anglong district, the PACLT approach was field-tested on a modest scale at the community and household levels. Without necessitating any interventions, such as limestone replacement or substitution over five and a half years, the field units have been operating consistently. A domestic Fluoride Nilogon unit’s crushed limestone bed is expected to last more than 50 years when used twice daily with feed water containing 20 mg L^−1^ fluoride. With a larger dose of phosphoric acid, fluoride can be completely removed regardless of baseline concentrations. The field study demonstrates the efficacy of Fluoride Nilogon as an effective fluoride removal technique in rural areas (Mohan et al., 2020). Some state governments, such as the government of Andhra Pradesh, have initiated pilot projects to study the use of biochar for fluoride removal from drinking water (Mukate et al., 2022).

Practical and low-cost, nature-based methods should be explored, and the implications of managed aquifer recharge (MAR) as a measure to dilute fluoride in groundwater have not yet been widely adopted. Rainwater harvesting for augmenting groundwater recharge is becoming mandatory in many countries and may additionally dilute high-fluoride groundwater (Brindha et al., 2011). However, the success of this approach depends on the initial fluoride concentrations and the volume of water recharged. Therefore, detailed scientific studies are required to assess MAR applicability in the long term (Brindha et al., 2016; Kalpana et al., 2019).

In China, defluoridation techniques such as activated alumina adsorption, reverse osmosis, and coagulation/flocculation have been employed to remove fluoride from drinking water (Wang et al., 2019, 2021). Although effective, these treatment methods are costly and require advanced infrastructure (Li et al., 2001). Long-term solutions should focus on source control and the prevention of contamination, including regulating industrial discharges, minimal use of fluoride-containing fertilizers and pesticides, and enforcing stringent policies for regular water quality monitoring and proper waste management practices (Li et al., 2001; Wang et al., 2023a, 2023b; Yang et al., 2025).

Studies testing local indigenous defluoridation methods have shown them to be adaptable and affordable in Nigeria. For instance, Ohwofasa et al. (2017) tested fluoride removal using clays, Oladoja et al. (2019) used lateritic soils, Ekine et al. (2019) used locally produced adsorbents from eggshells, and Jagaba et al. (2019) used activated carbon from *Tridax procumbens* for effective defluoridation at low cost. Earlier, Erhuanga et al. (2014) formulated a ceramic filtration setup integrating clays, bone char, laterite, and charcoal for defluoridation. These studies in Nigeria have shown positive outcomes for significant fluoride removal from groundwater. Nevertheless, cost-effectiveness determines whether low-income inhabitants of vulnerable regions can easily adopt them. Additionally, where defluoridation techniques cannot be easily implemented, researchers have advised providing alternative safe sources of drinking water (Giwa et al., 2021; Ibiyemi, 2020).

Defluoridation in Ghana is still in the initial phase due to limited work carried out specifically for the regional environment. Despite this, adsorbent materials such as bone char, aluminum-coated bauxite, and limestone have been used for groundwater defluoridation (Droepenu, 2016; Fosu et al., 2025; Kumi et al., 2023; Salifu et al., 2016). For example, Kumi et al. (2023) employed activated bone char to reduce highly fluoridated water in the North East Region of Ghana. They explained that increasing the dosage of bone char and the contact time with groundwater enhances the efficacy of the defluoridation technique. Precipitation techniques using activated alumina with indigenous laterite and bauxite have also been employed (Craig et al., 2015b). Like other developing countries, advanced technologies such as reverse osmosis and ion exchange cannot be afforded by all. Remarkably, the Ghana Water Company Limited uses advanced technologies like reverse osmosis and ion exchange to treat water for urban areas. However, challenges remain in rural areas, where a large population depends on limited surface water sources, hand-dug wells, and boreholes provided by the Government of Ghana through partnerships with international organizations. Defluoridation of contaminated groundwater is not being performed in fluorosis-endemic parts of rural Ghana, as residents cannot afford costly technologies.

Water treatment facilities in Ethiopia supply potable water to residents in cities and towns. Owing to reported high fluoride levels, several point-of-use water treatment technologies have been implemented. For example, common community-based treatment systems include the use of activated alumina, bone char, Nalgonda technique, contact precipitation, and reverse osmosis (Mosler et al., 2011; Osterwalder et al., 2014). Interestingly, many residents do not use these treatment methods due to maintenance costs, cultural or religious beliefs, leaving some installed units underutilized (Kloos & Tekle Haimanot, 1999; Osterwalder et al., 2014; Tekle-Haimanot et al., 2006).

In Kenya, established defluoridation programs include the Catholic Diocese of Nakuru Defluoridation program, which provides household bucket-size and community-level defluoridation plants. The program uses the bone-char method for fluoride removal. Despite its availability, the program needs better outreach to cover all affected communities. For example, 75% of the population in Nakuru town were unaware of the program, and only 19% of the sampled population actually used the defluoridation filters (Ochieng, 2013). No other defluoridation programs have been reported in Kenya. However, several experimental studies have shown the potential of locally available materials, such as alum and wood ash leachate, to remove up to 99% of fluoride from water (Kazungu et al., 2022). However, these methods have not yet been tested at household or community scale. To achieve a national low-fluoride drinking water status, a multidisciplinary, solution-focused research approach is needed. First, a detailed geochemical database of all groundwater resources should be established to identify areas where fluoride exceeds recommended limits. Second, through public education programs, local authorities should inform communities about dietary and water-use practices to prevent or reduce the effects of high fluoride exposure (Mwaniki et al., 1994; Ndambiri & Rotich, 2018). Currently, new research on effective defluoridation methods suitable for rural regions, where most high-fluoride groundwater occurs, is limited. Public awareness of the health effects of fluoride also needs to be strengthened, particularly in rural communities, to increase sensitivity and urgency for mitigation measures (Gevera et al., 2022b).

In Kenya, several lessons have been learned regarding defluoridation programs. The RANAS (Risk, Attitude, Norm, Ability, and Self-regulation) model shows that awareness alone does not guarantee the adoption of safe water habits (Nocella et al., 2022). This was attributed to a lack of trust in foreign companies due to hidden maintenance costs associated with defluoridation programs (Nocella et al., 2022). Other challenges of defluoridation in Kenya include recurring maintenance costs and misconceptions about high-fluoride water due to its lack of odour and colour (Gevera et al., 2019; Nocella et al., 2022). Another observation was that factors such as solidarity and norms, as well as management logistics such as electricity costs, led most people to prefer community rather than household defluoridation methods (Nocella et al., 2022). However, visual nudges, such as coloured signposts at community water points, can help communities adopt healthy practices (Nocella et al., 2022). Table 5 summarizes barriers and potential remedies that defluoridation programs are facing in Kenya.

**Table 5:** Lessons learned on barriers and potential remedies on defluoridation in Kenya

Category	Key lessons and findings		References
Barriers to defluoridation	**Awareness & beliefs**: High awareness of “brown teeth” exists, but cause-and-effect is misunderstood. Many residents believe salty water causes fluorosis or rely on ineffective traditional remedies like brushing with herbs or soil and drinking milk to "whiten" teeth **Maintenance costs**: The “hidden costs” of replacing sorbent media (such as bone char or pellets) are the primary reason for program failure. Many households can afford the initial filter but not the recurring expense of media replacement **Taste & odor**: New bone char filters often produce water with initial turbidity, color, and a specific odor, which can lead to early abandonment if not flushed properly. Conversely, untreated “isokot” (salty) water is often perceived as safe because it looks clean **Trust**: A significant lack of trust toward foreign companies exists because they often introduce technologies without disclosing long-term maintenance requirements		Gevera et al., (2022b), Nocella et al. (2022), Otieno et al., (2024), Catholic Diocese of Nakuru (CDN) and Muller (2006), Catholic Diocese of Nakuru (CDN) (2009), Resilience (2024), Kenya Dental Association FDI World Dental Federation (2023), Korir et al., (2009)
Potential remedies	**Community financing:** Programs are moving toward installment-based payment plans rather than lump sums. Innovative funding like carbon credits and government tax exemptions on defluoridation equipment are recommended to lower costs **School-based programs:** Because dental fluorosis is permanent if exposure occurs before age seven, installing machines in mandatory schools is a high-priority remedy to protect the most vulnerable demographic **Periodic media replacement:** Success requires regular monitoring by the provider to identify when the media is saturated, as fluoride is invisible and tasteless. Innovations like calcium-phosphate pellets and contact precipitation can extend the filter's lifespan by 3 to 6 times, reducing the frequency of replacement **Social solidarity:** Most participants prefer community-level filters over household units because they ensure neighbors aren’t left behind and can be centrally managed		

In Tanzania, Kimambo (2023) suggested a variety of locally available raw adsorbents, such as gypsum, bauxite, and magnesite, for defluoridation to combat fluorosis. Kimambo et al. (2023) highlighted calcined bauxite as a particularly cost-effective method compared to other adsorbents. Gutierrez et al. (2023) emphasized the need for serious policy interventions, noting that existing policies and limited government funding are insufficient to protect rural populations from fluorosis.

In Yemen, no practical measures have yet been implemented to prevent or minimize fluoride contamination in affected areas. Only recommendations exist, such as using alternative water sources, improving the nutrition of high-risk populations, and adopting defluoridation methods including adsorption, ion exchange, precipitation–coagulation, membrane separation, electrolytic defluoridation, and electro-dialysis (Ahmad et al., 2022). In urban areas, many households rely on reverse osmosis systems for their drinking water and cooking needs.

In Estonia, reverse osmosis technology and the development of new wells have been introduced to reduce fluoride concentrations. These measures have substantially decreased overall fluoride exposure (Indermitte et al., 2014). Geochemical studies indicate that local geology must be considered when developing new water supply wells. Opening clayey carbonate layers with well screens should be avoided to ensure safe fluoride levels.

In the Nysa region of Poland, measures for clean and safe water supply include: (i) closing and abandoning wells with very high fluoride contents, (ii) treating wells with fluoride concentrations of around 1.5–3.0 mg L^−1^, using sand and gravel filters and aeration, and (iii) observing that shallow wells used by local residents generally do not exceed fluoride limits, though some households used home water purifiers. In areas with high fluoride levels, over 25% of the population is affected by fluorosis (Razowska-Jaworek & Cudak, 2022).

In Argentina, a sustainable solution for fluoride contamination has not yet been achieved, and documenting fluoride distribution in the aquifer remains challenging. This is likely due to the large heterogeneity of loess-like deposits, which have been reworked by fluvial transport in many areas. Regions affected by high fluoride concentrations, often accompanied by elevated arsenic levels, are addressed through structural and non-structural solutions (Quinodoz et al., 2019). Among structural solutions, supplying water from external catchments, typically via aqueducts from rivers located hundreds of kilometres away, is the most commonly used approach. Where feasible, deeper wells accessing confined aquifers with lower contaminant levels are drilled. Non-structural solutions include the use of bottled water. Unfortunately, for small towns or rural populations, acceptance of the risk remains a frequent solution (Becerra et al., 2020).

In Colombia, reverse osmosis has been installed as an effective method to reduce fluoride in the water supply (Alarcón-Herrera et al., 2013). Maintenance and installation costs for such plants have decreased, making them feasible in regions most affected by fluoride. A study by Serrano-Florez et al. (2023) explores the use of renewable energy to desalinate water and provide energy to isolated communities. Their study uses mathematical models to simulate a reverse osmosis system applied to brackish water sources in La Guajira, Colombia. The region hosts indigenous communities at high and medium altitudes with brackish underground wells, which could provide potable water and improve quality of life. Additionally, La Guajira’s high solar radiation potential suggests that solar-powered desalination could be an efficient solution for water supply in these isolated communities (Serrano-Florez et al., 2023).

In Australia, high-fluoride groundwater in small rural and remote communities is typically treated using reverse osmosis desalination plants (https://www.sawater.com.au). Other state water authority methods include activated alumina treatment or blending water from multiple bores to remain below the maximum guideline limit for fluoride. In a managed aquifer recharge scheme in the Perth Basin, peaks in fluoride concentration were observed during the injection of deionized water into a siliciclastic aquifer (Sun et al., 2020). Post-injection increases in fluoride were associated with reduced calcium, increased sodium and bicarbonate, and higher pH. Experimental and modelling studies suggest that this elevated fluoride arises from a charge-balanced exchange, with H^+^ replacing Ca^2+^ and fluoride on the surface of carbonate-rich fluorapatite (Chaïrat et al., 2007a, 2007b; Schafer et al., 2018, 2020, 2021). This mechanism is plausible given the widespread presence of trace fluorapatite minerals in rocks worldwide, which may explain many instances of elevated fluoride not linked to fluorite dissolution. Further research and validation are needed to confirm this mechanism.

**Fig. 23:**
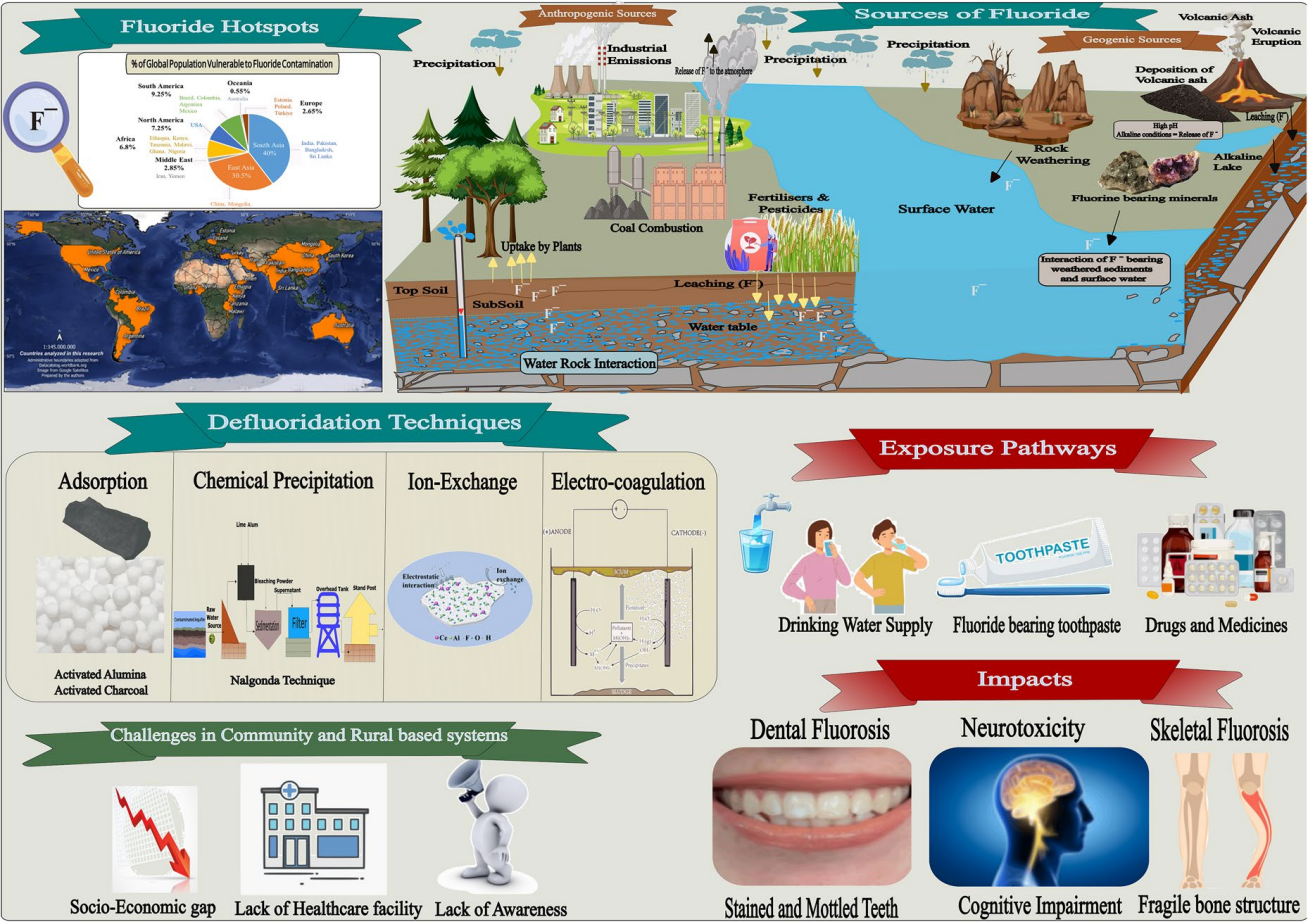
Conceptual model illustrating fluoride hotspots, sources, exposure pathways, defluoridation strategies, challenges, and public health impacts

Finally, Fig. 23 presents a conceptual model, based on the above findings, illustrating fluoride hotspots, sources, exposure pathways, defluoridation strategies, associated challenges, and public health impacts.

## Social aspects and policy interventions

Scientific articles often do not emphasize social and policy interventions. Here, we document these aspects in detail, considering the targeted countries.

Rahman et al. (2020) highlighted the necessity for a major research program in Bangladesh to evaluate clinical evidence and therapeutic alternatives. They raised awareness of groundwater fluoride concentrations and associated health risks in coastal Bangladesh. Low-cost defluoridation methods could provide safe water to large coastal communities. A large-scale investigation based on clinical evidence of fluoride exposure from drinking water, the availability of inexpensive fluoride tests, and increased understanding of water quality and health could reduce health concerns in Bangladesh. Most coastal inhabitants are highly vulnerable to extreme climatic events and are economically disadvantaged. This limits their access to modern technologies like reverse osmosis. Currently, there is no policy intervention ensuring fluoride-free water across the country.

In India, many unlicensed drinking wells are privately owned due to frequent water supply interruptions caused by inadequate infrastructure or unmanaged public water systems. Consequently, people rely on their private wells for drinking water, often unaware of fluoride contamination. Municipal water supply is often inadequately filtered and requires filtration for safe consumption. Further research and pilot projects are needed to develop policies and regulations for implementing low-cost and effective adsorbents, such as biochar, for water treatment (Boraah et al., 2022; Quispe et al., 2022). Proper procedures for the safe disposal and management of post-treatment waste are also critical.

Considering the severity of fluorosis, the National Programme for Prevention and Control of Fluorosis (NPPCF; https://cghealth.nic.in/) was initiated by the Ministry of Health and Family Welfare, Government of India, in 2008–2009. The “Fluoride Contamination (Prevention) Bill, 2017” was introduced in the Indian Parliament to provide regulations for national policy formulation on mitigating and preventing fluoride contamination in food and drinking water. Implementation has occurred in Kamrup (metro), Nagaon, and Karbi Anglong districts of Assam, with expansion to other affected northeastern districts pending (National Programme for Prevention & Control of Fluorosis, Directorate of Health Service, Government of Assam). Shah and Indu highlighted that in India, more emphasis is placed on communicable diseases than on irreversible fluorosis issues (https://inremfoundation.org/).

In Pakistan, community water filtration systems, including activated carbon tanks and cartridge filters, are often poorly monitored and regulated. Those who can afford it install household-based filtration units such as cartridge, ultraviolet, or reverse osmosis systems. Limited research, inadequate policy, and poor infrastructure hinder the effective management of contaminated groundwater. Only limited households above the poverty line can afford advanced filtration technologies.

In Mongolia, many drinking water wells, particularly in the steppe and Gobi regions, contain high fluoride. Fluoride treatment is limited, with only a few settlement wells equipped with reverse osmosis filters. The large, sparsely populated countryside relies on untreated water, and there is no systematic health risk assessment. Testing of all drinking wells, particularly in rural areas, is necessary.

In Nigeria, government agencies have made limited efforts to implement sustainable interventions or policies to reduce fluoride risks. Authorities should establish more effective programs to mitigate public health dangers from fluoride consumption. Researchers are encouraged to explore human health risk assessment, geostatistical, and machine learning models in future studies.

In Ghana, fluoride contamination is a significant public health concern, and policy interventions have been implemented to mitigate the problem (Ganyaglo et al., 2019; Maity et al., 2021). The Ghana Water Company Limited (GWCL) regulates water supply and distribution, operates a national water quality monitoring program to track fluoride concentrations, and implements treatment programs using defluoridation technologies such as activated alumina and bone char (Fuesta and Haffnerb, 2007).

In Kenya, water resources are managed at the county level. Despite natural contaminants being reported, no government-led water purification programs for defluoridation or desalination exist.

In Ethiopia, although awareness of fluoride contamination and health impacts is increasing, many people still rely on fluoride-rich water. Urban residents often receive treated water, while rural communities lack alternative sources and force to depend on known fluoride-rich water due to limited treatment infrastructure (Rango et al., 2012). USAID and NGOs have installed defluoridation systems, but sustainability is a challenge. When systems fail, communities often revert to previous water sources. Average monthly household income in Ethiopia is ~ $83, making investment in water infrastructure, fluoride removal, and storage facilities difficult (Entele & Lee, 2020). With such low income, most of the population cannot afford private reverse osmosis in their households.

Similar social disparities exist in Yemen, where no clear government policies ensure access to safe, fluoride-free water. Rural populations rely on alternative wells or springs, while wealthier families may have private wells. Most of the population cannot afford reverse osmosis systems, and privately-owned wells are not monitored. This exacerbates inequalities and the risk of fluorosis. Furthermore, commercial purified water is available but not regulated by authorities.

In Iran, urban and rural water supply is controlled by provincial water and sewage companies, adhering to national and international standards. Some villages rely on private wells due to poverty, and community-level fluoridation or defluoridation is generally not feasible. Wealthier residents may use private filtration systems, but these are often inefficient due to irregular maintenance. Government efforts exist, but information on de-fluoridation system capacity and mechanisms is limited.

In Türkiye, municipal wastewater treatment facilities use adsorption techniques (sand filters, activated carbon) rather than reverse osmosis to provide potable water. Implementation of low-cost defluoridation methods for large communities remain challenging.

In Mexico, many high-fluoride wells have been fitted with reverse osmosis filters, reducing fluorosis prevalence (Espino-Valdés et al., 2022). Dental fluorosis is now rare, but rural areas with untested wells remain at risk (Alarcón-Herrera et al., 2020; Espino-Valdés et al., 2022). Reverse osmosis generates 30–50% waste water with high salt content (Alarcón-Herrera & Gutiérrez, 2022; Olmos-Márquez et al., 2020). Alternative treatments, such as electrocoagulation, filtration, and adsorption using biomaterials, are still in pilot stages (Robledo-Peralta et al., 2021). Low-cost approaches like mixing groundwater with low-solute water (surface water, rainwater, solar-distilled water) are also in planning or pilot stages. Rural inhabitants are generally unaware of water quality risks. Drinking water management is at the municipal level, with national limits enforced at the federal level. Compliance enforcement is limited due to insufficient funds and personnel. The lowering of Mexico’s fluoride limit in drinking water from 1.5 to 1.0 mg L^−1^ (NOM-127-SSA1-2021) in 2021 makes more stringent treatment necessary (ITA, 2023; Valdivia Alvarado et al., 2021).

In Brazil, approximately 35 million people lack access to treated water, leading to multiple fluorosis cases and outbreaks of water-borne diseases (Hirata et al., 2019; Instituto Trata Brasil, 2023). Data on fluoride and fluorosis is limited in interior regions, though concerning cases have been reported in Centro-Oeste (Magalhães & Migliorini, 2021) and Norte (Amazon region) (Gonçalves et al., 2022; Rebelo et al., 2020; Santos, 2020). Public administration should aim for universal basic sanitation rather than localized solutions. Long-term dental and health safety will be best achieved through comprehensive water and sewage treatment.

## Research gaps, opportunities and recommendations

Though significant progress has been made in recent years in understanding the sources of fluoride and the extent of the most affected areas, important knowledge gaps remain. These gaps include testing fluoride concentrations in sparsely populated areas, determining the co-occurrence of other toxic ions, investigating the combined health effects of co-occurring solutes, and addressing social factors that influence the identification and implementation of practices to minimize health risks, particularly for the most affected low-income populations. Given the diversity of water problems among these countries, ranging from high fluoride to the presence of other local co-contaminants and groundwater over-exploitation, a single global solution to combat high fluoride and fluorosis risk is not feasible. Common approaches to mitigate fluoride include using alternative water sources, removing excess fluoride through ex-situ and in-situ methods, and modifying dietary intake (foods rich in calcium and vitamin D) to prevent fluorosis.

Although avoiding the source of fluoride is the most effective way to reduce fluorosis risk, it is often not feasible due to limited alternative water sources. Providing safe water access to every household remains a challenge, particularly in rural areas. Future investigations must focus on complex interplay of geogenic factors driving fluoride mobilization from surface dissolution of fluorapatite under circum-neutral pH conditions, silicate weathering, and calcium-deficient conditions to evaporative concentration and competitive desorption. Surface water availability is uncertain throughout the year, and groundwater remains a viable water supply option in many regions globally. Borewell construction should be carried out with careful consideration of hydrogeological conditions and variations in fluoride concentrations with depth to the water table (Brindha et al., 2011; Edmunds & Smedley, 2013). Considering the potential limitations of securing reliable sources, the infrastructure and costs involved in water treatment, and local conditions, exploring alternative approaches is warranted.

Despite significant advances in groundwater fluoride research, knowledge gaps persist, including: (i) lack of comprehensive data on temporal and spatial variations of fluoride levels in aquifers, (ii) limited understanding of the origins and causes of fluoride contamination, (iii) limited research on potential impacts of fluoride on aquatic ecosystems, (iv) limited research on the effects of elevated groundwater fluoride concentrations on plant and animal growth. v) different analytical methods used for determining fluoride concentration.

Future research should focus on conducting comprehensive studies to assess the spatial and temporal variability of fluoride in groundwater, especially in data-sparse regions.

The use of cost-effective and sustainable defluoridation techniques, along with community-based interventions, could help ensure safe drinking water in affected regions. Future research should prioritize identifying sources and causes of fluoride contamination to develop appropriate management strategies. Ndambiri and Rotich (2018) reported that in Kenya, communities are willing to pay nominal amounts to remove fluoride from drinking water, a similar trend also observed in other African countries such as Ethiopia and Tanzania. Implementing appropriate technologies, designing simple household-level defluoridation filters, and encouraging active participation of rural communities are few practical solutions in managing fluoride contamination. Community water supply schemes providing reverse osmosis water have been increasingly implemented in recent years. Additionally, selecting low-fluoride wells at the village level and distributing water from such wells is feasible due to uneven fluoride distribution. Ultimately, sustainable strategies require integrating hydrogeochemical mapping with decentralized treatment systems and regular water quality monitoring to protect vulnerable populations from chronic fluoride exposure.

Globally, numerous regions remain unexplored. While significant studies report on fluoride, many do not document its genesis and mobilization pathways. Understanding fluoride release under various physico-chemical conditions requires determination of sources and geochemical assessment of aquifers. This is essential for understanding global fluoride pathways in groundwater.

Data sharing remains a major challenge in fluoride research. This study recommends making water quality data openly accessible to improve understanding of fluoride contamination. Data deposit in a single repository could be a commendable step towards understanding fluoride mobility. International collaboration among researchers is essential and more powerful than isolated efforts. Establishing an international committee could address this issue not only for fluoride but also for other toxic pollutants. Associations such as the International Medical Geology Association (IMGA) provide a network and forum for collaboration among geologists, earth scientists, environmental scientists, toxicologists, epidemiologists, and medical specialists (https://medicalgeology.org/). Expanding and strengthening IMGA’s activities globally could help in tackling the fluoride contamination, particularly in developing countries.

Fluoride is indisputably a major global groundwater contaminant, and current defluoridation techniques are largely limited to local scales. Providing water to large communities faces challenges due to scalability, acceptability, and weak policies in developing nations. Open access to data would enable a better understanding of fluoride pathways on a large scale compared to individual studies.

## Conflict of interests

The authors declare no competing interests.

## Supplementary Information

Below is the link to the electronic supplementary material.

Supplementary file1 (DOCX 12010 kb)

## Data Availability

All data supporting the findings of this review are available within the cited published literature and publicly accessible sources.
