# Supplementary material for: Global groundwater contamination by geogenic fluoride
Source: Environ Geochem Health. 2026 Jun 17;48(9):410. doi: 10.1007/s10653-026-03187-8 (PMC13275634; doi:10.1007/s10653-026-03187-8)
Supplement: Supplementary file 1 — Supplementary file1 (DOCX 12010 kb) [file 10653_2026_3187_MOESM1_ESM.docx]

**Global Groundwater Contamination by Geogenic Fluoride**

**^a^Shakir Ali**

^a^Department of Civil Engineering Science, Faculty of Engineering & the Built Environment, Auckland Park, Kingsway Campus, University of Johannesburg, Johannesburg, South Africa

**Corresponding author:** shakiriitb@gmail.com; [shakira@uj.ac.za](mailto:shakira@uj.ac.za)

ORCID: <https://orcid.org/0000-0002-7743-9965>

**^b^Pratibha Mishra**

^b^School of Geosciences, The University of Edinburgh, Edinburgh, EH8 9XP, Scotland

**^c^K. Brindha**

^c^Department of Water Resources and Ecosystems, IHE Delft Institute for Water Education, Westvest 7, 2611 AX Delft, The Netherlands

**^d^Mélida Gutiérrez**

^d^School of Earth, Environment, and Sustainability, Missouri State University, USA

**^e^Rakesh Kumar**

^e^Department of Biosystems Engineering, Auburn University, Auburn, AL 36849, USA

**^f^Emmanuel Daanoba Sunkari**

^f^Department of Mining Engineering, Faculty of Integrated and Advanced Technology, Sir Padampat Singhania University, Udaipur-313601, Rajasthan, India

^f^Department of Chemical Sciences, Faculty of Science, University of Johannesburg, P.O. Box 524, Auckland Park 2006, Johannesburg, South Africa

**^g^Patrick Kirita Gevera**

^g^Department of Civil Engineering, University of South Africa, [Florida science campus], Cnr Christian de Wet Road and Pioneer Avenue, Johannesburg, South Africa

**^h^Enn Karro**

^h^Department of Geology, Institute of Ecology and Earth Sciences, University of Tartu

Ravila 14a, 50411 Tartu, Estonia

**^i^Johnbosco C. Egbueri**

^i^Department of Geology, Chukwuemeka Odumegwu Ojukwu University, Uli, Nigeria

Research Management Office (RMO), Chukwuemeka Odumegwu Ojukwu University, Anambra, Nigeria

**^j^Reza Dehbandi**

^j^Department of Chemical Engineering, University of Science and Technology of Mazandaran, Behshahr, Iran

^j^School of Geography, Earth and Environmental Sciences, University of Birmingham, Edgbaston, B15 2TT Birmingham, United Kingdom

**^k^Rohana Chandrajith**

^k^Department of Geology, Faculty of Science, University of Peradeniya, Sri Lanka

**^l^Peiyue Li**

^l^School of Water and Environment, Chang'an University, No. 126 Yanta Road, Xi'an 710054, Shaanxi, China

**^m^Abu Reza Md. Towfiqul Islam**

^m^Department of Disaster Management,

Begum Rokeya University, Rangpur, 5404, Bangladesh

**^n^Seong-Taek Yun**

^n^Department of Earth and Environmental Sciences, Korea University, Seoul 02841, South Korea

**^o^Hullysses Sabino**

^o^Geography Department, Geosciences Institute, Universidade Federal Fluminense, Rio de Janeiro, Brazil

**^p^Daniel Emilio Martinez**

^p^Instituto de Geología de Costas y del Cuaternario (U.N. Mar del Plata-CIC BA) - Instituto de Investigaciones Marinas y Costeras (CONICET-U.N. Mar del Plata) Mar del Plata, Argentina.

**^q^Alper Baba**

^q^Department of the international water resources, Izmir Institute of Technology, Izmir, Türkiye

**^r^Taimoor Shah Durrani**

^r^Department of Environmental Sciences, Balochistan University of Information Technology Engineering and Management Sciences (BUITEMS), Quetta, Pakistan

**^s^Vahab Amiri**

^s^Department of Geology, Yazd University, Iran

**^t^Adnan Aqeel**

^t^Department of Earth & Environmental Sciences, Sana'a University, Yemen

**^u^Julian Ijumulana**

^u^DAFWAT Research Group, Department of Water Resources Engineering, College of Engineering and Technology, University of Dar es Salaam, Dar es Salaam, Tanzania.

^u^Geospatial Data Sciences and Technology (Geomatics) section, Department of Transportation and Geotechnical Engineering, College of Engineering and Technology, University of Dar es Salaam, Dar es Salaam, Tanzania

**^v^Joshua Nosa Edokpayi**

^v^Water and Environmental Management Research Group, Faculty of Science, Engineering and Agriculture, University of Venda, Thohoyandou 0950, Limpopo province, South Africa

**^w^David Schafer**

^w^Department of Water and Environmental Regulation, Perth, Western Australia

**^x^Lidia Razowska-Jaworek**

^x^Polish Geological Institute - National Research Institute, Poland

**^y^Maria Teresa Alarcón-Herrera**

^y^Centro de Investigaciones de Materiales Avanzados, Durango, Dgo.34147, México

**^z^Odsuren Batdelger**

^z^Institute of Geography and Geoecology, Mongolian Academy of Sciences, Mongolia

**^aa^Ritusmita Goswami**

^aa^Centre for Ecology Environment and Sustainable Development, Tata Institute of Social Sciences, Guwahati, Assam, India

**^ab^Abida Farooqi**

^ab^Environmental Hydro Geochemistry Lab, Department of Environmental Sciences, Quaid-i-Azam University, Islamabad, Pakistan

**^ac^Emiliano Fabio Alcaraz**

^ac^Instituto de Geología de Costas y del Cuaternario (U.N. Mar del Plata-CIC BA) - Instituto de Investigaciones Marinas y Costeras (CONICET-U.N. Mar del Plata) Mar del Plata, Argentina.

**^ad^Yaşar Kemal Recepoğlu**

^ad^Department of Chemical Engineering, Izmir Institute of Technology, Izmir, Türkiye

^ad^Department of Chemical Engineering, Ege University, Izmir, Türkiye

**^ae^Soraya Paz-Montelongo**

^ae^Área de Toxicología, Universidad de La Laguna, La Laguna, 38071 Tenerife, Islas Canarias, Spain

**^af^Prosun Bhattacharya**

^af^KTH-International Groundwater Arsenic Research Group, Department of Sustainable Development, Environmental Science and Engineering, KTH Royal Institute of Technology, Teknikringen 10B, SE-114 28 Stockholm, Sweden

***SUPPLEMENTARY FIGURES***


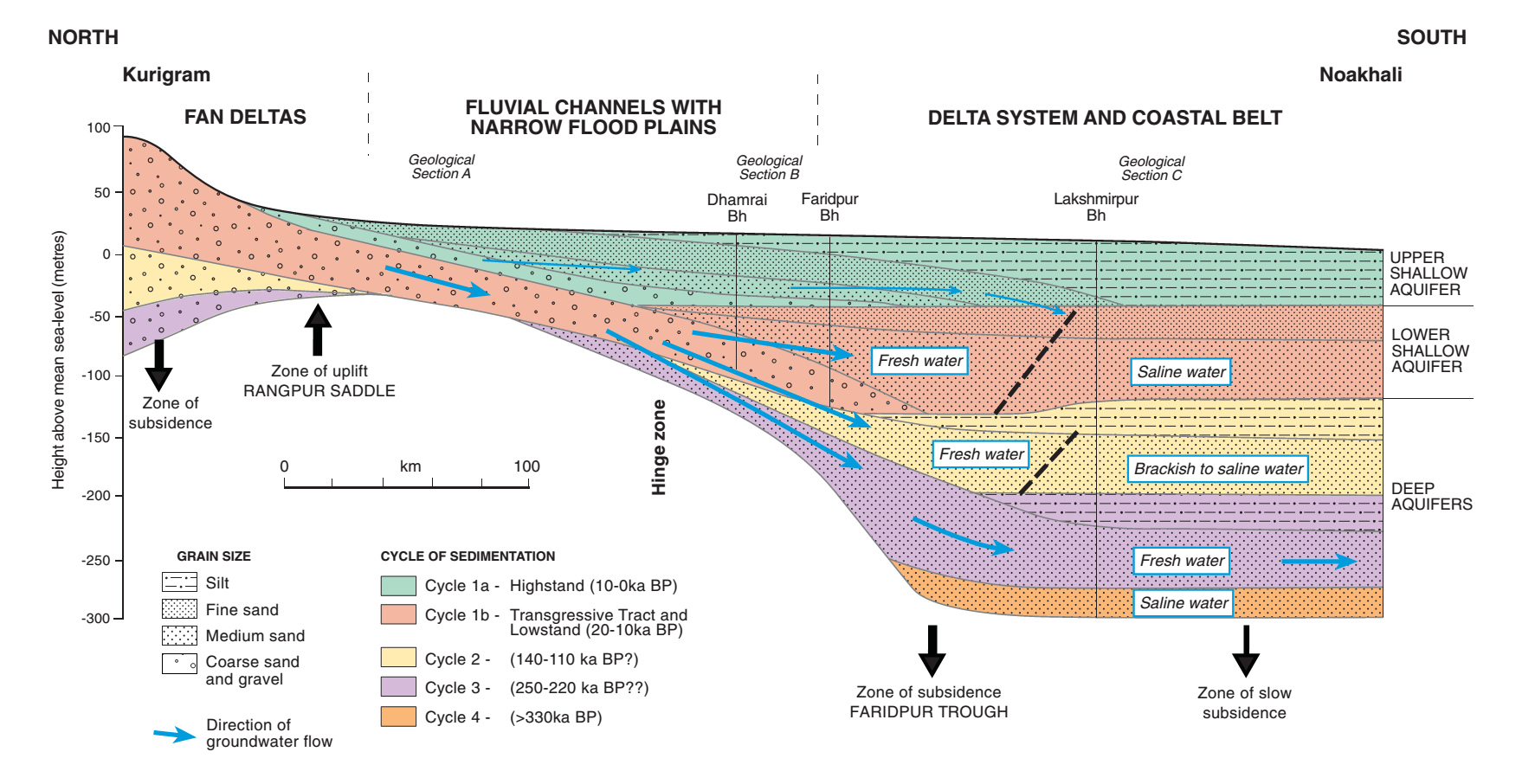


**Supp. Figure S1**: Hydrogeological cross-section from the north to the south part of Bangladesh (after BGS-DPHE 2001).


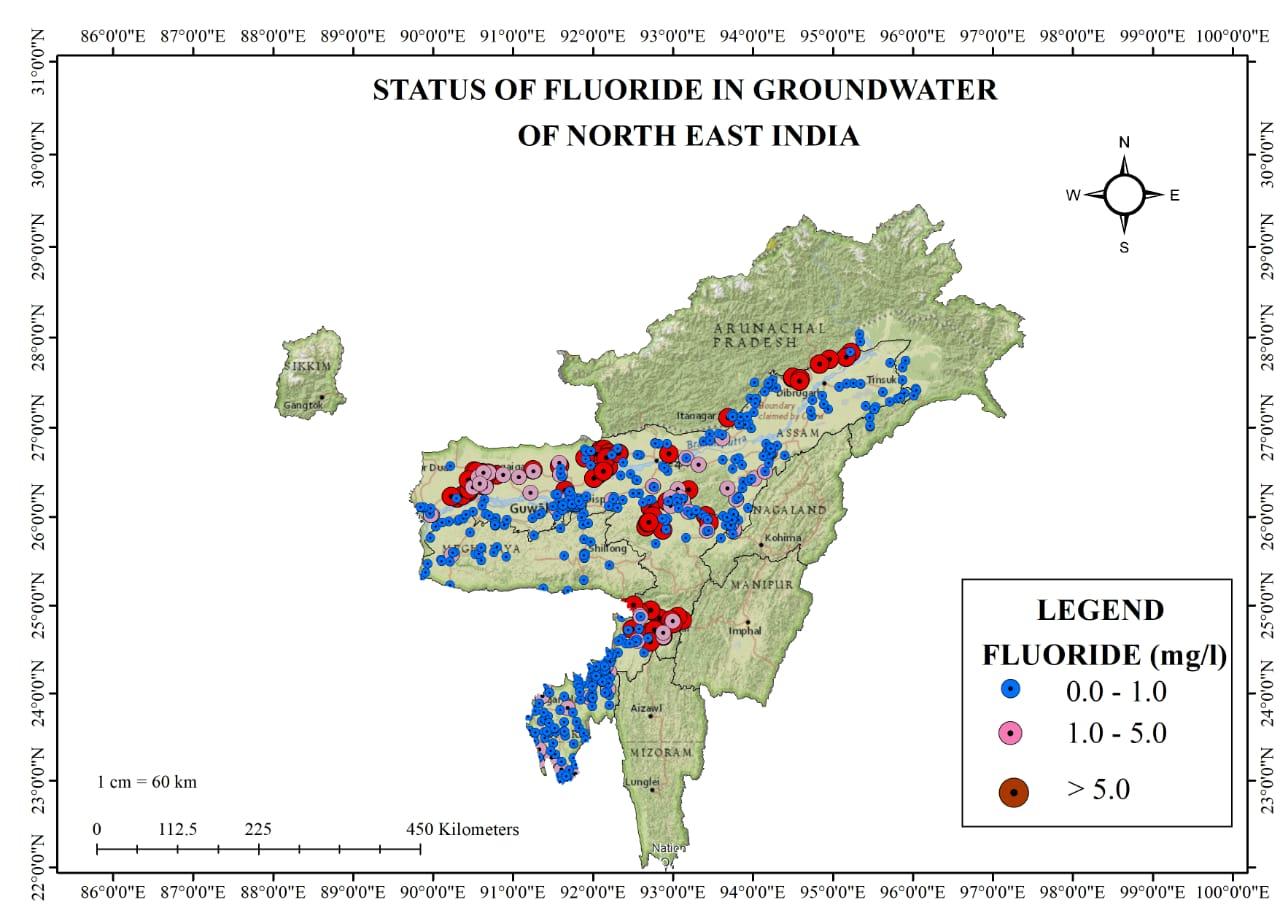


**Supp. Figure S2**: Fluoride in groundwater of Northeast India (N=976).

**Figure S SEQ Figure_S \* ARABIC 3**: Lithological units and groundwater fluoride distribution in Pakistan.

**
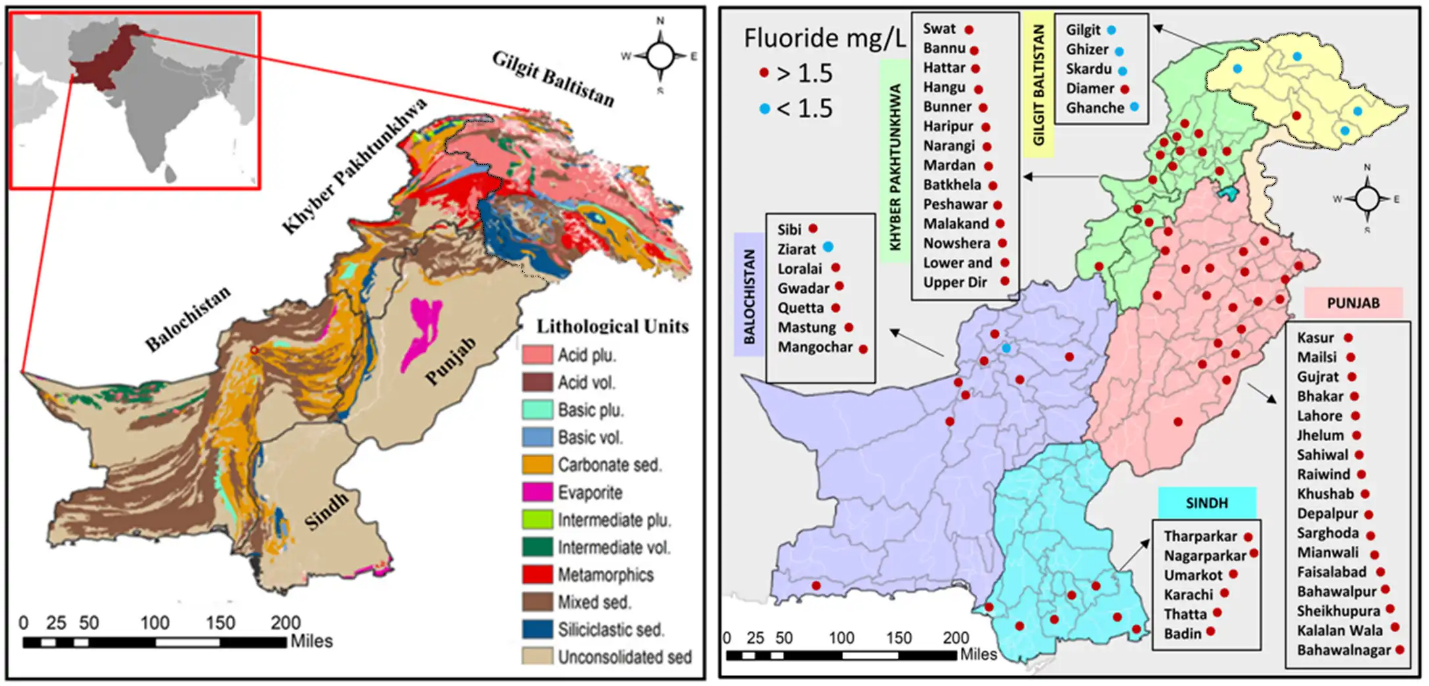
**

**Supp. Figure S3**: Lithological units and groundwater fluoride distribution in Pakistan. Disputed boundaries are indicated by dotted lines. The boundaries, names, and designations shown on this map are for academic purposes only and do not imply official endorsement or acceptance by the author or publisher. The author remains neutral regarding jurisdictional claims and boundary disputes.


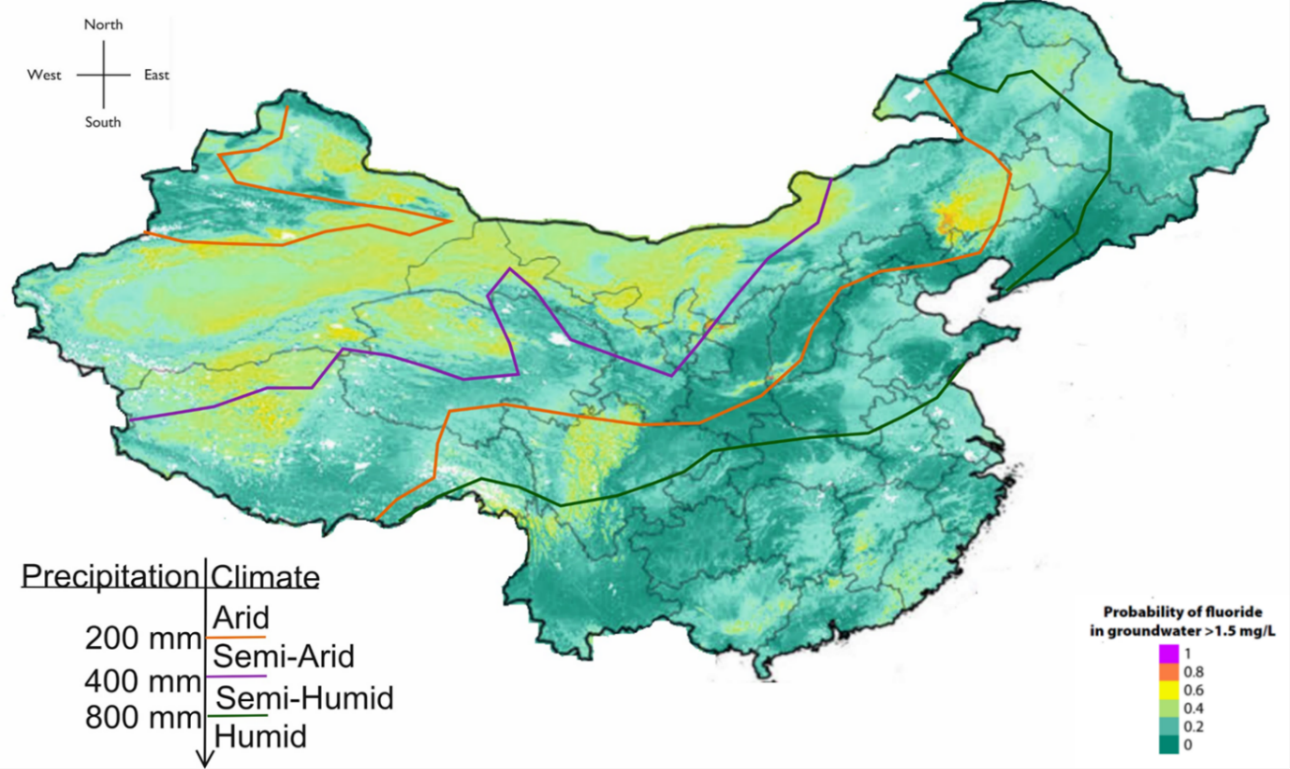


**Supp. Figure S4**: Probability of fluoride in groundwater of China [adapted from Podgorski & Berg (2022) open access; Precipitation and climate were adopted from He et al. (2020); not to scale.


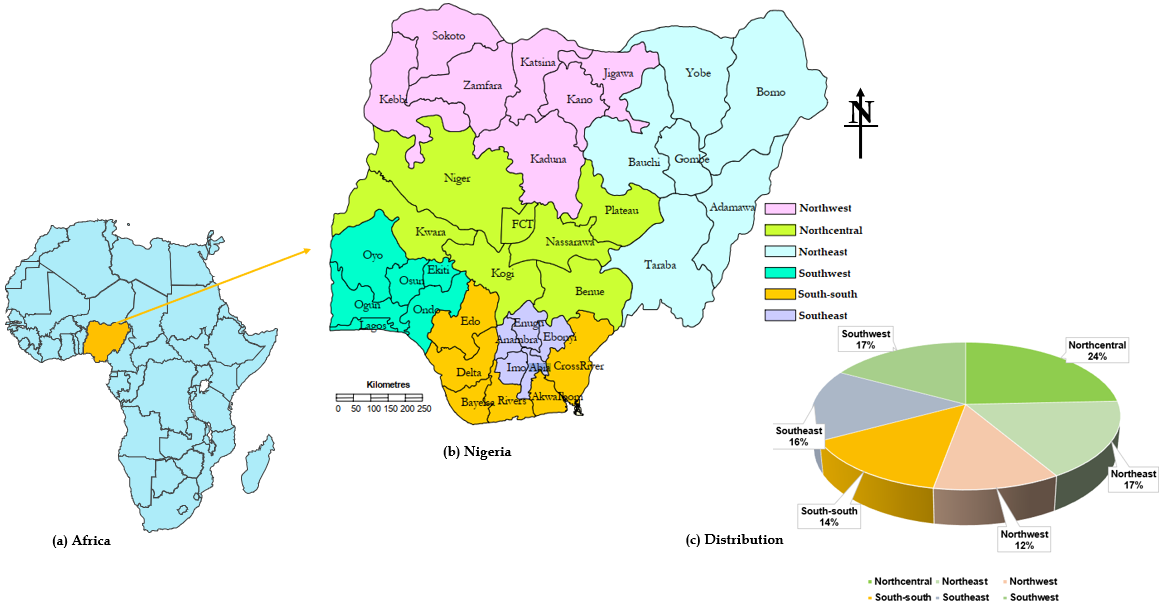


**Supp. Figure S5:** Location of Nigeria in Africa and the distribution of Nigerian fluoride studies depicted in a pie chart.


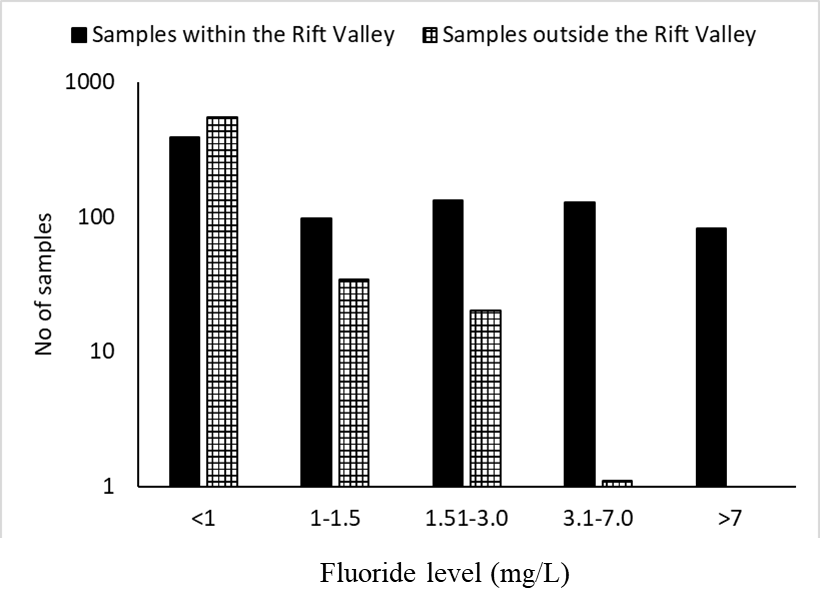


**Supp. Figure S6:** Fluoride distribution across Ethiopia from 1438 sampling sites both within and outside the rift valley system.


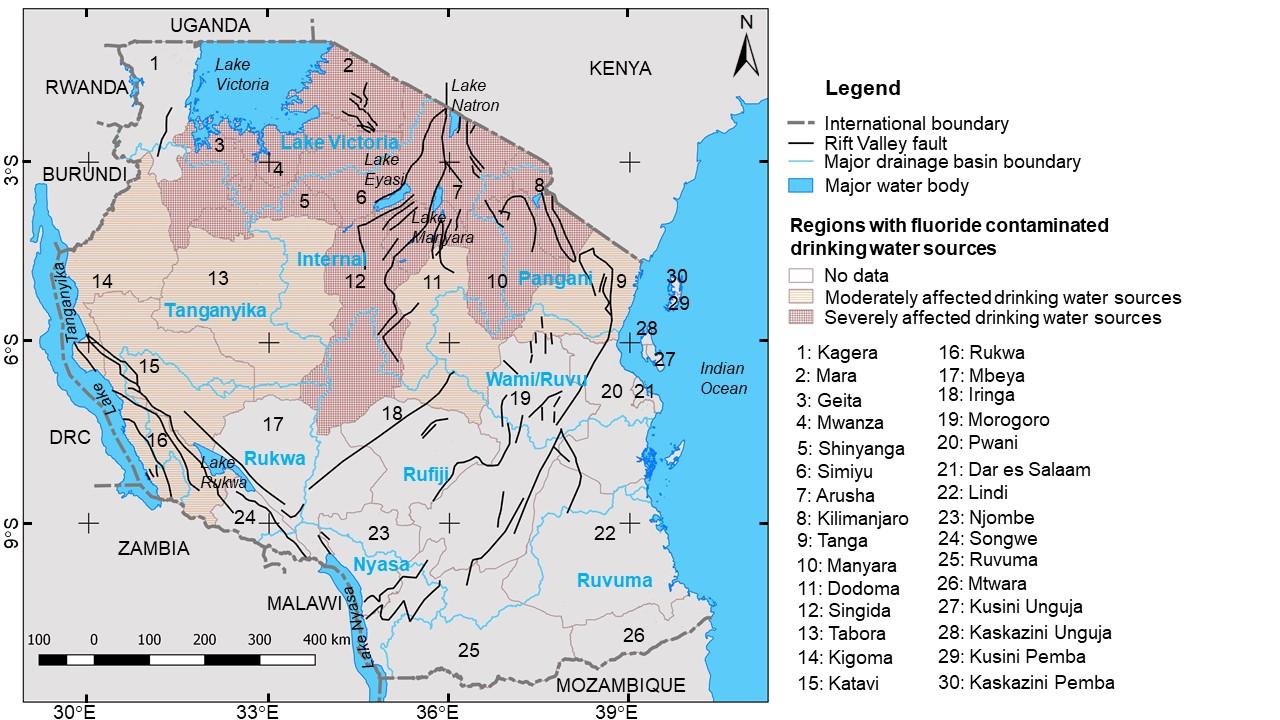


**Supp. Figure S7**: Geographical distribution of regions moderately and severely affected by excessive fluoride in the water sources of Tanzania (Ijumulana et al., 2022; open access).


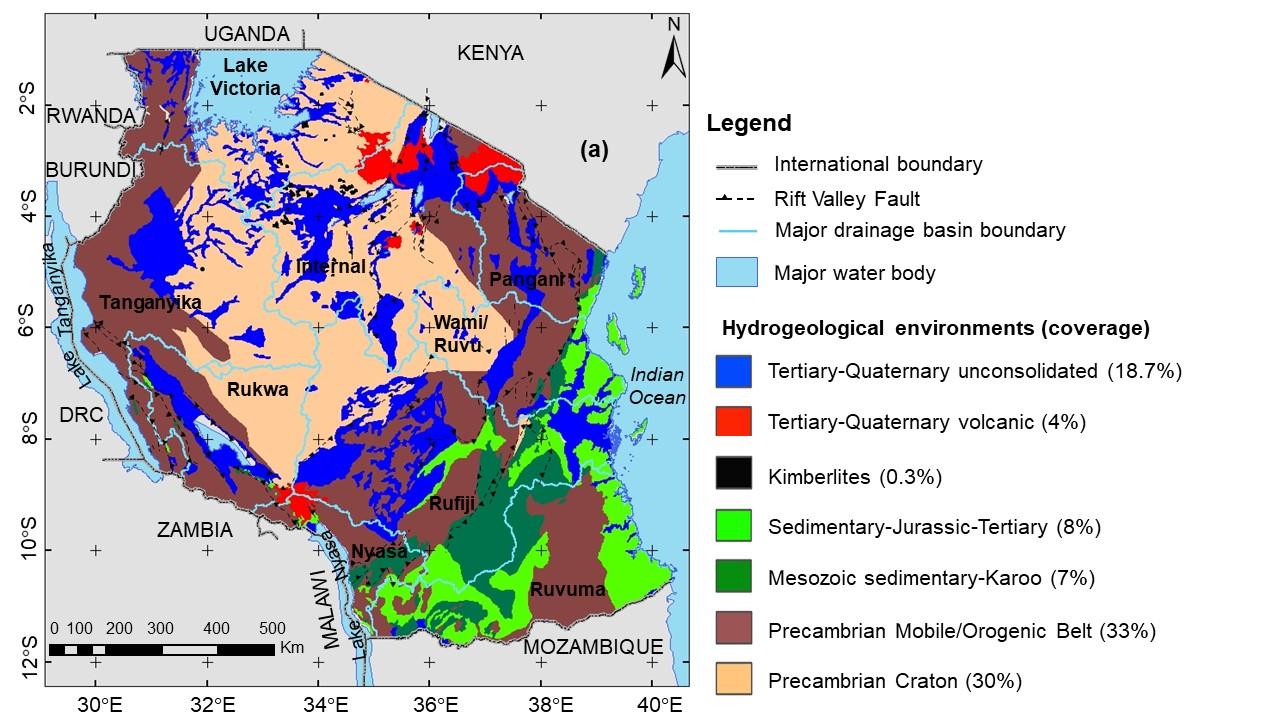


**Supp. Figure S8**: The generalized hydrogeological setting of Tanzania (Ijumulana et al., 2022; open access). (The hydrogeological data from Africa Groundwater Atlas (2019), available at https://africagroundwateratlas.org/). Kindly note that the rest (6%) is water bodies.


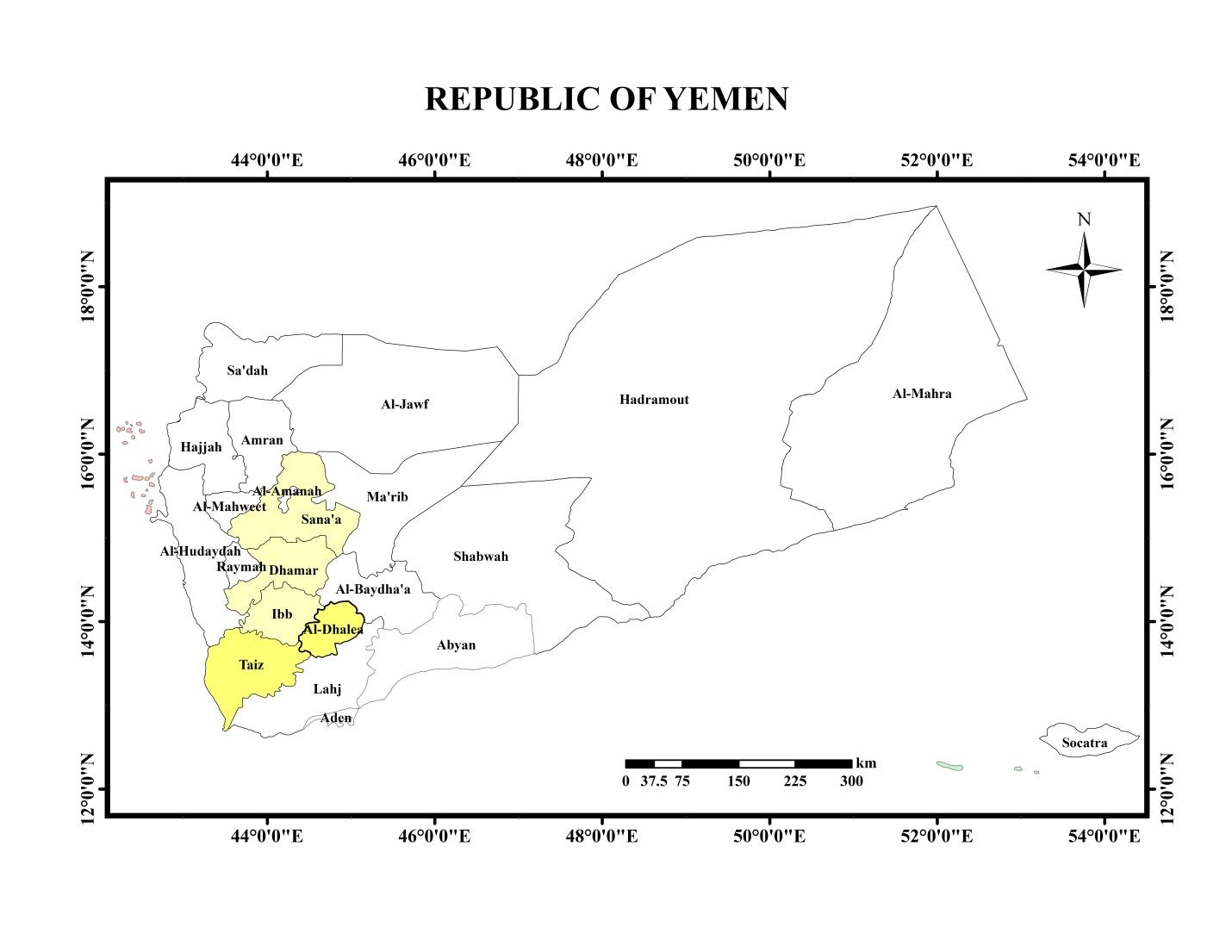


**Supp. Figure S9**: Spatial distributions of high concentrations of fluoride in drinking water in Yemen Governorates (Yellow colours).


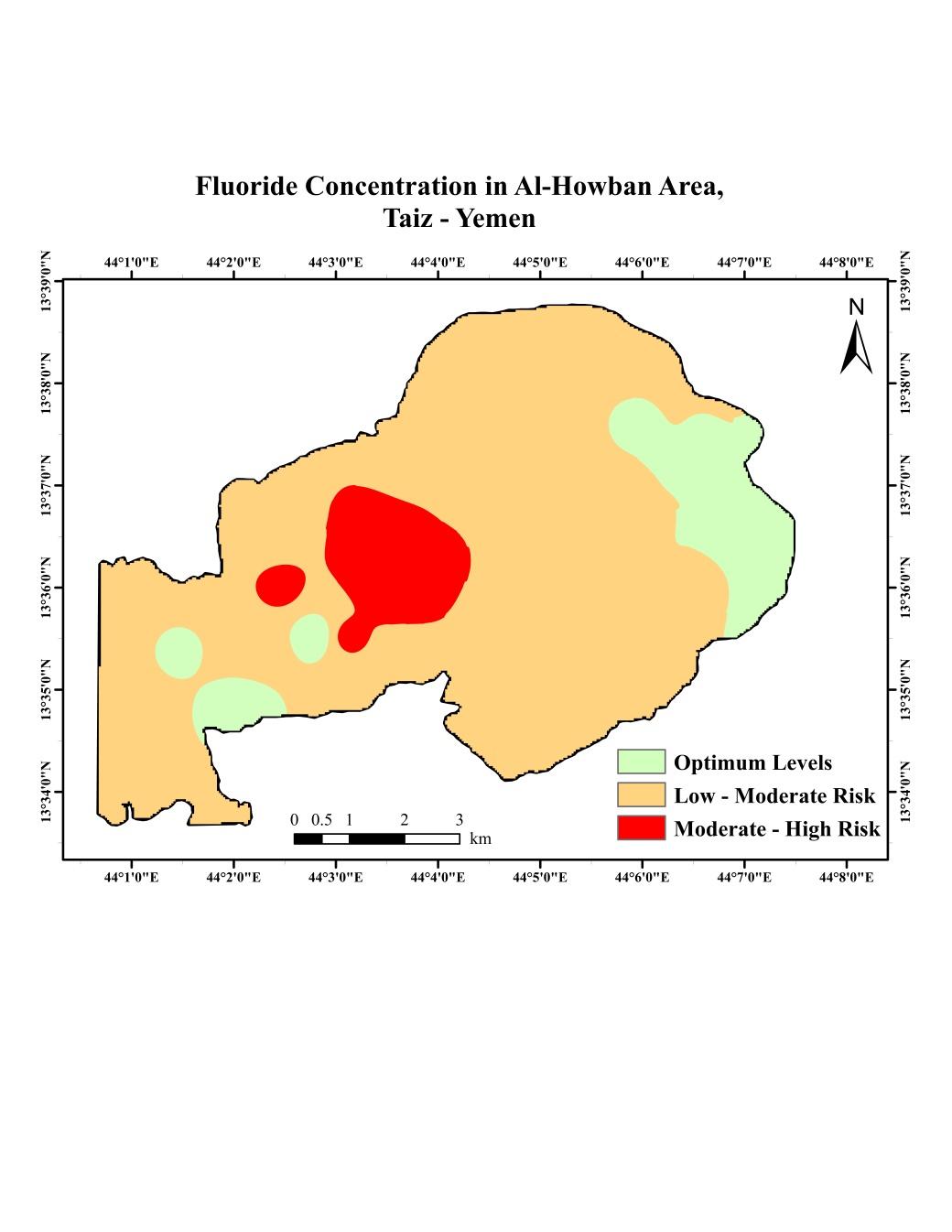


**Supp. Figure S10**: Map showing the fluorosis risk-levels in Taiz City (At-Taizyah District) in Taiz Governorate, Yemen. This map was produced based on the studies of both Aqeel et al., 2017 and; NWRA/Taiz (Natural Water Resources Authority), 2008).

**
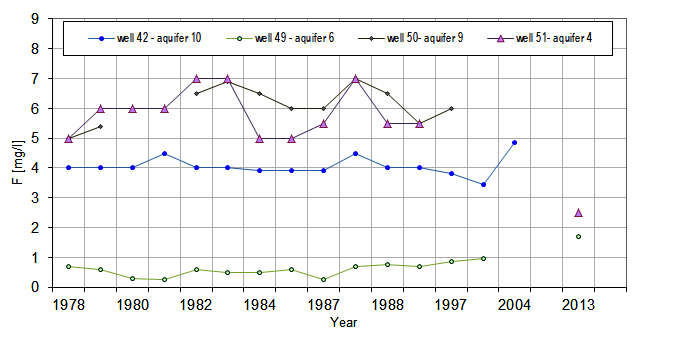
**

**Supp. Figure S11**: Fluoride concentrations in selected Neogene wells of Nysa Water Works, Poland (after Koślacz, 1989).


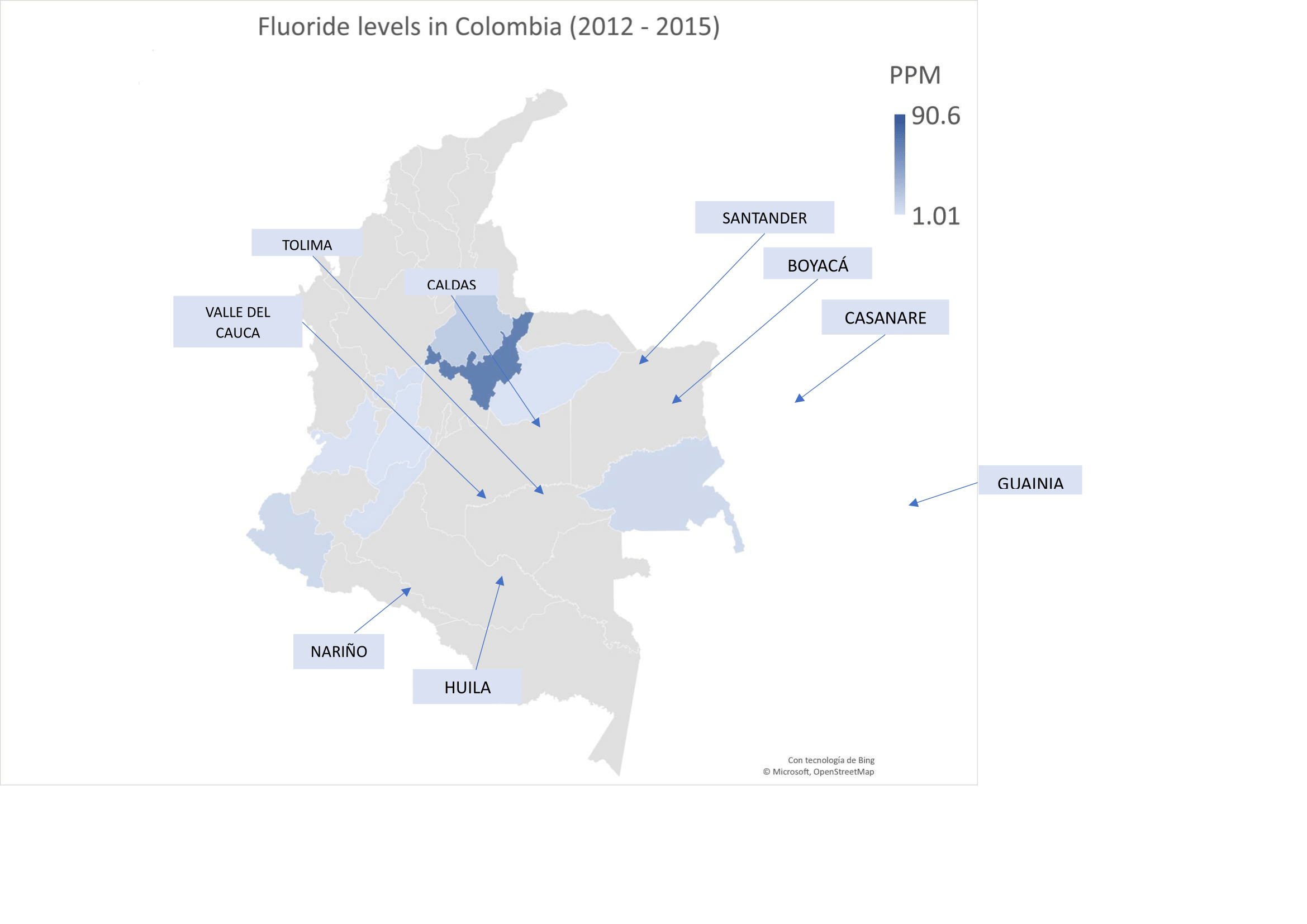


**Supp. Figure S12**: Fluoride levels in different regions of Colombia Source: MINSALUD, 2016.


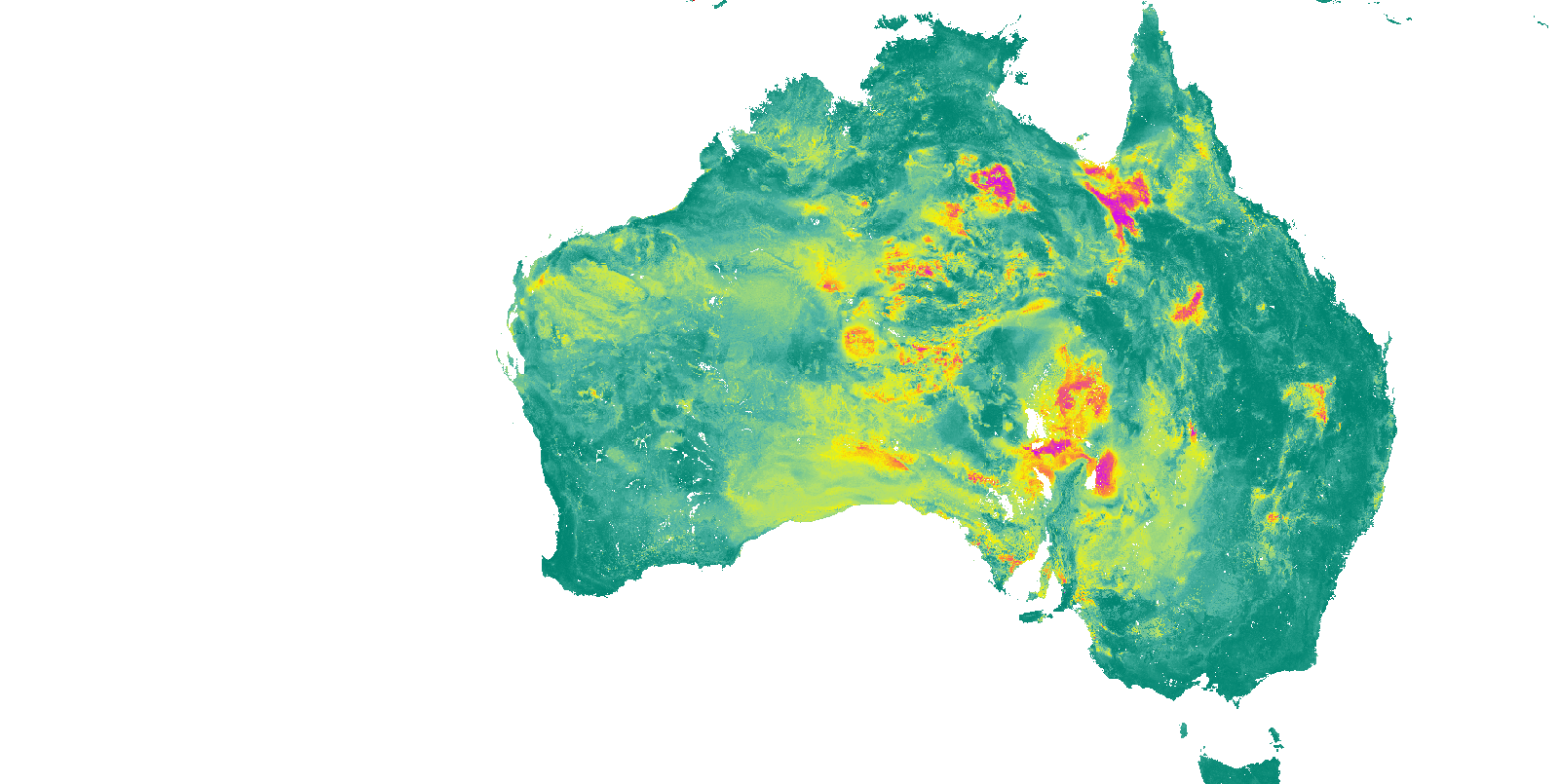

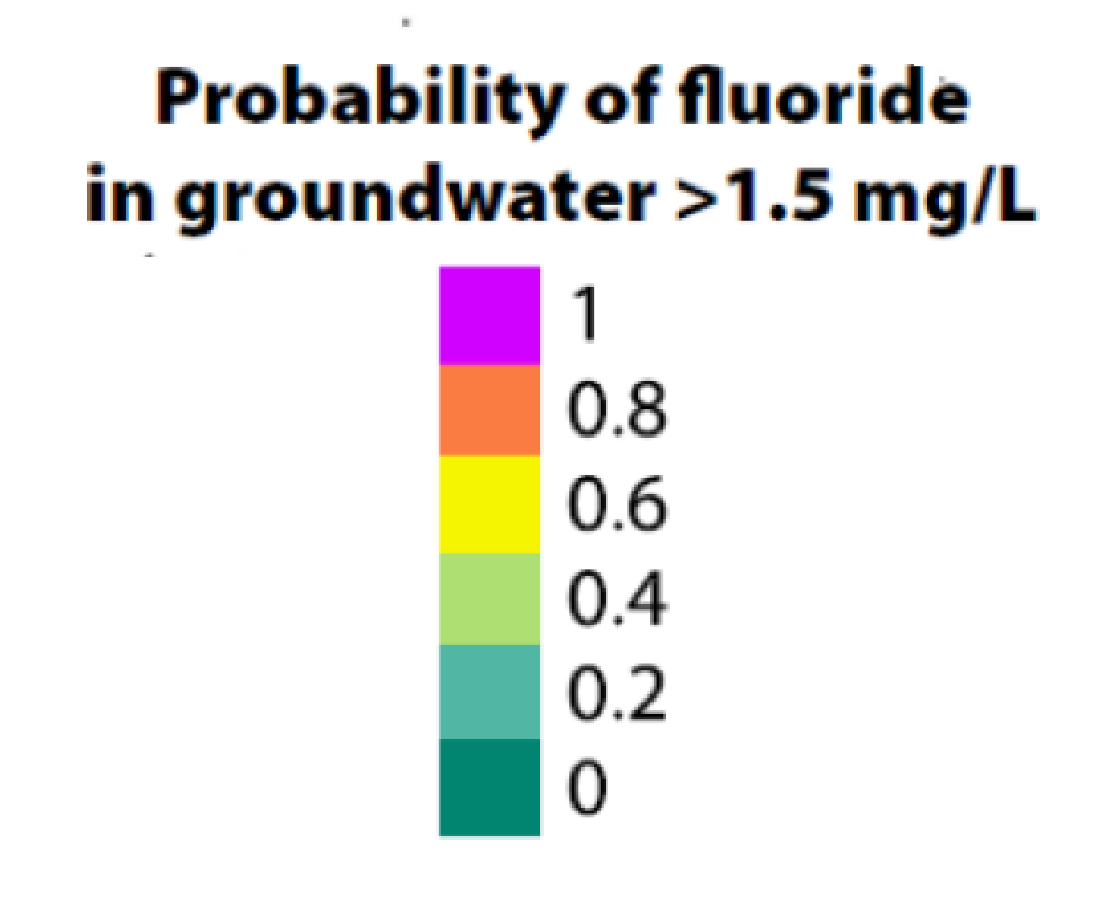


**Supp. Figure S13:** Probability of Fluoride in groundwater (adapted from Podgorski & Berg, 2022; open access).


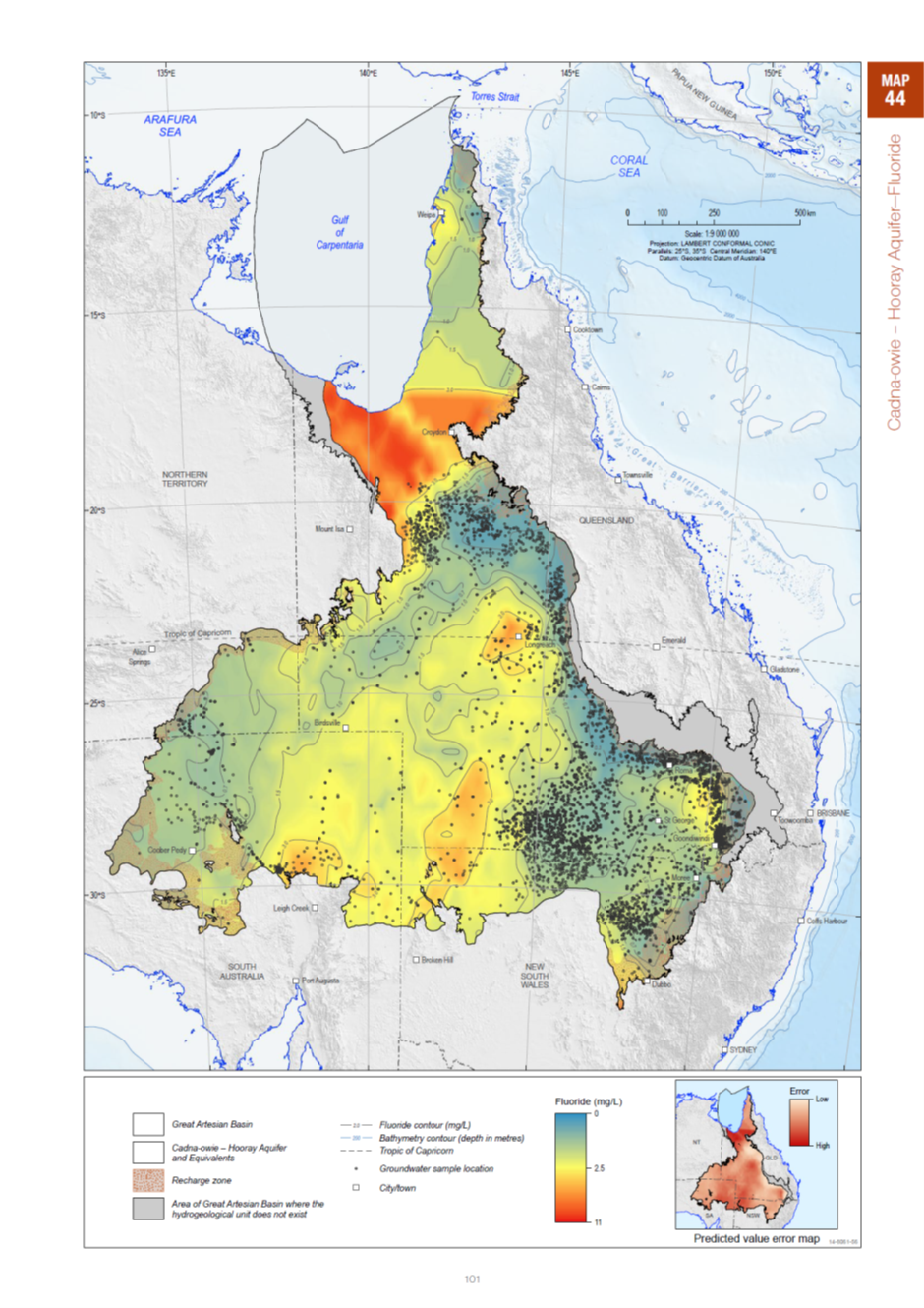


**Supp. Figure S14**: Distribution of fluoride in the Cadna-owie / Hooray Aquifer of the Great Artesian Basin of Australia (after Map 44 in Ransley et al., 2015), Commonwealth of Australia, Geoscience Australia. ([www.ga.gov.au](http://www.ga.gov.au)). Permission granted for the reuse of the figure through email.

**SUPP TABLES**

| **Supp. Table S1**: Fluoride concentration in fertilizers and their by-products. | | |
| --- | --- | --- |
| **Product** | **Fluoride (mg/Kg)** | **Reference** |
| Urea | 3.8 | Rao (1997) |
| Superphosphate | 2750 | Rao (1997) |
| NPK | 1675 | Rao (1997) |
| Potash | 10 | Rao (1997) |
| Zinc sulphate | 2.58 | Brindha & Elango (2013) |
| Potash | 13.77 | Brindha & Elango (2013) |
| Ammonium sulphate | 0.27 | Brindha & Elango (2013) |
| Urea | 0.42 | Brindha & Elango (2013) |
| NPK complex | 0.02 | Brindha & Elango (2013) |
| Diammonium phosphate (*DAP*) | 60-255 | Farooqi et al. (2007b) |
| Superphosphate, Triple superphosphate and NPK complex | Up to 10,000 | Mirlean & Roisenberg (2007) |
| Simple superphosphate | 420-510 | Da Conceição & Bonotto (2006) |
| Triple superphosphate | 492-546 | Da Conceição & Bonotto (2006) |
| Monoammonium phosphate | 330-372 | Da Conceição & Bonotto (2006) |
| Phosphogypsum, by-product of fertilizer production | 180-210 | Da Conceição & Bonotto (2006) |
| Phosphogypsum, by-product of fertilizer production | 2320 - 3400 | Dartan et al. (2017) |

**Supp. Table S2:** Spatial distribution range and probable release mechanism of Fluoride in North East India.

| **State** | **District** | **Fluoride concentration (mg/L)** | | **Probable release mechanism** | **References** |
| --- | --- | --- | --- | --- | --- |
|  |  | **Min** | **Max** |  |  |
| **Assam** | Karbi-Anglong | 0.15 | 20.6 | Dissolution from fluoride-containing minerals in the granitic rocks and regional geological settings; carbonate dissolution | (Gogoi et al., 2021; Hanse et al., 2019; Kakoty et al., 2008; Sahadevan & Chandrasekharam, 2008) |
|  | Nalbari | 0.02 | 1.56 | Dissolution from fluoride-bearing minerals such as Fluorspar, Cryolite and Fluroapatite in rock | (Sharma et al., 2012) |
|  | Nagaon | BDL | 9 | Silicate weathering and ion exchange | (Gogoi et al., 2021) |
|  | Kamrup (M) | BDL | 8.92 | Geogenic processes- dissolution of Fluoroapatite, Carbonate weathering and reverse ion exchange mechanism; input from fertilizers | (Chakrabarty & Sarma, 2011; Dutta et al., 2020; Gogoi et al., 2021) |
|  | Golaghat | 0.19 | 1.67 | Residual components of the igneous rocks such as feldspar, hornblende, and pyroxene | Sharma et al. (2011) |
| **Manipur** | Bishnupur | 0.02 | 1.6 | Silicate and carbonate rock-weathering or dissolutions processes | (Alam et al., 2020a) |
|  | Churachandpur | 0.1 | 1.3 | Rock-weathering geochemical process | (Alam et al., 2022) |
|  | Chandel | BDL | 1.65 | Rock weathering and silicate weathering process | (Alam et al., 2020b) |
|  | Imphal | 0.7 | 0.84 | Sources are the minerals in rocks, namely fluorite, fluorapatite, cryolite, mica & hornblende. | (Devi & Kamble, 2006) |
|  | Thoubal | BDL | 1.52 | Rock-weathering geochemical process | (Alam et al., 2019) |
| **Mizoram** | Aizawl | < | 1.5 | Weathering of fluoride-bearing minerals from rocks: arenaceous and argillaceous type | (Lalbiakmawia & Vanthangliana, 2015) |
| **Nagaland** | Dimapur | 0.02 | 6.3 | N/A | (Pamei et al., 2020) |
| **Tripura** | North Tripura | <0.005 | 4.8 | N/A | (Bhattacharya et al., 2020) |

N/A: Not available

| Supp. Table S3: Groundwater fluoride distribution in provinces of Pakistan | | | |
| --- | --- | --- | --- |
| Province | **Geology Units** | **Districts affected** | **Fluoride (mg/L)** |
| Balochistan | unconsolidated, mixed carbonate sediments, igneous, metamorphic | 7 | 0 to 24 |
| Gilgit Baltistan | sedimentary, igneous, metamorphic | 1 | 0 to 1.8 |
| Khyber Pakhtunkhwa | unconsolidated, mixed carbonate sediments, igneous, metamorphic | 12 | 0 to 6.7 |
| Punjab | unconsolidated, mixed carbonate sediments, evaporites, igneous | 20 | 0 to 21 |
| Sindh | unconsolidated mixed sediments, evaporates, igneous | 6 | 0.06 to 44.4 |

**Supp. Table S4:** Previous studies that have reported fluoride contamination in Nigeria.

| Author(s) | Locality | Region | Level of Fluoride in water (mg L^-1^) | Water type |
| --- | --- | --- | --- | --- |
| (Emenike et al., 2018) | Abeokuta | Southwest | 0.48 to 1.84 | Groundwater |
| (Ogbu et al., 2012) | Enugu | Southeast | Mean = 0.63 | Groundwater |
| (Olasehinde et al., 2016) | Ogbomosho | Northcentral | 1.35 to 2.75 | Groundwater |
| (Aminu & Amadi, 2014) | Zango, Katsina | Northwest | 0.10 to 3.16 | Groundwater |
| (Okunlola et al., 2016) | Hong area, Adamawa | Northeast | 0.06 to 2.58 | Groundwater |
| (Lar et al., 2007; Uriah et al., 2014) | Fobur, Jos East | Northcentral | 0.2 to 8 | Surface and groundwater |
| (Dibal & Lar, 2005) | Kaltungo, Gombe | Northeast | 1 to 4 | Groundwater |
| (Dibal & Lar, 2005) | Kerang volcanic province | Northcentral | 0.14 to 0.41 | Spring water |
| (Dibal & Lar, 2005) | Kerang volcanic province | Northcentral | 0.12 to 0.59 | Groundwater |
| (Egbinola & Amanambu, 2014) | Ibadan | Southwest | 0.01 to 6.38 | Groundwater |
| (Akpabio et al., 1984) | Kaduna State | Northwest | 0.19 to 0.42 | Groundwater |
| (Akpabio et al., 1984) | Kwara State | Northcentral | 0.06 to 0.42 | Groundwater |
| (Akpabio et al., 1984) | Niger State | Northcentral | 0.02 to 0.20 | Groundwater |
| (Akpabio et al., 1984) | Abuja (FCT) | Northcentral | 0.12 to 0.24 | Groundwater |
| (Akpabio et al., 1984) | Sokoto | Northwest | 0.29 to 0.60 | Groundwater |
| (Akpabio et al., 1984) | Oyo State | Southwest | 0.12 to 0.20 | Groundwater |
| (Akpabio et al., 1984) | Lagos State | Southwest | 0.25 | Groundwater |
| (Akpabio et al., 1984) | Ogun State | Southwest | 0.12 to 0.30 | Groundwater |
| (Akpabio et al., 1984) | Bendel State (now Delta) | South-south | 0.03 to 0.05 | Groundwater |
| (Akpabio et al., 1984) | Anambra State | Southeast | 0.05 to 0.08 | Groundwater |
| (Akpabio et al., 1984) | Borno State | Northeast | 0.05 to 1.52 | Groundwater |
| (Ibe et al., 1999) | Ohafia-Arochukwu area | Southeast | 0.00 to 0.03 | Spring and stream waters |
| (Ndukwe et al., 2019) | Ikwuano Region | Southeast | 0.40 to 2.0 | Groundwater |
| (Goyit et al., 2018) | Langtang North | Northcentral | 1.1 to 6.0 | Surface and groundwater |
| (Thompson, 1958) | Gombe area | Northeast | 0.0 to 2.1 | Groundwater |
| (Oteze & Ayegbusi, 2002) | Maiduguri | Northeast | 5.00 | Groundwater |
| (Hyeladi et al., 2014) | Jos Plateau | Northcentral | 0.01 to 1.56 | Groundwater |
| (Dibal et al., 2012) | Langtang area | Northcentral | 0.12 to 10.30 | Hand dug wells, streams, boreholes, and spring |
| (Giwa et al., 2021) | Gombe State | Northeast | 0.35 to 3.46 | Groundwater |
| (Dirisu et al., 2016) | Omoku, Rivers State | South-south | 0.94±0.07 | Private borehole water |
| (Dirisu et al., 2016) | Omoku, Rivers State | South-south | 0.48±0.03 | Dug well water |
| (Lar & Gusikit, 2015) | Panyam volcanic province | Northcentral | 0.12 to 0.59 | Boreholes and springs |
| Ibiyemi 2020 | Oyo State | Southwest | 0.03 to 3.00 | Groundwater |
| (Akpata et al., 2009) | Northcentral | Northcentral | 0.96±0.81 | Shallow wells |
| (Akpata et al., 2009) | Northeast | Northeast | 0.33±0.22 | Shallow wells |
| (Akpata et al., 2009) | Northwest | Northwest | 0.26±0.22 | Shallow wells |
| (Akpata et al., 2009) | South-south | South-south | 0.28±0.76 | Shallow wells |
| (Akpata et al., 2009) | Southeast | Southeast | 0.21±0.08 | Shallow wells |
| (Akpata et al., 2009) | Southwest | Southwest | 0.26±0.19 | Shallow wells |
| (Akpata et al., 2009) | Northcentral | Northcentral | 0.67±1.28 | Deep wells |
| (Akpata et al., 2009) | Northeast | Northeast | 0.36±0.30 | Deep wells |
| (Akpata et al., 2009) | Northwest | Northwest | 0.33±0.28 | Deep wells |
| (Akpata et al., 2009) | South-south | South-south | 0.24±0.38 | Deep wells |
| (Akpata et al., 2009) | Southeast | Southeast | 0.18±0.05 | Deep wells |
| (Akpata et al., 2009) | Southwest | Southwest | 0.33±0.35 | Deep wells |
| (Akpata et al., 2009) | Northcentral | Northcentral | 0.68±0.75 | Boreholes |
| (Akpata et al., 2009) | Northeast | Northeast | 0.62±0.67 | Boreholes |
| (Akpata et al., 2009) | Northwest | Northwest | 0.34±0.24 | Boreholes |
| (Akpata et al., 2009) | South-south | South-south | 0.53±1.10 | Boreholes |
| (Akpata et al., 2009) | Southeast | Southeast | 0.38±0.42 | Boreholes |
| (Akpata et al., 2009) | Southwest | Southwest | 0.63±1.05 | Boreholes |

**Supp. Table S5**: Summary of highly fluoridated zones in Ghana (after Sunkari et al., 2022)

| **Location** | **Groundwater fluoride concentration (mg L^-1^)** | **Reference** |
| --- | --- | --- |
| Upper East Region | 0.10-5.00 | Alfredo et al., 2014; Apambire et al., 1997; Atipoka, 2009; Craig et al., 2015, 2018; Firempong et al., 2013; Ganyaglo et al., 2019; Sunkari et al., 2018; Sunkari & Abu, 2019; Zango et al., 2021 |
| North East Region | 0.01-13.29 | Anornu et al., 2017; Sunkari et al., 2020; Zango et al., 2019 |
| Northern Region | 0.1-11.6 | Salifu et al., 2016; Sunkari et al., 2023; Tay, 2017 |
| White Volta River Basin | 0.04-3.79 | Anornu et al., 2017 |

**Supp. Table S6:** Fluoride levels in the rift and highland systems of 126 communities from 270 water sources (Kloos & Tekle Haimanot, 1999)

| Fluoride  (mg L^-1^) | No. of sources | Hot Spring | Cold spring | Boreholes | Shallow wells | Rivers | Lakes |
| --- | --- | --- | --- | --- | --- | --- | --- |
| Rift Valley system | | | | | | | |
| BDL | 4 | 0 | 0 | 3 | 0 | 0 | 0 |
| <1 | 43 | 0 | 6 | 30 | 3 | 3 | 1 |
| 1.0-4.9 | 52 | 0 | 1 | 42 | 2 | 6 | 1 |
| 5.0-9.9 | 24 | 1 | 0 | 20 | 2 | 0 | 1 |
| >9.9 | 29 | 2 | 0 | 18 | 4 | 0 | 6 |
| Subtotal | 152 | 3 | 7 | 113 | 11 | 9 | 9 |
| Highland’s region | | | | | | | |
| BDL | 13 | 0 | 1 | 11 | 0 | 1 | 0 |
| <1 | 84 | 0 | 23 | 40 | 2 | 16 | 3 |
| 1.0-4.9 | 18 | 0 | 3 | 9 | 1 | 4 | 1 |
| 5.0-9.9 | 3 | 0 | 0 | 3 | 0 | 0 | 0 |
| >9.9 | 0 | 0 | 0 | 0 | 0 | 0 | 0 |
| subtotal | 118 | 0 | 27 | 63 | 3 | 21 | 4 |

BDL: below detection limit

**Supp. Table S7- Input parameters of the non-carcinogenic risk assessment**

|  | Value | Units | Parameters |
| --- | --- | --- | --- |
| - | - | mg/L | Concentration (C) |
| Bazeli et al. (2022) | **Cold regions:** Children: 0.51, Teen: 1.12, Adults: 1.23 | l/day | Ingestion rate (IRw) |
| Mohammadpour et al. (2022) | **Hot regions:**  Children: 1.25, Teen: 1.58, Adults: 1.95 |  |  |
| EPA (2024) | 365 | Day/year | Exposure frequency (EF) |
| Wu et al. (2015) | Children: 20  Teens: 46.25  Adults: 70 | Kg | Body weight (kg) |
| Smith (1994) | Children: 4  Teens: 13  Adults: 40 | year | Exposure Duration |
|  | Children: 1460  Teens: 4745  Adults: 14600 | days | Averaging Time (day*365) |
| IRIS (2016) | 0.06 | mg/kg/day | Oral reference dose (RfD) |

The chronic daily intake (CDI) values of fluoride via drinking water can be calculated for different age groups according to USEPA method following Supp. Equation (Supp. Eq. 1):

$CDI=\frac{Cw \times IRw \times EF \times ED}{BW \times AT}$ (1)

HQ, which represents the non-carcinogenicity risk through different exposure pathways, can be calculated using estimated daily intake (EDI) and oral reference dose (RfD) using the following Supp. Equation 2 (Supp. Eq. 2):

$HQ=\frac{\mathrm{CDI}}{\mathrm{RfD}}$ (2)

**All input parameters of the non-carcinogenic risk assessment are shown in Supp. Table S7.**

To assess the effect of climate on oral fluoride intake and the subsequent hazard quotient (HQ), we considered two regions in Iran with the same assumed fluoride concentration but different climates of hot and cold. The fluoride concentration in both regions was assumed to be identical at 1.5 mg/L. The only difference between the two regions was their climate, with the warm region having higher water ingestion rate, based on data obtained from two real studies conducted in Iran (Bazeli et al., 2022; Mohammadpour et al., 2022). Calculations were performed only for the adult age group. Based on this calculation, the CDI values in the cold and warm regions were 0.026357143 and 0.041785714, respectively, and the HQ values in the cold and warm regions were 0.439285714 and 0.696428571, respectively. As can be seen, despite the same assumed fluoride concentration in both regions, the intake dose and health risk were higher in the warmer region due to higher daily water consumption.

**References**

Akpabio, S. P., Gardiner, J. H., & Adeyinka, A. (1984). *Dental Epidemiological and Orthodontic Survey-Nigeria, 1982*.

Akpata, E. S., Danfillo, I. S., Otoh, E. C., & Mafeni, J. O. (2009). Geographical mapping of fluoride levels in drinking water sources in Nigeria. *African Health Sciences, 9*(4), 227-233.

Alam, W., Gyanendra, Y., Neihsial, M. T., & Nesa, N. (2019). Hydrogeochemical assessment of groundwater arsenic and fluoride contamination in Thoubal District, Manipur, India. *Earth Science India, 12*, 38–52. <https://doi.org/10.31870/ESI.12.1.2019.03>

Alam, W., Gyanendra, Y., Chanda, R., Laishram, R. J., & Nesa, N. (2020). Hydrogeochemical assessment and evaluation of groundwater quality in selected areas of Bishnupur District, Manipur. *Journal of the Geological Society of India, 96*, 272–278. <https://doi.org/10.1007/S12594-020-1547-4>

Alam, W., Singh, K. S., Gyanendra, Y., Laishram, R. J., & Nesa, N. (2020). Hydrogeochemical assessment of groundwater quality for a few habitations of Chandel District, Manipur (India). *Applied Water Science, 10*, 123. <https://doi.org/10.1007/s13201-020-01208-0>

Alam, W., Zamminsion, K., Gyanendra, Y., Laishram, R. J., & Nesa, N. (2022). Geochemical and multivariate assessment of groundwater resources of Churachandpur subdivision of Manipur, India. *Applied Water Science, 12*, 111. <https://doi.org/10.1007/S13201-022-01638-Y>

Alfredo, K. A., Lawler, D. F., & Katz, L. E. (2014). Fluoride contamination in the Bongo District of Ghana, West Africa: Geogenic contamination and cultural complexities. *Water International, 39*(4), 486–503. <https://doi.org/10.1080/02508060.2014.926234>

Aminu, T., & Amadi, A. N. (2014). Fluoride contamination of shallow groundwater in parts of Zango Local Government Area of Katsina State, Northwest Nigeria. *Environmental Science, 2*(5), 178–185. <https://doi.org/10.12691/jgg-2-5-1>

Anornu, G., Gibrilla, A., & Adomako, D. (2017). Tracking nitrate sources in groundwater and associated health risk for rural communities in the White Volta River Basin of Ghana using isotopic approach. *Science of the Total Environment, 603–604*, 687–698. <https://doi.org/10.1016/J.SCITOTENV.2017.01.219>

Apambire, W. B., Boyle, D. R., & Michel, F. A. (1997). Geochemistry, genesis, and health implications of fluoriferous groundwaters in the upper regions of Ghana. *Environmental Geology, 33*(1), 13–24. <https://doi.org/10.1007/S002540050221>

Aqeel, A., Al-Amry, A., & Alharbi, O. (2017). Assessment and geospatial distribution mapping of fluoride concentrations in the groundwater of Al-Howban Basin, Taiz, Yemen. *Arabian Journal of Geosciences, 10*(14), 312. <https://doi.org/10.1007/S12517-017-3069-Y>

Atipoka, F. A. (2009). Water supply challenges in rural Ghana. *Desalination, 248*(1–3), 212–217. <https://doi.org/10.1016/J.DESAL.2008.05.057>

Bazeli, J., Ghalehaskar, S., Morovati, M., Soleimani, H., Masoumi, S., Rahmani Sani, A., Saghi, M. H., & Rastegar, A. (2022). Health risk assessment techniques to evaluate non-carcinogenic human health risk due to fluoride, nitrite and nitrate using Monte Carlo simulation and sensitivity analysis in groundwater of Khaf County, Iran. *International Journal of Environmental Analytical Chemistry, 102*(8), 1793–1813. <https://doi.org/10.1080/03067319.2020.1743280>

Bhattacharya, P., Adhikari, S., Samal, A. C., Das, R., Dey, D., Deb, A., Ahmed, S., Hussein, J., De, A., Das, A., Joardar, M., Panigrahi, A. K., Roychowdhury, T., & Santra, S. C. (2020). Health risk assessment of co-occurrence of toxic fluoride and arsenic in groundwater of Dharmanagar region, North Tripura, India. *Groundwater for Sustainable Development, 11*, 100430. <https://doi.org/10.1016/J.GSD.2020.100430>

BGS-DPHE (2001). Arsenic contamination of groundwater in Bangladesh. British Geological Survey and Department of Public Health Engineering, Government of Bangladesh. [British Geological Survey and Department of Public Health Engineering, Government of Bangladesh]

Bright, I. C., & Chibuzo, A. A. (2018). Analysis of fluoride concentration in commercial sachet water brands in Enugu, Nigeria. *Advances in Analytical Chemistry, 8*(1), 10–14. <https://doi.org/10.5923/j.aac.20180801.03>

Brindha, K., & Elango, L. (2013). Geochemistry of fluoride-rich groundwater in a weathered granitic rock region, southern India. *Water Quality, Exposure and Health, 5*(3), 127–138. <https://doi.org/10.1007/S12403-013-0096-0>

Chakrabarty, S., & Sarma, H. P. (2011). Heavy metal contamination of drinking water in Kamrup District, Assam, India. *Environmental Monitoring and Assessment, 179*(1–4), 479–486. <https://doi.org/10.1007/s10661-010-1750-7>

Craig, L., Lutz, A., Berry, K. A., & Yang, W. (2015). Recommendations for fluoride limits in drinking water based on estimated daily fluoride intake in the Upper East Region, Ghana. *Science of the Total Environment, 532*, 127–137. <https://doi.org/10.1016/J.SCITOTENV.2015.05.126>

Craig, L., Thomas, J. M., Lutz, A., & Decker, D. L. (2018). Determining the optimum locations for pumping low-fluoride groundwater to distribute to communities in a fluoridic area in the Upper East Region, Ghana. *Chemical Geology, 476*, 481–492. <https://doi.org/10.1016/J.CHEMGEO.2017.12.001>

Da Conceição, F. T., & Bonotto, D. M. (2006). Radionuclides, heavy metals and fluorine incidence at Tapira phosphate rocks, Brazil, and their industrial by-products. *Environmental Pollution, 139*(2), 232–243. <https://doi.org/10.1016/J.ENVPOL.2005.05.014>

Dartan, G., Taşpınar, F., & Toroz, Ä. (2017). Analysis of fluoride pollution from fertilizer industry and phosphogypsum piles in agricultural area. *Journal of Industrial Pollution Control, 33(1)*, 662–669.

Devi, S. B., & Kamble, R. K. (2006). Groundwater fluoride in East Imphal District of Manipur. *Indian Journal of Environmental Protection (IJEP), 26*(10), 885–891.

Dibal, H. U., & Lar, U. A. (2005). Preliminary survey of fluoride concentrations in the groundwaters of Kaltungo Town and environs, Gombe State, Northeastern Nigeria: Water quality and health implications. *Journal of Environmental Sciences, 9*.

Dibal, H. U., Schoeneich, K., Garba, I., Lar, U. A., & Bala, E. A. (2012). Occurrence of fluoride in the drinking waters of Langtang area, north central Nigeria. *Health, 4*(11), 1116–1126. <https://doi.org/10.4236/health.2012.411169>

Dirisu, C. G., Mafiana, M. O., Okwodu, N. E., & Isaac, A. (2016). Fluoride contents of community drinking water: Biological and public health implications. *American Journal of Water Resources, 4*(3), 54–57. <https://doi.org/10.12691/AJWR-4-3-1>

Dutta, S., Barman, R., Rabha, D., Raj, R., & Radhapyari, K. (2020). Uranium, heavy metals and fluoride co-occurrence in groundwater of Kamrup Metropolitan District of Assam, India. *Bhujal News, 30*(1–4), 34–50.

Egbinola, C. N., & Amanambu, A. C. (2014). Groundwater contamination in Ibadan, South-West Nigeria. *SpringerPlus, 3*(1), 448. <https://doi.org/10.1186/2193-1801-3-448>

Emenike, C. P. G., Tenebe, I. T., & Jarvis, P. (2018). Fluoride contamination in groundwater sources in Southwestern Nigeria: Assessment using multivariate statistical approach and human health risk. *Ecotoxicology and Environmental Safety, 156*, 391–402. <https://doi.org/10.1016/j.ecoenv.2018.03.022>

Farooqi, A., Masuda, H., Kusakabe, M., Naseem, M., & Firdous, N. (2007). Distribution of highly arsenic- and fluoride-contaminated groundwater from east Punjab, Pakistan, and the controlling role of anthropogenic pollutants in the natural hydrological cycle. *Geochemical Journal, 41*(4), 213–234. <https://doi.org/10.2343/geochemj.41.213>

Firempong, C., Nsiah, K., Awunyo-Vitor, D., & Dongsogo, J. (2013). Soluble fluoride levels in drinking water: A major risk factor of dental fluorosis among children in Bongo community of Ghana. *Ghana Medical Journal, 47*(1), 16.

Ganyaglo, S. Y., Gibrilla, A., Teye, E. M., Owusu-Ansah, E. D. G. J., Tettey, S., Diabene, P. Y., & Asimah, S. (2019). Groundwater fluoride contamination and probabilistic health risk assessment in fluoride endemic areas of the Upper East Region, Ghana. *Chemosphere, 233*, 862–872. <https://doi.org/10.1016/j.chemosphere.2019.05.276>

Giwa, A. S., Memon, A. G., Ahmad, J., Ismail, T., Abbasi, S. A., Kamran, K., Wang, B., Segun, B., & Seydou, H. (2021). Assessment of high fluoride in water sources and endemic fluorosis in the north-eastern communities of Gombe State, Nigeria. *Environmental Pollutants and Bioavailability, 33*(1), 31–40. <https://doi.org/10.1080/26395940.2021.1908849>

Gogoi, R. R., Khanikar, L., Gogoi, J., Neog, N., Deka, D. J., & Sarma, K. P. (2021). Geochemical sources, hydrogeochemical behaviour of fluoride release and its health risk assessment in some fluorosis endemic areas of the Brahmaputra Valley of Assam, India. *Applied Geochemistry, 127*, 104911. <https://doi.org/10.1016/j.apgeochem.2021.104911>

Goyit, M. P., Solomon, O. A., & Kutshik, R. J. (2018). Distribution of fluoride in surface and groundwater: A case study of Langtang North, Plateau State, Nigeria. *International Journal of Biological and Chemical Sciences, 12*(2), 1057–1067. <https://doi.org/10.4314/ijbcs.v12i2.33>

Hanse, A., Chabukdhara, M., Gohain Baruah, S., Boruah, H., & Gupta, S. K. (2019). Fluoride contamination in groundwater and associated health risks in Karbi Anglong District, Assam, Northeast India. *Environmental Monitoring and Assessment, 191*(12), 1–17. <https://doi.org/10.1007/s10661-019-7970-6>

He, X., Li, P., Ji, Y., Wang, Y., Su, Z., & Elumalai, V. (2020). Groundwater arsenic and fluoride and associated arsenicosis and fluorosis in China: Occurrence, distribution and management. *Exposure and Health, 12*(3), 355–368. <https://doi.org/10.1007/s12403-020-00347-8>

Hyeladi, D., Dahilo, S. A., Raymond, D., & Uriah, L. (2014). Low-level fluoride content in groundwater of the younger granite aquifers in parts of Jos Plateau, Nigeria. *American Journal of Environmental Protection, 9*(5), 42–53. <https://doi.org/10.11648/j.ajep.s.2014030602.17>

Ibe, K., Adlegbembo, A. O., Mafeni, J., & Danfillo, I. (1999). Natural fluoride levels in some springs and streams from the Late Maastrichtian Ajali Formation in the Ohafia–Arochukwu area of southeastern Nigeria. Odontostomatol Trop. 22: 41–45.

Ijumulana, J., Ligate, F., Irunde, R., Bhattacharya, P., Ahmad, A., Tomašek, I., Maity, J. P., & Mtalo, F. (2022). Spatial variability of the sources and distribution of fluoride in groundwater of the Sanya alluvial plain aquifers in northern Tanzania. *Science of the Total Environment, 810*, 152153. <https://doi.org/10.1016/j.scitotenv.2021.152153>

Kakoty, P., Barooah, P., Baruah, M. K., Goswami, A., Borah, G. C., Gogoi, H. M., Ahmed, F., Gogoi, A., & Paul, A. B. (2008). Fluoride and endemic fluorosis in Karbi Anglong District of Assam, India. *Fluoride, 41*(1), 42–45.

Kloos, H., & Tekle Haimanot, R. (1999). Distribution of fluoride and fluorosis in Ethiopia and prospects for control. *Tropical Medicine & International Health, 4*(5), 355–364. <https://doi.org/10.1046/j.1365-3156.1999.00405.x>

Koślacz, R. (1989). Fluor w neogeńskich poziomach wodonośnych rejonu Nysy [Fluoride in the Neogene aquifers in the Nysa region]. In *VI Polish–Czechoslovak Symposium: Protecting and Pollution of Groundwater* (pp. 209–216).

Lalbiakmawia, F., & Vanthangliana, V. (2015). Application of geo-spatial technologies for groundwater quality mapping of Aizawl District, Mizoram, India. *Science Vision, 15*(3), 115–123.

Lar, U. A., Daspan, R., Dibal, H. U., & Jaryum, S. W. (2007). Fluoride occurrence in the surface and groundwater of Fobur area of Jos East LGA of Plateau State, Nigeria. *Journal of Environmental Sciences, II* (2), 99–105.

Lar, U. A., & Gusikit, R. B. (2015). Environmental and health impact of potentially harmful elements distribution in the Panyam (Sura) volcanic province, Jos Plateau, central Nigeria. *Environmental Earth Sciences, 74*(2), 1699–1710. <https://doi.org/10.1007/s12665-015-4178-0>

MINSALUD (2016). Documento técnico: Política de flúor vs. caries y fluorosis dental en Colombia. Ministerio de Salud y Protección Social, Colombia. Versión 3, febrero 2016. [Technical document: Fluoride policy vs. dental caries and fluorosis in Colombia. Ministry of Health and Social Protection, Colombia. Version 3, February 2016]

Mirlean, N., & Roisenberg, A. (2007). Fluoride distribution in the environment along the gradient of a phosphate-fertilizer production emission (southern Brazil). *Environmental Geochemistry and Health, 29*(3), 179–187. <https://doi.org/10.1007/s10653-006-9061-1>

Mohammadpour, A., Tabatabaee, Z., Dehbandi, R., Khaksefidi, R., Golaki, M., Gharechahi, E., Samaei, M. R., Mohammadpour, R., Sheibani, A., & Badeenezhad, A. (2022). Concentration, distribution and probabilistic health risk assessment of exposure to fluoride in drinking water of Hormozgan province, Iran. *Stochastic Environmental Research and Risk Assessment, 36*(4), 1035–1047. <https://doi.org/10.1007/s00477-021-02090-1>

Ndukwe, V., Uzoegbu, N., & Agibe. (2019). Assessment of some physicochemical properties of groundwater quality in Ikwuano region of southeastern Nigeria. *Journal of Applied Science and Environmental Management, 23*(8), 1595–1602. <https://doi.org/10.4314/jasem.v23i8.26>

Natural Water Resources Authority of Taiz (NWRA/Taiz). (2008). Technical notice on quality of water in Hidhran and Al Burahay basin, Taiz, Yemen. Taiz, Yemen.

Ogbu, I. S. I., Okoro, O. I. O., & Ugwuja, E. I. (2012). Well water fluoride in Enugu, Nigeria. *The International Journal of Occupational and Environmental Medicine, 3*, 96–98.

Okunlola, I. A., Amadi, A. N., Olashinde, P. I., Maspalma, S. S., & Okoye, N. O. (2016). Quality assessment of groundwater from shallow aquifers in Hong area, Adamawa State, northeastern Nigeria. *Ife Journal of Science, 18*(1), 267–283.

Olasehinde, P., Amadi, A. N., Okunlola, I., Dan-Hassan, M. A., & Jimoh, M. O. (2016). Occurrence of fluoride and some heavy metals in groundwater from shallow aquifers near Ogbomosho, north-central Nigeria. *Journal of Natural Sciences Research*, 6(13), 55-60.

Oteze, G. E., & Ayegbusi, M. S. (2002). The continental terminal aquifer in northeast Nigeria. *Journal of the Nigerian Association of Hydrogeologists, 13*, 62–69.

Pamei, M., Naresh, G., Dutta, D., & Puzari, A. (2020). Accessibility of safe drinking water in the greater Dimapur area of Nagaland and related health hazards: An analytical study. *International Journal of Energy and Water Resources, 4*(3), 245–255. <https://doi.org/10.1007/s42108-020-00074-5>

Podgorski, J., & Berg, M. (2022). Global analysis and prediction of fluoride in groundwater. *Nature Communications, 13*(1), 1–9. <https://doi.org/10.1038/s41467-022-31940-x>

Ransley, T. R., Radke, B. M., Feitz, A. J., Kellett, J. R., Owens, R., Bell, J., Stewart, G., & Carey, H. (2015). *Hydrogeological atlas of the Great Artesian Basin*. Geoscience Australia. <https://doi.org/10.11636/9781925124668>

Rao, N. S. (1997). The occurrence and behaviour of fluoride in the groundwater of the Lower Vamsadhara River Basin, India. *Hydrological Sciences Journal, 42*(6), 877–892. <https://doi.org/10.1080/02626669709492085>

Sahadevan, S., & Chandrasekharam, D. (2008). High fluoride groundwater of Karbi-Anglong District, Assam, northeastern India: Source characterization. In P. Bhattacharya, A. Ramanathan, A. B. Mukherjee, J. Bundschuh, D. Chandrasekharam, & A. K. Keshari (Eds.), *Groundwater for sustainable development: Problems, perspectives and challenges* (pp. 325-334). CRC Press. <https://doi.org/10.1201/9780203894569-37>

Salifu, A., Petrusevski, B., Mwampashi, E. S., Pazi, I. A., Ghebremichael, K., Buamah, R., Aubry, C., Amy, G. L., & Kennedy, M. D. (2016). Defluoridation of groundwater using aluminum-coated bauxite: Optimization of synthesis process conditions and equilibrium study. *Journal of Environmental Management, 181*, 108–117. <https://doi.org/10.1016/j.jenvman.2016.06.011>

Sharma, P., Sarma, H. P., & Mahanta, C. (2012). Evaluation of groundwater quality with emphasis on fluoride concentration in Nalbari District, Assam, northeast India. *Environmental Earth Sciences, 65*(7), 2147–2159. <https://doi.org/10.1007/s12665-011-1195-5>

Smith, R. L. (1994). Use of Monte Carlo simulation for human exposure assessment at a Superfund site. *Risk Analysis, 14*(4), 433–439. <https://doi.org/10.1111/j.1539-6924.1994.tb00261.x>

Sunkari, E. D., Abangba, T., Ewusi, A., Tetteh, S. E. K., & Ofosu, E. (2023). Hydrogeochemical evolution and assessment of groundwater quality for drinking and irrigation purposes in the Gushegu Municipality and parts of East Mamprusi District, Ghana. *Environmental Monitoring and Assessment, 195*(165), 1–25. <https://doi.org/10.1007/s10661-022-10731-3>

Sunkari, E. D., & Abu, M. (2019). Hydrochemistry with special reference to fluoride contamination in groundwater of the Bongo District, Upper East Region, Ghana. *Sustainable Water Resources Management, 5*(4), 1803–1814. <https://doi.org/10.1007/s40899-019-00335-0>

Sunkari, E. D., Abu, M., Zango, M. S., & Lomoro Wani, A. M. (2020). Hydrogeochemical characterization and assessment of groundwater quality in the Kwahu-Bombouaka Group of the Voltaian Supergroup, Ghana. *Journal of African Earth Sciences, 169*, 103899. <https://doi.org/10.1016/j.jafrearsci.2020.103899>

Sunkari, E. D., Adams, S. J., Okyere, M. B., & Bhattacharya, P. (2022). Groundwater fluoride contamination in Ghana and the associated human health risks: Any sustainable mitigation measures to curtail the long-term hazards? *Groundwater for Sustainable Development, 16*, 100715. <https://doi.org/10.1016/j.gsd.2021.100715>

Sunkari, E. D., Zango, M. S., & Korboe, H. M. (2018). Comparative analysis of fluoride concentrations in groundwaters in northern and southern Ghana: Implications for contaminant sources. *Earth Systems and Environment, 2*(1), 103–117. <https://doi.org/10.1007/s41748-018-0044-z>

Tay, C. K. (2017). Hydrogeochemical framework and factor analysis of fluoride contamination in groundwater within the Savelugu–Nanton District, northern Ghana. *West African Journal of Applied Ecology, 25*(1), 33–55.

Thompson, J.H. (1958). The geology and hydrogeology of Gombe, Bauchi Province. Records of the Geology Survey of Nigeria, 46-56.

Uriah, L. A., Hyeladi, D., & Krzyszto, S. F. (2014). Fluoride in groundwater in Nigeria: Origin and human health impact. *American Journal of Environmental Protection, 3*(6-2), 66–69. <https://doi.org/10.11648/j.ajep.s.2014030602.19>

U.S. Environmental Protection Agency. (2004). *Risk assessment guidance for Superfund: Volume I—Human health evaluation manual (Part E: Supplemental guidance for dermal risk assessment)* (EPA/540/R/99/005). <https://www.epa.gov>

U.S. Environmental Protection Agency, Integrated Risk Information System (IRIS). (2016). *Fluorine (soluble fluoride).* <https://cfpub.epa.gov/ncea/iris2/chemicalLanding.cfm?substance_nmbr=53>

Wilson, D. C. (1954). Fluorine content of some Nigerian waters. *Nature, 173*(4398), 305. <https://doi.org/10.1038/173305a0>

Wu, J., Li, P., & Qian, H. (2015). Hydrochemical characterization of drinking groundwater with special reference to fluoride in an arid area of China and the control of aquifer leakage on its concentrations. *Environmental Earth Sciences, 73*(12), 8575–8588. <https://doi.org/10.1007/s12665-015-4018-2>

Zahid, A., Hassan, M. Q., & Ahmed, K. M. U. (2015). Simulation of flowpaths and travel time of groundwater through arsenic-contaminated zones in the multilayered aquifer system of the Bengal Basin. *Environmental Earth Sciences, 73*(3), 979–991. <https://doi.org/10.1007/s12665-014-3447-7>

Zango, M. S., Pelig-Ba, K. B., Anim-Gyampo, M., Gibrilla, A., & Sunkari, E. D. (2021). Hydrogeochemical and isotopic controls on the source of fluoride in groundwater within the Vea Catchment, northeastern Ghana. *Groundwater for Sustainable Development, 12*, 100526. <https://doi.org/10.1016/j.gsd.2020.100526>

Zango, M. S., Sunkari, E. D., Abu, M., & Lermi, A. (2019). Hydrogeochemical controls and human health risk assessment of groundwater fluoride and boron in the semi-arid northeast region of Ghana. *Journal of Geochemical Exploration, 207*, 106363. <https://doi.org/10.1016/j.gexplo.2019.106363>

Top of Form
